# Supplementary material for: A meta-analysis of the association between inflammatory cytokine polymorphism and neonatal sepsis
Source: PLoS One. 2024 Jun 7;19(6):e0301859. doi: 10.1371/journal.pone.0301859 (PMC11161124; doi:10.1371/journal.pone.0301859)
Supplement: S1 File — (DOC) [file pone.0301859.s001.doc]

Excluded Articles：

IL-1

**Duplicates(77):**

1. Genetic variants of TNF-[FC12]a, IL-1beta, IL-4 receptor [FC12]a-chain, IL-6 and IL-10 genes are not risk factors for sepsis in low-birth-weight infants.
2. Genetic variants of TNF-α, IL-1β, IL-4 receptor α-chain, IL-6 and IL-10 genes are not risk factors for sepsis in low-birth-weight infants.
3. Association between IL-1ra gene polymorphism and premature delivery.
4. Association between IL-1ra gene polymorphism and premature delivery.
5. Cytokine responses and sudden infant death syndrome: genetic, developmental, and environmental risk factors.
6. Cytokine responses and sudden infant death syndrome: genetic, developmental, and environmental risk factors.
7. Genetic polymorphisms and risk for acute renal failure in preterm neonates.
8. Genetic polymorphisms and risk for acute renal failure in preterm neonates.
9. Interleukin-10 and its role in clinical immunoparalysis following pediatric cardiac surgery.
10. Interleukin-10 and its role in clinical immunoparalysis following pediatric cardiac surgery.
11. IL-10, IL-6 and CD14 polymorphisms and sepsis outcome in ventilated very low birth weight infants.
12. IL-10, IL-6 and CD14 polymorphisms and sepsis outcome in ventilated very low birth weight infants.
13. Effect of various genetic polymorphisms on the incidence and outcome of severe sepsis.
14. Effect of various genetic polymorphisms on the incidence and outcome of severe sepsis.
15. Genetic polymorphisms of CD14, toll-like receptor 4, and caspase-recruitment domain 15 are not associated with necrotizing enterocolitis in very low birth weight infants.
16. Genetic basis for necrotizing enterocolitis - risk factors and their relations to genetic polymorphisms.
17. Genetic basis for necrotizing enterocolitis - risk factors and their relations to genetic polymorphisms.
18. Molecular biology on the ICU. From understanding to treating sepsis.
19. The correlation between systemic inflammatory response syndrome in newborns and IL-10 gene polymorphism.
20. Genetic association studies in VLBW infants exemplifying susceptibility to sepsis--recent findings and implications for future research.
21. Interleukin-6 gene variants and the risk of sepsis development in children.
22. Interleukin-6 gene variants and the risk of sepsis development in children.
23. Interleukin-6 (-174C) polymorphism and the risk of sepsis in very low birth weight infants: meta-analysis.
24. Interleukin-6 (-174C) polymorphism and the risk of sepsis in very low birth weight infants: meta-analysis.
25. Interleukin-6 polymorphism is associated with chorioamnionitis and neonatal infections in preterm infants.
26. Interleukin-6 polymorphism is associated with chorioamnionitis and neonatal infections in preterm infants.
27. [Genetic markers of predisposition to infectious complications in neonatal infants with respiratory distress syndrome.]
28. Role of Polymorphic Variants as Genetic Modulators of Infection in Neonatal Sepsis.
29. Role of Polymorphic Variants as Genetic Modulators of Infection in Neonatal Sepsis.
30. An age-related decrease in factor V Leiden frequency among Polish subjects.
31. An age-related decrease in factor V Leiden frequency among Polish subjects.
32. Human cytomegalovirus UL144 is associated with viremia and infant development sequelae in congenital infection.
33. Association between bronchopulmonary dysplasia and MBL2 and IL1-RN polymorphisms.
34. Association between bronchopulmonary dysplasia and MBL2 and IL1-RN polymorphisms.
35. Toll-like 4 receptor variant, Asp299Gly, and reduced risk of hemorrhagic cystitis after hematopoietic stem cell transplantation.
36. Frequencies of functional caspase 12 genotypes in the North Africa population.
37. Genetic association study of tumor necrosis factor-alpha with sepsis and septic shock in Thai pediatric patients.
38. Genetic association study of tumor necrosis factor-alpha with sepsis and septic shock in Thai pediatric patients.
39. Characterization of the acute phase response in critically ill children.
40. Characterization of the acute phase response in critically ill children.
41. Prediction of sepsis-related outcomes in neonates through systematic genotyping of polymorphisms in genes for innate immunity and inflammation: a narrative review and critical perspective.
42. Prediction of sepsis-related outcomes in neonates through systematic genotyping of polymorphisms in genes for innate immunity and inflammation: a narrative review and critical perspective.
43. Neonatal infections in Saudi Arabia: association with C-reactive protein, CRP -286 (C>T>A) gene polymorphism and IgG antibodies.
44. Prognostic markers of pediatric meningococcal sepsis.
45. Prognostic markers of pediatric meningococcal sepsis.
46. Multiple gene-to-gene interactions in children with sepsis: a combination of five gene variants predicts outcome of life-threatening sepsis.
47. Multiple gene-to-gene interactions in children with sepsis: a combination of five gene variants predicts outcome of life-threatening sepsis.
48. Neonatal infections in Saudi Arabia: Association with cytokine gene polymorphisms.
49. Association of Polymorphisms in IRAK1, IRAK4 and MyD88, and Severe Invasive Pneumococcal Disease.
50. Research progress on heart rate variability in neonatal sepsis.
51. Genome-wide association study of sepsis in extremely premature infants.
52. Systematic Review and Meta-analysis: Gene Association Studies in Neonatal Sepsis.
53. Systematic Review and Meta-analysis: Gene Association Studies in Neonatal Sepsis.
54. Tumor necrosis factor-α-308G/A and-238G/A polymorphisms are associated with increased risks of sepsis: evidence from an updated meta-analysis.
55. Tumor necrosis factor-α-308G/A and-238G/A polymorphisms are associated with increased risks of sepsis: evidence from an updated meta-analysis.
56. Risk of nontyphoidal <i>Salmonella</i> bacteraemia in African children is modified by <i>STAT4</i>.
57. Research progress in the treatment of alcoholic liver disease based on the hepatointestinal axis.
58. The association between interleukin-6 gene-174G/C single nucleotide polymorphism and sepsis: an updated meta-analysis with trial sequential analysis.
59. The association between interleukin-6 gene-174G/C single nucleotide polymorphism and sepsis: an updated meta-analysis with trial sequential analysis.
60. Association of gene polymorphism of bactericidal permeability increasing protein rs4358188, cluster of differentiation 14 rs2569190, interleukin 1β rs1143643 and matrix metalloproteinase-16 rs2664349 with neonatal sepsis.
61. Association between Tumor Necrosis Factor-α Promoter-308 G/A Polymorphism and Early Onset Sepsis in Preterm Infants.
62. Association between Tumor Necrosis Factor-α Promoter-308 G/A Polymorphism and Early Onset Sepsis in Preterm Infants.
63. Research progress on the relationship between susceptibility to neonatal sepsis and polymorphisms of tumor necrosis factor and interleukin genes.
64. Research progress on the relationship between susceptibility to neonatal sepsis and polymorphisms of tumor necrosis factor and interleukin genes.
65. Proprotein Convertase Subtilisin/Kexin Type 9 Loss-of-Function Is Detrimental to the Juvenile Host With Septic Shock*.
66. Genetic variant rs16944 in <i>IL1B</i> gene is a risk factor for early-onset sepsis susceptibility and outcome in preterm infants.
67. [Association between interleukin-8 rs4073 polymorphisms and susceptibility to neonatal sepsis.]
68. IL-1 β A study on the correlation between gene single nucleotide polymorphism and sepsis in full-term newborns.
69. IL-1 β A study on the correlation between gene single nucleotide polymorphism and sepsis in full-term newborns.
70. The relationship between the polymorphism of rs4073 locus in 68-IL-8 gene and susceptibility to neonatal sepsis.
71. The relationship between the polymorphism of rs4073 locus in 68-IL-8 gene and susceptibility to neonatal sepsis.
72. The relationship between the polymorphism of rs4073 locus in 68-IL-8 gene and susceptibility to neonatal sepsis.
73. Association of IL-6 -174G > C Polymorphism with Susceptibility to Childhood Sepsis: A Systematic Review and Meta-Analysis
74. Association between Interleukin-6 rs1800795 Polymorphism and Serum Interleukin-6 Levels and Full-Term Neonatal Sepsis.
75. The impact and interaction of TLR2 and IRF-5 gene polymorphisms on susceptibility to neonatal sepsis.
76. The correlation between 74-IL-10 gene polymorphism and susceptibility to sepsis in full-term newborns.
77. The correlation between 74-IL-10 gene polymorphism and susceptibility to sepsis in full-term newborns.

**Review,systematic analysis, animal experiment（5）：**

1. Association between lymphotoxin-α intron +252 polymorphism and sepsis: a meta-analysis.
2. Association between IL-6-174G/C Polymorphism and the Risk of Sepsis and Mortality: A Systematic Review and Meta-Analysis.
3. Periodontitis and gestational diabetes mellitus: a systematic review and meta-analysis of observational studies.
4. Prenatal pesticide exposure associated with glycated haemoglobin and markers of metabolic dysfunction in adolescents.
5. Association of IL-6-174G &gt; C Polymorphism with Susceptibility to Childhood Sepsis: A Systematic Review and Meta-Analysis.

**Summarize(4)：**

1. Molecular biology on the ICU - From understanding to treating sepsis.
2. Prediction of sepsis-related outcomes in neonates through systematic genotyping of polymorphisms in genes for innate immunity and inflammation: a narrative review and critical perspective
3. Elucidating the role of genomics in neonatal sepsis.
4. Research progress on the relationship between susceptibility to neonatal sepsis and polymorphisms of tumor necrosis factor and interleukin genes.

**Studies that include on the title and abstract either having no connection with the subject（142）：**

1. 4G/5G promoter polymorphism in the plasminogen-activator-inhibitor-1 gene and outcome of meningococcal disease. Meningococcal Research Group.
2. Pilot study assessing TNF gene polymorphism as a prognostic marker for disease progression in neonates with sepsis.
3. A family with delayed Bruton syndrome.
4. Severity of meningococcal disease in children and the angiotensin-converting enzyme insertion/deletion polymorphism.
5. Bench-to-bedside review: genetic influences on meningococcal disease.
6. Is interleukin-6 -174 genotype associated with the development of septicemia in preterm infants?
7. Lower prevalence of IL-4 receptor alpha-chain gene G variant in very-low-birth-weight infants with necrotizing enterocolitis.
8. Mutations of genes involved in the innate immune system as predictors of sepsis in very low birth weight infants.
9. The role of molecular genetics in the pathogenesis and diagnosis of neonatal sepsis.
10. Polymorphisms of genes involved in innate immunity: association with preterm delivery.
11. Tumor necrosis factor alpha -- 308 polymorphism associated with increased sepsis mortality in ventilated very low birth weight infants.
12. Evaluation of systemic inflammatory responses in neonates with herpes simplex virus infection.
13. Genetic screening for susceptibility to infection in the NICU setting.
14. Virulence and cord blood mononuclear cells cytokine production induced by perinatal listeria monocytogenes strains from different phylogenetic lineages.
15. Association of two tumour necrosis factor gene polymorphisms with the incidence of severe intraventricular haemorrhage in preterm infants.
16. Periodontal therapy reduces the rate of preterm low birth weight in women with pregnancy-associated gingivitis.
17. The correlation between systemic inflammatory response syndrome in newborns and IL-10 gene polymorphism.
18. A macrophage migration inhibitory factor promoter polymorphism is associated with high-density parasitemia in children with malaria.
19. Interleukin-6-174-genotype, sepsis and cerebral injury in very low birth weight infants
20. Genetic polymorphisms and infections.
21. Prevalence of two tumor necrosis factor gene polymorphisms in premature infants with early onset sepsis.
22. Genetic Polymorphisms of CD14, toll-like receptor 4, and caspase-recruitment domain 15 are not associated with necrotizing enterocolitis in very low birth weight infants.
23. 4G4G genotype of the plasminogen activator inhibitor-1 promoter polymorphism associates with disseminated intravascular coagulation in children with systemic meningococcemia.
24. Genetic association studies in VLBW infants exemplifying susceptibility to sepsis -: recent findings and implications for future research.
25. Acute inflammation is exacerbated in mice genetically predisposed to a severe protein C deficiency.
26. Investigation of the Role of Pen-Like Receptors in the Development of Immunopathogenic Intestinal Diseases.
27. The extent to which genotype information may add to the prediction of distrurbed perinatal adaption:: none, minor, or major?
28. Sudden infant death syndrome: Review of implicated genetic factors.
29. Basic and clinical research on retinopathy of premature infants.
30. Role of angiotensin-converting enzyme gene polymorphisms in children with sepsis and septic shock.
31. The role of mannose-binding lectin in susceptibility to infection in preterm Neonates.
32. A study on risk factors associated with PROM and methods for detection of BV flora.
33. 159C&gt;T CD14 genotype -: Functional effects on innate immune responses in term neonates.
34. An infectious aetiology of sudden infant death syndrome.
35. Susceptibility to meningococcal infection-Nature versus nurture?
36. Genetic polymorphisms of IL-6-174 and IL-10-1082 in full term neonates with late onset blood stream infections.
37. [Genetic markers of predisposition to infectious complications in neonatal infants with respiratory distress syndrome.]
38. Genetics of susceptibility to malaria related phenotypes.
39. Immaturity, perinatal inflammation, and retinopathy of prematurity: A multi-hit hypothesis
40. Effects of RANTES and MBL2 gene polymorphisms in sickle cell disease clinical outcomes: association of the g.In1.1T>C RANTES variant with protection against infections.
41. Cytokine gene polymorphisms in preterm infants with necrotising enterocolitis: genetic association study.
42. Lack of association between TLR4 polymorphism and severe gram-negative bacterial infection in neonates.
43. Nitric oxide synthase 2A (NOS2A) polymorphisms are not associated with invasive pneumococcal disease.
44. Interleukin-6 G(-174)C polymorphism is associated with mental retardation in cystic periventricular leucomalacia in preterm infants.
45. Theme Index of Chinese Journal of Contemporary Pediatrics, Volume 12, 2010.
46. Genetic variability in complement activation modulates the systemic inflammatory response syndrome in children.
47. Genetic polymorphisms in the endotoxin receptor may influence platelet count as part of the acute phase response in critically ill children.
48. [Correlations between serum interleukin-18 (IL-18) level, IL-18 gene promoter polymorphisms and the development of sepsis in children.]
49. Common <i>NFKBIL2</i> polymorphisms and susceptibility to pneumococcal disease: a genetic association study.
50. Association of the+874 T/A interferon gamma polymorphism with infections in sickle cell disease.
51. CCR5, RANTES and SDF-1 polymorphisms and mother-to-child HIV-1 transmission.
52. β<sub>2</sub>-Adrenergic Receptor Gene Polymorphism Is Associated with Mortality in Septic Shock.
53. Human Cytomegalovirus UL144 Is Associated with Viremia and Infant Development Sequelae in Congenital Infection.
54. Study on the virulence related functions of outer membrane protein T of Escherichia coli K1 pathogenic strain.
55. Genetic Analysis of BPD SP-B Genetic Deficiency Genes in Han Chinese and Study on the Mechanism of Action Leading to BPD.
56. Functional study of GimA, a virulence island gene of Escherichia coli in neonatal meningitis.
57. Functional Promoter Haplotypes of Interleukin-18 Condition Susceptibility to Severe Malarial Anemia and Childhood Mortality.
58. Association of environment and place of birth with asthma in Chinese immigrant children.
59. Procalcitonin versus CRP as an early indicator of fetal infection in preterm premature rupture of membranes.
60. Laboratory aid to the diagnosis and therapy of infection in the neonate.
61. Tumor necrosis factor-α promoter -308 G/A polymorphism and susceptibility to sepsis in very-low-birth-weight infants.
62. A regulatory polymorphism in promoter region of TNFR1 gene is associated with Kawasaki disease in Chinese individuals.
63. Environmental tobacco smoke and male sex modify the influence of IL-13 genetic variants on cord blood IgE levels.
64. Association of glutathione S-transferase Ω 1-1 polymorphisms (A140D and E208K) with the expression of interleukin-8 (IL-8), transforming growth factor beta (TGF-β), and apoptotic protease-activating factor 1 (Apaf-1) in humans chronically exposed to arsenic in drinking water.
65. Toll-Like 4 Receptor Variant, Asp299Gly, and Reduced Risk of Hemorrhagic Cystitis after Hematopoietic Stem Cell Transplantation.
66. Frequencies of functional caspase 12 genotypes in the North Africa population.
67. A functional microsatellite of the <i>macrophage migration inhibitory factor</i> gene associated with meningococcal disease.
68. Immunoregulatory gene polymorphisms in Japanese women with preterm births and periodontitis.
69. Intestinal barrier function in neonatal foals: Options for improvement.
70. Risk of infection and sepsis in severely injured patients related to single nucleotide polymorphisms in the lectin pathway.
71. Tumour necrosis factor gene polymorphism in dengue infection: association with risk of bleeding.
72. Effects of <i>IL</i>-<i>10</i> Haplotype and Atomic Bomb Radiation Exposure on Gastric Cancer Risk.
73. Neonatal infections in Saudi Arabia: association with C-reactive protein, <i>CRP</i>-286 (C&gt;T&gt;A) gene polymorphism and IgG antibodies.
74. Biomarkers for Prediction and Diagnosis of Necrotizing Enterocolitis.
75. Toll-like receptors in Neonatal Sepsis.
76. Air pollution, inflammation and preterm birth in Mexico City: Study design and methods.
77. Short (GT)n Microsatellite Repeats in the Heme Oxygenase-1 Gene Promoter Are Associated with Antioxidant and Anti-Inflammatory Status in Mexican Pediatric Patients with Sepsis.
78. IL-6, IL-10, IL-18, IFN- γ The detection significance of waiting in children with hemophagocytic syndrome.
79. Clinical and basic research on perioperative damage to important organs in children with congenital heart disease.
80. Parto Prematuro - Estudo epidemiológico e genético. <i>O Envolvimento Do Gene HBD1</i>.
81. Gene Polymorphisms and Febrile Neutropenia in Acute Leukemia-No Association with IL-4, CCR-5, IL-1RA, but the MBL-2, ACE, and TLR-4 Are Associated with the Disease in Turkish Patients: A Preliminary Study.
82. Genetic and Environmental Influences on the Prospective Correlation Between Systemic Inflammation and Coronary Heart Disease Death in Male Twins.
83. Association of Polymorphisms in IRAK1, IRAK4 and MyD88, and Severe Invasive Pneumococcal Disease.
84. Are Immune Modulating Single Nucleotide Polymorphisms Associated with Necrotizing Enterocolitis?
85. Clinical relevance of single nucleotide polymorphisms within the 13 cytokine genes in North Indian trauma hemorrhagic shock patients.
86. Polymorphism rs2239185 in <i>vitamin D receptor</i> gene is associated with severe community-acquired pneumonia of children in Chinese Han population: a case-control study.
87. Urinary Proteins, Vitamin D and Genetic Polymorphisms as Risk Factors for Febrile Urinary Tract Infection and Relation with Bacteremia: A Case Control Study.
88. The role of inflammatory response in the occurrence and development of immature brain injury.
89. The significance of pre B cell colony enhancing factor in the diagnosis and treatment of neonatal sepsis.
90. Research progress on heart rate variability in neonatal sepsis.
91. Immune mechanism and clinical study of childhood infection.
92. Interleukin-10-1082 G/A gene polymorphisms in Egyptian children with CAP: A case-control study.
93. Genotyping of vitamin D receptor gene polymorphisms using mismatched amplification mutation assay in neonatal sepsis patients of Odisha, eastern India.
94. Does Haptoglobin Phenotype Influence Postnatal Morbidity in Preterm Neonates?
95. Association of TLR polymorphisms with bronchopulmonary dysplasia.
96. Mannose-binding lectin (MBL) insufficiency protects against the development of systemic inflammatory response after pediatric cardiac surgery.
97. Recent developments in severe sepsis research: from bench to bedside and back.
98. Isolation and identification of Streptococcus agalactiae subsp. Pasteuri, and study on its pathogenicity and mechanism of macrolide resistance.
99. Maternal Interleukin Genotypes Are Associated With NICU Outcomes Among Low-Birth-Weight Infants.
100. Plasma levels of Macrophage Migration inhibitory Factor and D-Dopachrome Tautomerase show a highly specific Profile in early life.
101. Genome-wide association study of sepsis in extremely premature infants.
102. Pathogenicity study of foodborne Cronobacter sakazakii based on suckling mice.
103. Risk of nontyphoidal Salmonella bacteraemia in African children is modified by STAT4.
104. The interleukin-27 -964A>G polymorphism enhances sepsis-induced inflammatory responses and confers susceptibility to the development of sepsis.
105. <i>IL</i>-<i>10RA</i> Mutation as a Risk Factor of Severe Influenza-Associated Encephalopathy: A Case Report.
106. Effect of maternal and neonatal interleukin-6-174 G/C polymorphism on preterm birth and neonatal morbidity.
107. Genetic susceptibility to invasive pneumococcal disease.
108. Candidate gene analysis in pathogenesis of surgically and non-surgically treated necrotizing enterocolitis in preterm infants.
109. Clinical Predictors of Liver Fibrosis in Patients With Chronic Hepatitis B Virus Infection From Children to Adults.
110. Establishment of a humanized mouse model and preliminary exploration of gene therapy research using this model, as well as the clinical significance of interleukin-35 in the diagnosis of early-onset neonatal sepsis.
111. Interleukin-1 β Mechanism study on mediating abnormal differentiation of hippocampal neural stem cells in neonatal rats with sepsis.
112. Establishment of Microdroplet Digital PCR Detection Method for Group B Streptococcus (GBS) and Preliminary Study on Inducing CD4+T Cell Differentiation in Newborns.
113. Research progress in the treatment of alcoholic liver disease based on the hepatointestinal axis.
114. Analysis of Molecular Epidemic Characteristics and Biofilm Formation of Infectious Streptococcus agalactiae.
115. Platelet Glycoprotein VI Haplotypes and the Presentation of Paediatric Sepsis.
116. A prospective investigation of interleukin-8 levels in pediatric acute respiratory failure and acute respiratory distress syndrome.
117. A Toll-like receptor 2 genetic variant modulates occurrence of bacterial infections in patients with sickle cell disease.
118. Acute phase reactant serum amyloid A in inflammation and other diseases.
119. Prevention and treatment of complications related to premature infants.
120. Relationship between IRAK-M gene polymorphism in umbilical cord blood and premature birth and major complications in newborns.
121. Study on the role of novel combination biomarkers in early warning of sepsis in children.
122. Proprotein Convertase Subtilisin/Kexin Type 9 Loss-of-Function Is Detrimental to the Juvenile Host With Septic Shock.
123. Is bronchopulmonary dysplasia decided before birth?
124. Two Faces of Heme Catabolic Pathway in Newborns: A Potential Role of Bilirubin and Carbon Monoxide in Neonatal Inflammatory Diseases.
125. Predicting Severe Enterovirus 71-Infected Hand, Foot, and Mouth Disease: Cytokines and Chemokines.
126. [Association between interleukin-8 rs4073 polymorphisms and susceptibility to neonatal sepsis.]
127. The relationship between the polymorphism of rs4073 locus in 68-IL-8 gene and susceptibility to neonatal sepsis.
128. Comparative genomic study of oral streptococcus and pathogenicity analysis of periodontitis in vivo by Fusobacterium nucleatum.
129. Association between single nucleotide polymorphisms and viral load in congenital cytomegalovirus infection.
130. A case of interleukin-1 receptor associated kinase 4 deficiency and literature review.
131. A Polymorphism of Bactericidal/Permeability-Increasing Protein Affects Its Neutralization Efficiency towards Lipopolysaccharide.
132. Component 1 Inhibitor Missense (Val480Met) Variant Is Associated With Gene Expression and Sepsis Development in Neonatal Lung Disease.
133. Gene-environment interactions related to maternal exposure to environmental and lifestyle-related chemicals during pregnancy and the resulting adverse fetal growth: a review.
134. Pb and Cd exposure linked with Il-10 and Il-13 gene polymorphisms in asthma risk relevant immunomodulation in children.
135. Association between Interleukin-6 rs1800795 Polymorphism and Serum Interleukin-6 Levels and Full-Term Neonatal Sepsis
136. Association between plasminogen activator inhibitor-1 gene polymorphism and susceptibility to neonatal sepsis.
137. Association between interleukin-27 gene polymorphisms and Plasmodium falciparum Malaria.
138. Biomarkers of necrotizing enterocolitis in the era of machine learning and omics.
139. The impact and interaction of TLR2 and IRF-5 gene polymorphisms on the susceptibility to neonatal sepsis.
140. Association of 140-IRF5 gene polymorphism with sICAM1 and 25 (OH) D and neonatal pulmonary infection.
141. The impact and interaction of TLR2 and IRF-5 gene polymorphisms on susceptibility to neonatal sepsis.
142. The correlation between 74-IL-10 gene polymorphism and susceptibility to sepsis in full-term newborns.

**Few articles on gene sequences(3)：**

1. Genetic variants of TNF-[FC12]a, IL-1beta, IL-4 receptor [FC12]a-chain, IL-6 and IL-10 genes are not risk factors for sepsis in low-birth-weight infants.
2. Genetic Polymorphisms and Bacterial Infections in Neonates.
3. Genetic variant rs16944 in IL1B gene is a risk factor for early-onset sepsis susceptibility and outcome in preterm infants.

**IL-6**

**Duplicates(75):**

1. Genetic variants of TNF-[FC12]a, IL-1beta, IL-4 receptor [FC12]a-chain, IL-6 and IL-10 genes are not risk factors for sepsis in low-birth-weight infants.
2. Genetic variants of TNF-α, IL-1β, IL-4 receptor α-chain, IL-6 and IL-10 genes are not risk factors for sepsis in low-birth-weight infants.
3. Association between IL-1ra gene polymorphism and premature delivery.
4. Association between IL-1ra gene polymorphism and premature delivery.
5. Mutations of genes involved in the innate immune system as predictors of sepsis in very low birth weight infants.
6. Cytokine responses and sudden infant death syndrome: genetic, developmental, and environmental risk factors.
7. Cytokine responses and sudden infant death syndrome: genetic, developmental, and environmental risk factors.
8. Genetic polymorphisms and risk for acute renal failure in preterm neonates.
9. Genetic polymorphisms and risk for acute renal failure in preterm neonates.
10. The correlation between systemic inflammatory response syndrome in newborns and IL-10 gene polymorphism.
11. Interleukin-10 and its role in clinical immunoparalysis following pediatric cardiac surgery.
12. Interleukin-10 and its role in clinical immunoparalysis following pediatric cardiac surgery.
13. IL-10, IL-6 and CD14 polymorphisms and sepsis outcome in ventilated very low birth weight infants.
14. Effect of various genetic polymorphisms on the incidence and outcome of severe sepsis.
15. Effect of various genetic polymorphisms on the incidence and outcome of severe sepsis.
16. Genetic Polymorphisms of CD14, toll-like receptor 4, and caspase-recruitment domain 15 are not associated with necrotizing enterocolitis in very low birth weight infants.
17. Genetic basis for necrotizing enterocolitis - Risk factors and their relations to genetic polymorphisms.
18. Genetic basis for necrotizing enterocolitis - Risk factors and their relations to genetic polymorphisms.
19. Molecular biology on the ICU. From understanding to treating sepsis.
20. Genetic association studies in VLBW infants exemplifying susceptibility to sepsis--recent findings and implications for future research.
21. Interleukin-6 gene variants and the risk of sepsis development in children.
22. Interleukin-6 gene variants and the risk of sepsis development in children.
23. Interleukin-6 (-174C) polymorphism and the risk of sepsis in very low birth weight infants: meta-analysis.
24. Interleukin-6 (-174C) polymorphism and the risk of sepsis in very low birth weight infants: meta-analysis.
25. Interleukin-6 polymorphism is associated with chorioamnionitis and neonatal infections in preterm infants.
26. Interleukin-6 polymorphism is associated with chorioamnionitis and neonatal infections in preterm infants.
27. [Genetic markers of predisposition to infectious complications in neonatal infants with respiratory distress syndrome.]
28. Role of polymorphic variants as genetic modulators of infection in neonatal sepsis.
29. Role of polymorphic variants as genetic modulators of infection in neonatal sepsis.
30. An age-related decrease in factor V Leiden frequency among Polish subjects.
31. An age-related decrease in factor V Leiden frequency among Polish subjects.
32. Human Cytomegalovirus UL144 Is Associated with Viremia and Infant Development Sequelae in Congenital Infection.
33. Association between bronchopulmonary dysplasia and MBL2 and IL1-RN polymorphisms.
34. Association between bronchopulmonary dysplasia and MBL2 and IL1-RN polymorphisms.
35. Toll-like 4 receptor variant, Asp299Gly, and reduced risk of hemorrhagic cystitis after hematopoietic stem cell transplantation.
36. Frequencies of functional caspase 12 genotypes in the North Africa population.
37. Genetic association study of tumor necrosis factor-alpha with sepsis and septic shock in Thai pediatric patients.
38. Genetic association study of tumor necrosis factor-alpha with sepsis and septic shock in Thai pediatric patients.
39. Characterization of the acute phase response in critically ill children.
40. Characterization of the acute phase response in critically ill children.
41. Prediction of sepsis-related outcomes in neonates through systematic genotyping of polymorphisms in genes for innate immunity and inflammation: a narrative review and critical perspective.
42. Prediction of sepsis-related outcomes in neonates through systematic genotyping of polymorphisms in genes for innate immunity and inflammation: a narrative review and critical perspective.
43. Neonatal infections in Saudi Arabia: association with C-reactive protein, <i>CRP</i>-286 (C&gt;T&gt;A) gene polymorphism and IgG antibodies.
44. Prognostic markers of pediatric meningococcal sepsis.
45. Prognostic markers of pediatric meningococcal sepsis.
46. Multiple gene-to-gene interactions in children with sepsis: a combination of five gene variants predicts outcome of life-threatening sepsis.
47. Multiple gene-to-gene interactions in children with sepsis: a combination of five gene variants predicts outcome of life-threatening sepsis.
48. Neonatal infections in Saudi Arabia: Association with cytokine gene pollymorphisms.
49. Association of Polymorphisms in IRAK1, IRAK4 and MyD88, and Severe Invasive Pneumococcal Disease.
50. Research progress on heart rate variability in neonatal sepsis.
51. Genome-wide association study of sepsis in extremely premature infants.
52. Systematic Review and Meta-analysis: Gene Association Studies in Neonatal Sepsis.
53. Systematic Review and Meta-analysis: Gene Association Studies in Neonatal Sepsis.
54. Tumor necrosis factor-α -308G/A and -238G/A polymorphisms are associated with increased risks of sepsis: evidence from an updated meta-analysis.
55. Tumor necrosis factor-α -308G/A and -238G/A polymorphisms are associated with increased risks of sepsis: evidence from an updated meta-analysis.
56. Risk of nontyphoidal <i>Salmonella</i> bacteraemia in African children is modified by <i>STAT4</i>.
57. Research progress in the treatment of alcoholic liver disease based on the hepatointestinal axis.
58. The association between interleukin-6 gene -174G/C single nucleotide polymorphism and sepsis: an updated meta-analysis with trial sequential analysis.
59. The association between interleukin-6 gene -174G/C single nucleotide polymorphism and sepsis: an updated meta-analysis with trial sequential analysis.
60. Association of gene polymorphism of bactericidal permeability increasing protein rs4358188, cluster of differentiation 14 rs2569190, interleukin 1β rs1143643 and matrix metalloproteinase-16 rs2664349 with neonatal sepsis.
61. Association between Tumor Necrosis Factor-α Promoter -308 G/A Polymorphism and Early Onset Sepsis in Preterm Infants.
62. Research progress on the relationship between susceptibility to neonatal sepsis and polymorphisms of tumor necrosis factor and interleukin genes.
63. Research progress on the relationship between susceptibility to neonatal sepsis and polymorphisms of tumor necrosis factor and interleukin genes.
64. Proprotein Convertase Subtilisin/Kexin Type 9 Loss-of-Function Is Detrimental to the Juvenile Host With Septic Shock.
65. Genetic variant rs16944 in <i>IL1B</i> gene is a risk factor for early-onset sepsis susceptibility and outcome in preterm infants.
66. [Association between interleukin-8 rs4073 polymorphisms and susceptibility to neonatal sepsis.]
67. The relationship between the polymorphism of rs4073 locus in 68-IL-8 gene and susceptibility to neonatal sepsis.
68. IL-1 β A study on the correlation between gene single nucleotide polymorphism and sepsis in full-term newborns.
69. The relationship between the polymorphism of rs4073 locus in 68-IL-8 gene and susceptibility to neonatal sepsis.
70. The relationship between the polymorphism of rs4073 locus in 68-IL-8 gene and susceptibility to neonatal sepsis.
71. Association of IL-6 -174G > C Polymorphism with Susceptibility to Childhood Sepsis: A Systematic Review and Meta-Analysis.
72. Association between Interleukin-6 rs1800795 Polymorphism and Serum Interleukin-6 Levels and Full-Term Neonatal Sepsis.
73. The impact and interaction of TLR2 and IRF-5 gene polymorphisms on susceptibility to neonatal sepsis.
74. The correlation between 74-IL-10 gene polymorphism and susceptibility to sepsis in full-term newborns.
75. The correlation between 74-IL-10 gene polymorphism and susceptibility to sepsis in full-term newborns.

**Review,systematic analysis, animal experiment（7）：**

1. A study on risk factors associated with PROM and methods for detection of BV flora.
2. Association between lymphotoxin-α intron +252 polymorphism and sepsis: a meta-analysis.
3. Association between IL-6-174G/C Polymorphism and the Risk of Sepsis and Mortality: A Systematic Review and Meta-Analysis.
4. Periodontitis and gestational diabetes mellitus: a systematic review and meta-analysis of observational studies.
5. Prenatal pesticide exposure associated with glycated haemoglobin and markers of metabolic dysfunction in adolescents.
6. Association of Gene Polymorphism of Bactericidal Permeability Increasing Protein Rs4358188, Cluster of Differentiation 14 Rs2569190, Interleukin 1beta Rs1143643 and Matrix Metalloproteinase-16 Rs2664349 with Neonatal Sepsis.
7. Association of IL-6-174G &gt; C Polymorphism with Susceptibility to Childhood Sepsis: A Systematic Review and Meta-Analysis.

**Summarize(4)：**

1. Molecular biology on the ICU - From understanding to treating sepsis.
2. Prediction of sepsis-related outcomes in neonates through systematic genotyping of polymorphisms in genes for innate immunity and inflammation: A narrative review and critical perspective.
3. Elucidating the role of genomics in neonatal sepsis.
4. Research progress on the relationship between susceptibility to neonatal sepsis and polymorphisms of tumor necrosis factor and interleukin genes.

**Studies that include on the title and abstract either having no connection with the subject（137）：**

1. 4G/5G promoter polymorphism in the plasminogen-activator-inhibitor-1 gene and outcome of meningococcal disease. Meningococcal Research Group.
2. Pilot study assessing TNF gene polymorphism as a prognostic marker for disease progression in neonates with sepsis.
3. A family with delayed Bruton syndrome.
4. Severity of meningococcal disease in children and the angiotensin-converting enzyme insertion/deletion polymorphism.
5. Bench-to-bedside review: genetic influences on meningococcal disease.
6. Lower prevalence of IL-4 receptor alpha-chain gene G variant in very-low-birth-weight infants with necrotizing enterocolitis.
7. The role of molecular genetics in the pathogenesis and diagnosis of neonatal sepsis.
8. Polymorphisms of genes involved in innate immunity: association with preterm delivery.
9. Tumor necrosis factor alpha -- 308 polymorphism associated with increased sepsis mortality in ventilated very low birth weight infants.
10. Evaluation of systemic inflammatory responses in neonates with herpes simplex virus infection.
11. Genetic screening for susceptibility to infection in the NICU setting.
12. Virulence and cord blood mononuclear cells cytokine production induced by perinatal listeria monocytogenes strains from different phylogenetic lineages.
13. Association of two tumour necrosis factor gene polymorphisms with the incidence of severe intraventricular haemorrhage in preterm infants.
14. Periodontal therapy reduces the rate of preterm low birth weight in women with pregnancy-associated gingivitis.
15. A macrophage migration inhibitory factor promoter polymorphism is associated with high-density parasitemia in children with malaria.
16. Genetic polymorphisms and infections.
17. Prevalence of two tumor necrosis factor gene polymorphisms in premature infants with early onset sepsis.
18. Genetic Polymorphisms of CD14, toll-like receptor 4, and caspase-recruitment domain 15 are not associated with necrotizing enterocolitis in very low birth weight infants.
19. The correlation between systemic inflammatory response syndrome in newborns and IL-10 gene polymorphism.
20. 4G4G genotype of the plasminogen activator inhibitor-1 promoter polymorphism associates with disseminated intravascular coagulation in children with systemic meningococcemia.
21. Genetic association studies in VLBW infants exemplifying susceptibility to sepsis -: recent findings and implications for future research.
22. Acute inflammation is exacerbated in mice genetically predisposed to a severe protein C deficiency.
23. Investigation of the Role of Pen-Like Receptors in the Development of Immunopathogenic Intestinal Diseases.
24. The extent to which genotype information may add to the prediction of distrurbed perinatal adaption:: none, minor, or major?
25. Sudden infant death syndrome: Review of implicated genetic factors.
26. Basic and clinical research on retinopathy in premature infants.
27. Role of angiotensin-converting enzyme gene polymorphisms in children with sepsis and septic shock.
28. The role of mannose-binding lectin in susceptibility to infection in preterm Neonates.
29. 159C&gt;T CD14 genotype -: Functional effects on innate immune responses in term neonates.
30. An infectious aetiology of sudden infant death syndrome.
31. Susceptibility to meningococcal infection-Nature versus nurture?
32. [Genetic markers of predisposition to infectious complications in neonatal infants with respiratory distress syndrome.]
33. Genetics of susceptibility to malaria related phenotypes.
34. Immaturity, perinatal inflammation, and retinopathy of prematurity: A multi-hit hypothesis.
35. Effects of RANTES and MBL2 gene polymorphisms in sickle cell disease clinical outcomes: association of the g.In1.1T>C RANTES variant with protection against infections.
36. Cytokine gene polymorphisms in preterm infants with necrotising enterocolitis: genetic association study.
37. Lack of association between TLR4 polymorphism and severe gram-negative bacterial infection in neonates.
38. Nitric oxide synthase 2A (NOS2A) polymorphisms are not associated with invasive pneumococcal disease.
39. Interleukin-6 G(-174)C polymorphism is associated with mental retardation in cystic periventricular leucomalacia in preterm infants.
40. Theme Index of Chinese Journal of Contemporary Pediatrics, Volume 12, 2010.
41. Genetic variability in complement activation modulates the systemic inflammatory response syndrome in children.
42. Genetic polymorphisms in the endotoxin receptor may influence platelet count as part of the acute phase response in critically ill children.
43. [Correlations between serum interleukin-18 (IL-18) level, IL-18 gene promoter polymorphisms and the development of sepsis in children.]
44. Common <i>NFKBIL2</i> polymorphisms and susceptibility to pneumococcal disease: a genetic association study.
45. Association of the+874 T/A interferon gamma polymorphism with infections in sickle cell disease.
46. CCR5, RANTES and SDF-1 polymorphisms and mother-to-child HIV-1 transmission.
47. β<sub>2</sub>-Adrenergic Receptor Gene Polymorphism Is Associated with Mortality in Septic Shock.
48. Human Cytomegalovirus UL144 Is Associated with Viremia and Infant Development Sequelae in Congenital Infection.
49. Study on the virulence related functions of outer membrane protein T of Escherichia coli K1 pathogenic strain.
50. Genetic Analysis of BPD SP-B Genetic Deficiency Genes in Han Chinese and Study on the Mechanism of Action Leading to BPD.
51. Functional study of GimA, a virulence island gene of Escherichia coli in neonatal meningitis.
52. Functional Promoter Haplotypes of Interleukin-18 Condition Susceptibility to Severe Malarial Anemia and Childhood Mortality.
53. Association of environment and place of birth with asthma in Chinese immigrant children.
54. Procalcitonin versus CRP as an early indicator of fetal infection in preterm premature rupture of membranes.
55. Laboratory aid to the diagnosis and therapy of infection in the neonate.
56. Tumor necrosis factor-α promoter -308 G/A polymorphism and susceptibility to sepsis in very-low-birth-weight infants.
57. A regulatory polymorphism in promoter region of TNFR1 gene is associated with Kawasaki disease in Chinese individuals.
58. Environmental tobacco smoke and male sex modify the influence of IL-13 genetic variants on cord blood IgE levels.
59. Association of glutathione S-transferase Ω 1-1 polymorphisms (A140D and E208K) with the expression of interleukin-8 (IL-8), transforming growth factor beta (TGF-β), and apoptotic protease-activating factor 1 (Apaf-1) in humans chronically exposed to arsenic in drinking water
60. Toll-Like 4 Receptor Variant, Asp299Gly, and Reduced Risk of Hemorrhagic Cystitis after Hematopoietic Stem Cell Transplantation.
61. Frequencies of functional caspase 12 genotypes in the North Africa population.
62. A functional microsatellite of the <i>macrophage migration inhibitory factor</i> gene associated with meningococcal disease.
63. Immunoregulatory gene polymorphisms in Japanese women with preterm births and periodontitis.
64. Intestinal barrier function in neonatal foals: Options for improvement.
65. Risk of infection and sepsis in severely injured patients related to single nucleotide polymorphisms in the lectin pathway.
66. Tumour necrosis factor gene polymorphism in dengue infection: association with risk of bleeding.
67. Effects of <i>IL</i>-<i>10</i> Haplotype and Atomic Bomb Radiation Exposure on Gastric Cancer Risk.
68. Neonatal infections in Saudi Arabia: association with C-reactive protein, <i>CRP</i>-286 (C&gt;T&gt;A) gene polymorphism and IgG antibodies.
69. Biomarkers for Prediction and Diagnosis of Necrotizing Enterocolitis.
70. Toll-like receptors in Neonatal Sepsis.
71. Air pollution, inflammation and preterm birth in Mexico City: Study design and methods.
72. Short (GT)n Microsatellite Repeats in the Heme Oxygenase-1 Gene Promoter Are Associated with Antioxidant and Anti-Inflammatory Status in Mexican Pediatric Patients with Sepsis.
73. IL-6, IL-10, IL-18, IFN- γ The detection significance of waiting in children with hemophagocytic syndrome.
74. Clinical and basic research on perioperative damage to important organs in children with congenital heart disease.
75. Parto Prematuro - Estudo epidemiológico e genético. <i>O Envolvimento Do Gene HBD1</i>.
76. Genetic Polymorphisms and Sepsis in Premature Neonates.
77. Gene Polymorphisms and Febrile Neutropenia in Acute Leukemia-No Association with IL-4, CCR-5, IL-1RA, but the MBL-2, ACE, and TLR-4 Are Associated with the Disease in Turkish Patients: A Preliminary Study.
78. Genetic and Environmental Influences on the Prospective Correlation Between Systemic Inflammation and Coronary Heart Disease Death in Male Twins.
79. Association of Polymorphisms in IRAK1, IRAK4 and MyD88, and Severe Invasive Pneumococcal Disease.
80. Are Immune Modulating Single Nucleotide Polymorphisms Associated with Necrotizing Enterocolitis?
81. Clinical relevance of single nucleotide polymorphisms within the 13 cytokine genes in North Indian trauma hemorrhagic shock patients.
82. Polymorphism rs2239185 in <i>vitamin D receptor</i> gene is associated with severe community-acquired pneumonia of children in Chinese Han population: a case-control study.
83. Urinary Proteins, Vitamin D and Genetic Polymorphisms as Risk Factors for Febrile Urinary Tract Infection and Relation with Bacteremia: A Case Control Study.
84. The role of inflammatory response in the occurrence and development of immature brain injury.
85. The significance of pre B cell colony enhancing factor in the diagnosis and treatment of neonatal sepsis.
86. Immune mechanism and clinical study of childhood infection.
87. Research progress on heart rate variability in neonatal sepsis.
88. Interleukin-10-1082 G/A gene polymorphisms in Egyptian children with CAP: A case-control study.
89. Genotyping of vitamin D receptor gene polymorphisms using mismatched amplification mutation assay in neonatal sepsis patients of Odisha, eastern India.
90. Does Haptoglobin Phenotype Influence Postnatal Morbidity in Preterm Neonates?
91. Association of TLR polymorphisms with bronchopulmonary dysplasia.
92. Mannose-binding lectin (MBL) insufficiency protects against the development of systemic inflammatory response after pediatric cardiac surgery.
93. Recent developments in severe sepsis research: from bench to bedside and back.
94. Isolation and identification of Streptococcus agalactiae subsp. Pasteuri, and study on its pathogenicity and mechanism of macrolide resistance.
95. Maternal Interleukin Genotypes Are Associated With NICU Outcomes Among Low-Birth-Weight Infants.
96. Plasma levels of Macrophage Migration inhibitory Factor and D-Dopachrome Tautomerase show a highly specific Profile in early life.
97. Genome-wide association study of sepsis in extremely premature infants.
98. Pathogenicity study of foodborne Cronobacter sakazakii based on suckling mice.
99. Risk of nontyphoidal Salmonella bacteraemia in African children is modified by STAT4.
100. The interleukin-27 -964A>G polymorphism enhances sepsis-induced inflammatory responses and confers susceptibility to the development of sepsis.
101. <i>IL</i>-<i>10RA</i> Mutation as a Risk Factor of Severe Influenza-Associated Encephalopathy: A Case Report.
102. Genetic susceptibility to invasive pneumococcal disease.
103. Candidate gene analysis in pathogenesis of surgically and non-surgically treated necrotizing enterocolitis in preterm infants.
104. Clinical Predictors of Liver Fibrosis in Patients With Chronic Hepatitis B Virus Infection From Children to Adults.
105. Establishment of a humanized mouse model and preliminary exploration of gene therapy research using this model, as well as the clinical significance of interleukin-35 in the diagnosis of early-onset neonatal sepsis.
106. Establishment of Microdroplet Digital PCR Detection Method for Group B Streptococcus (GBS) and Preliminary Study on Inducing CD4+T Cell Differentiation in Newborns.
107. Research progress in the treatment of alcoholic liver disease based on the hepatointestinal axis.
108. Analysis of Molecular Epidemic Characteristics and Biofilm Formation of Infectious Streptococcus agalactiae.
109. Platelet Glycoprotein VI Haplotypes and the Presentation of Paediatric Sepsis.
110. A prospective investigation of interleukin-8 levels in pediatric acute respiratory failure and acute respiratory distress syndrome.
111. A Toll-like receptor 2 genetic variant modulates occurrence of bacterial infections in patients with sickle cell disease.
112. Acute phase reactant serum amyloid A in inflammation and other diseases.
113. Prevention and treatment of complications related to premature infants.
114. Relationship between IRAK-M gene polymorphism in umbilical cord blood and premature birth and major complications in newborns.
115. Study on the role of novel combination biomarkers in early warning of sepsis in children.
116. Proprotein Convertase Subtilisin/Kexin Type 9 Loss-of-Function Is Detrimental to the Juvenile Host With Septic Shock.
117. Is bronchopulmonary dysplasia decided before birth?
118. Two Faces of Heme Catabolic Pathway in Newborns: A Potential Role of Bilirubin and Carbon Monoxide in Neonatal Inflammatory Diseases.
119. Genetic variant rs16944 in IL1B gene is a risk factor for early-onset sepsis susceptibility and outcome in preterm infants.
120. Predicting Severe Enterovirus 71-Infected Hand, Foot, and Mouth Disease: Cytokines and Chemokines.
121. [Association between interleukin-8 rs4073 polymorphisms and susceptibility to neonatal sepsis.]
122. IL-1 β A study on the correlation between gene single nucleotide polymorphism and sepsis in full-term newborns.
123. The relationship between the polymorphism of rs4073 locus in 68-IL-8 gene and susceptibility to neonatal sepsis.
124. Comparative genomic study of oral streptococcus and pathogenicity analysis of periodontitis in vivo by Fusobacterium nucleatum.
125. Association between single nucleotide polymorphisms and viral load in congenital cytomegalovirus infection.
126. A case of interleukin-1 receptor associated kinase 4 deficiency and literature review.
127. A Polymorphism of Bactericidal/Permeability-Increasing Protein Affects Its Neutralization Efficiency towards Lipopolysaccharide.
128. Component 1 Inhibitor Missense (Val480Met) Variant Is Associated With Gene Expression and Sepsis Development in Neonatal Lung Disease.
129. Gene-environment interactions related to maternal exposure to environmental and lifestyle-related chemicals during pregnancy and the resulting adverse fetal growth: a review.
130. Pb and Cd exposure linked with Il-10 and Il-13 gene polymorphisms in asthma risk relevant immunomodulation in children.
131. Association between plasminogen activator inhibitor-1 gene polymorphism and susceptibility to neonatal sepsis.
132. Association between interleukin-27 gene polymorphisms and Plasmodium falciparum Malaria
133. Biomarkers of necrotizing enterocolitis in the era of machine learning and omics.
134. The impact and interaction of TLR2 and IRF-5 gene polymorphisms on the susceptibility to neonatal sepsis.
135. Association of 140-IRF5 gene polymorphism with sICAM1 and 25 (OH) D and neonatal pulmonary infection.
136. The impact and interaction of TLR2 and IRF-5 gene polymorphisms on susceptibility to neonatal sepsis.
137. The correlation between 74-IL-10 gene polymorphism and susceptibility to sepsis in full-term newborns.

**Few articles on gene sequences(1)：**

1. Polymorphisms of IL-6 and IL-10 genes in umbilical cord blood and their association with serum levels and premature infant infection.

**Incomplete or no research data（2）：**

1. Is interleukin-6 -174 genotype associated with the development of septicemia in preterm infants?
2. Association between Interleukin-6 rs1800795 Polymorphism and Serum Interleukin-6 Levels and Full-Term Neonatal Sepsis.

**IL-8**

**Duplicates(76):**

1. Genetic variants of TNF-[FC12]a, IL-1beta, IL-4 receptor [FC12]a-chain, IL-6 and IL-10 genes are not risk factors for sepsis in low-birth-weight infants.
2. Genetic variants of TNF-[FC12]a, IL-1beta, IL-4 receptor [FC12]a-chain, IL-6 and IL-10 genes are not risk factors for sepsis in low-birth-weight infants.
3. Association between IL-1ra gene polymorphism and premature delivery.
4. Association between IL-1ra gene polymorphism and premature delivery.
5. Cytokine responses and sudden infant death syndrome: genetic, developmental, and environmental risk factors.
6. Cytokine responses and sudden infant death syndrome: genetic, developmental, and environmental risk factors.
7. Genetic polymorphisms and risk for acute renal failure in preterm neonates.
8. Genetic polymorphisms and risk for acute renal failure in preterm neonates
9. Interleukin-10 and its role in clinical immunoparalysis following pediatric cardiac surgery.
10. Interleukin-10 and its role in clinical immunoparalysis following pediatric cardiac surgery.
11. IL-10, IL-6 and CD14 polymorphisms and sepsis outcome in ventilated very low birth weight infants.
12. IL-10, IL-6 and CD14 polymorphisms and sepsis outcome in ventilated very low birth weight infants.
13. Effect of various genetic polymorphisms on the incidence and outcome of severe sepsis.
14. Effect of various genetic polymorphisms on the incidence and outcome of severe sepsis.
15. Genetic Polymorphisms of CD14, toll-like receptor 4, and caspase-recruitment domain 15 are not associated with necrotizing enterocolitis in very low birth weight infants.
16. Genetic basis for necrotizing enterocolitis - Risk factors and their relations to genetic polymorphisms.
17. Genetic basis for necrotizing enterocolitis - Risk factors and their relations to genetic polymorphisms.
18. The correlation between systemic inflammatory response syndrome in newborns and IL-10 gene polymorphism.
19. Genetic association studies in VLBW infants exemplifying susceptibility to sepsis--recent findings and implications for future research.
20. Interleukin-6 gene variants and the risk of sepsis development in children.
21. Interleukin-6 gene variants and the risk of sepsis development in children.
22. Interleukin-6 (-174C) polymorphism and the risk of sepsis in very low birth weight infants: meta-analysis.
23. Interleukin-6 (-174C) polymorphism and the risk of sepsis in very low birth weight infants: meta-analysis.
24. Interleukin-6 polymorphism is associated with chorioamnionitis and neonatal infections in preterm infants.
25. Interleukin-6 polymorphism is associated with chorioamnionitis and neonatal infections in preterm infants.
26. [Genetic markers of predisposition to infectious complications in neonatal infants with respiratory distress syndrome.]
27. Role of polymorphic variants as genetic modulators of infection in neonatal sepsis.
28. Role of polymorphic variants as genetic modulators of infection in neonatal sepsis.
29. An age-related decrease in factor V Leiden frequency among Polish subjects.
30. An age-related decrease in factor V Leiden frequency among Polish subjects.
31. Human cytomegalovirus UL144 is associated with viremia and infant development sequelae in congenital infection.
32. Association between bronchopulmonary dysplasia and MBL2 and IL1-RN polymorphisms.
33. Association between bronchopulmonary dysplasia and MBL2 and IL1-RN polymorphisms.
34. Toll-Like 4 Receptor Variant, Asp299Gly, and Reduced Risk of Hemorrhagic Cystitis after Hematopoietic Stem Cell Transplantation.
35. Frequencies of functional caspase 12 genotypes in the North Africa population.
36. Genetic association study of tumor necrosis factor-alpha with sepsis and septic shock in Thai pediatric patients.
37. Genetic association study of tumor necrosis factor-alpha with sepsis and septic shock in Thai pediatric patients.
38. Characterization of the acute phase response in critically ill children.
39. Characterization of the acute phase response in critically ill children.
40. Prediction of sepsis-related outcomes in neonates through systematic genotyping of polymorphisms in genes for innate immunity and inflammation: a narrative review and critical perspective.
41. Prediction of sepsis-related outcomes in neonates through systematic genotyping of polymorphisms in genes for innate immunity and inflammation: a narrative review and critical perspective.
42. Neonatal infections in Saudi Arabia: association with C-reactive protein, CRP -286 (C>T>A) gene polymorphism and IgG antibodies.
43. Prognostic markers of pediatric meningococcal sepsis.
44. Prognostic markers of pediatric meningococcal sepsis.
45. Multiple gene-to-gene interactions in children with sepsis: a combination of five gene variants predicts outcome of life-threatening sepsis.
46. Multiple gene-to-gene interactions in children with sepsis: a combination of five gene variants predicts outcome of life-threatening sepsis.
47. Neonatal infections in Saudi Arabia: Association with cytokine gene pollymorphisms.
48. Association of Polymorphisms in IRAK1, IRAK4 and MyD88, and Severe Invasive Pneumococcal Disease.
49. Research progress on heart rate variability in neonatal sepsis.
50. Genome-wide association study of sepsis in extremely premature infants.
51. Systematic Review and Meta-analysis: Gene Association Studies in Neonatal Sepsis.
52. Systematic Review and Meta-analysis: Gene Association Studies in Neonatal Sepsis.
53. Tumor necrosis factor-α -308G/A and -238G/A polymorphisms are associated with increased risks of sepsis: evidence from an updated meta-analysis.
54. Tumor necrosis factor-α -308G/A and -238G/A polymorphisms are associated with increased risks of sepsis: evidence from an updated meta-analysis.
55. Risk of nontyphoidal <i>Salmonella</i> bacteraemia in African children is modified by <i>STAT4</i>.
56. Research progress in the treatment of alcoholic liver disease based on the hepatointestinal axis.
57. The association between interleukin-6 gene -174G/C single nucleotide polymorphism and sepsis: an updated meta-analysis with trial sequential analysis.
58. The association between interleukin-6 gene -174G/C single nucleotide polymorphism and sepsis: an updated meta-analysis with trial sequential analysis.
59. Association of gene polymorphism of bactericidal permeability increasing protein rs4358188, cluster of differentiation 14 rs2569190, interleukin 1β rs1143643 and matrix metalloproteinase-16 rs2664349 with neonatal sepsis.
60. Association between Tumor Necrosis Factor-α Promoter -308 G/A Polymorphism and Early Onset Sepsis in Preterm Infants.
61. Association between Tumor Necrosis Factor-α Promoter -308 G/A Polymorphism and Early Onset Sepsis in Preterm Infants.
62. Research progress on the relationship between susceptibility to neonatal sepsis and polymorphisms of tumor necrosis factor and interleukin genes.
63. Research progress on the relationship between susceptibility to neonatal sepsis and polymorphisms of tumor necrosis factor and interleukin genes.
64. Proprotein Convertase Subtilisin/Kexin Type 9 Loss-of-Function Is Detrimental to the Juvenile Host With Septic Shock.
65. Genetic variant rs16944 in <i>IL1B</i> gene is a risk factor for early-onset sepsis susceptibility and outcome in preterm infants.
66. [Association between interleukin-8 rs4073 polymorphisms and susceptibility to neonatal sepsis.]
67. [Association between interleukin-8 rs4073 polymorphisms and susceptibility to neonatal sepsis.]
68. The relationship between the rs4073 polymorphism of IL-8 gene and susceptibility to neonatal sepsis.
69. IL-1 β A study on the correlation between gene single nucleotide polymorphism and sepsis in full-term newborns.
70. The relationship between the rs4073 polymorphism of IL-8 gene and susceptibility to neonatal sepsis.
71. The relationship between the rs4073 polymorphism of IL-8 gene and susceptibility to neonatal sepsis.
72. Association of IL-6 -174G > C Polymorphism with Susceptibility to Childhood Sepsis: A Systematic Review and Meta-Analysis.
73. Association between Interleukin-6 rs1800795 Polymorphism and Serum Interleukin-6 Levels and Full-Term Neonatal Sepsis.
74. The impact and interaction of TLR2 and IRF-5 gene polymorphisms on susceptibility to neonatal sepsis.
75. The correlation between 74-IL-10 gene polymorphism and susceptibility to sepsis in full-term newborns.
76. The correlation between 74-IL-10 gene polymorphism and susceptibility to sepsis in full-term newborns.

**Review,systematic analysis, animal experiment（6）：**

1. Association between lymphotoxin-α intron +252 polymorphism and sepsis: a meta-analysis.
2. Association between IL-6-174G/C Polymorphism and the Risk of Sepsis and Mortality: A Systematic Review and Meta-Analysis.
3. Periodontitis and gestational diabetes mellitus: a systematic review and meta-analysis of observational studies.
4. Prenatal pesticide exposure associated with glycated haemoglobin and markers of metabolic dysfunction in adolescents.
5. Association of Gene Polymorphism of Bactericidal Permeability Increasing Protein Rs4358188, Cluster of Differentiation 14 Rs2569190, Interleukin 1beta Rs1143643 and Matrix Metalloproteinase-16 Rs2664349 with Neonatal Sepsis.
6. Association of IL-6-174G &gt; C Polymorphism with Susceptibility to Childhood Sepsis: A Systematic Review and Meta-Analysis.

**Summarize(4)：**

1. Molecular biology on the ICU - From understanding to treating sepsis.
2. Prediction of sepsis-related outcomes in neonates through systematic genotyping of polymorphisms in genes for innate immunity and inflammation: A narrative review and critical perspective.
3. Elucidating the role of genomics in neonatal sepsis.
4. Research progress on the relationship between susceptibility to neonatal sepsis and polymorphisms of tumor necrosis factor and interleukin genes.

**Studies that include on the title and abstract either having no connection with the subject（144）：**

1. 4G/5G promoter polymorphism in the plasminogen-activator-inhibitor-1 gene and outcome of meningococcal disease. Meningococcal Research Group.
2. Pilot study assessing TNF gene polymorphism as a prognostic marker for disease progression in neonates with sepsis.
3. A family with delayed Bruton syndrome.
4. Severity of meningococcal disease in children and the angiotensin-converting enzyme insertion/deletion polymorphism.
5. Bench-to-bedside review: genetic influences on meningococcal disease.
6. Is interleukin-6 -174 genotype associated with the development of septicemia in preterm infants?
7. Lower prevalence of IL-4 receptor alpha-chain gene G variant in very-low-birth-weight infants with necrotizing enterocolitis.
8. Genetic variants of TNF-α, IL-1β, IL-4 receptor α-chain, IL-6 and IL-10 genes are not risk factors for sepsis in low-birth-weight infants.
9. Mutations of genes involved in the innate immune system as predictors of sepsis in very low birth weight infants.
10. The role of molecular genetics in the pathogenesis and diagnosis of neonatal sepsis.
11. Polymorphisms of genes involved in innate immunity: association with preterm delivery.
12. Tumor necrosis factor alpha -- 308 polymorphism associated with increased sepsis mortality in ventilated very low birth weight infants.
13. Evaluation of systemic inflammatory responses in neonates with herpes simplex virus infection.
14. Genetic screening for susceptibility to infection in the NICU setting.
15. Virulence and cord blood mononuclear cells cytokine production induced by perinatal listeria monocytogenes strains from different phylogenetic lineages.
16. Association of two tumour necrosis factor gene polymorphisms with the incidence of severe intraventricular haemorrhage in preterm infants.
17. Periodontal therapy reduces the rate of preterm low birth weight in women with pregnancy-associated gingivitis.
18. The correlation between systemic inflammatory response syndrome in newborns and IL-10 gene polymorphism.
19. A macrophage migration inhibitory factor promoter polymorphism is associated with high-density parasitemia in children with malaria.
20. Interleukin-6-174-genotype, sepsis and cerebral injury in very low birth weight infants.
21. Genetic polymorphisms and infections.
22. Prevalence of two tumor necrosis factor gene polymorphisms in premature infants with early onset sepsis.
23. Genetic Polymorphisms of CD14, toll-like receptor 4, and caspase-recruitment domain 15 are not associated with necrotizing enterocolitis in very low birth weight infants.
24. Molecular biology on the ICU. From understanding to treating sepsis.
25. 4G4G genotype of the plasminogen activator inhibitor-1 promoter polymorphism associates with disseminated intravascular coagulation in children with systemic meningococcemia.
26. Genetic association studies in VLBW infants exemplifying susceptibility to sepsis -: recent findings and implications for future research.
27. Acute inflammation is exacerbated in mice genetically predisposed to a severe protein C deficiency.
28. Investigation of the Role of Pen-Like Receptors in the Development of Immunopathogenic Intestinal Diseases.
29. The extent to which genotype information may add to the prediction of distrurbed perinatal adaption:: none, minor, or major?
30. Sudden infant death syndrome: Review of implicated genetic factors.
31. Basic and clinical research on retinopathy in premature infants.
32. Role of angiotensin-converting enzyme gene polymorphisms in children with sepsis and septic shock.
33. The role of mannose-binding lectin in susceptibility to infection in preterm Neonates.
34. A study on risk factors associated with PROM and methods for detection of BV flora.
35. 159C&gt;T CD14 genotype -: Functional effects on innate immune responses in term neonates.
36. An infectious aetiology of sudden infant death syndrome.
37. Susceptibility to meningococcal infection-Nature versus nurture?
38. Genetic polymorphisms of IL-6-174 and IL-10-1082 in full term neonates with late onset blood stream infections.
39. [Genetic markers of predisposition to infectious complications in neonatal infants with respiratory distress syndrome.]
40. Genetics of susceptibility to malaria related phenotypes.
41. Immaturity, perinatal inflammation, and retinopathy of prematurity: A multi-hit hypothesis.
42. Effects of RANTES and MBL2 gene polymorphisms in sickle cell disease clinical outcomes: association of the g.In1.1T>C RANTES variant with protection against infections.
43. Cytokine gene polymorphisms in preterm infants with necrotising enterocolitis: genetic association study.
44. Lack of association between TLR4 polymorphism and severe gram-negative bacterial infection in neonates.
45. Nitric oxide synthase 2A (NOS2A) polymorphisms are not associated with invasive pneumococcal disease.
46. Interleukin-6 G(-174)C polymorphism is associated with mental retardation in cystic periventricular leucomalacia in preterm infants.
47. Theme Index of Chinese Journal of Contemporary Pediatrics, Volume 12, 2010.
48. Genetic variability in complement activation modulates the systemic inflammatory response syndrome in children.
49. Genetic polymorphisms in the endotoxin receptor may influence platelet count as part of the acute phase response in critically ill children.
50. [Correlations between serum interleukin-18 (IL-18) level, IL-18 gene promoter polymorphisms and the development of sepsis in children.]
51. Common <i>NFKBIL2</i> polymorphisms and susceptibility to pneumococcal disease: a genetic association study.
52. Association of the+874 T/A interferon gamma polymorphism with infections in sickle cell disease.
53. CCR5, RANTES and SDF-1 polymorphisms and mother-to-child HIV-1 transmission.
54. β<sub>2</sub>-Adrenergic Receptor Gene Polymorphism Is Associated with Mortality in Septic Shock.
55. Human Cytomegalovirus UL144 Is Associated with Viremia and Infant Development Sequelae in Congenital Infection.
56. Study on the virulence related functions of outer membrane protein T of Escherichia coli K1 pathogenic strain.
57. Genetic Analysis of BPD SP-B Genetic Deficiency Genes in Han Chinese and Study on the Mechanism of Action Leading to BPD.
58. Functional study of GimA, a virulence island gene of Escherichia coli in neonatal meningitis.
59. Functional Promoter Haplotypes of Interleukin-18 Condition Susceptibility to Severe Malarial Anemia and Childhood Mortality.
60. Association of environment and place of birth with asthma in Chinese immigrant children.
61. Procalcitonin versus CRP as an early indicator of fetal infection in preterm premature rupture of membranes.
62. Laboratory aid to the diagnosis and therapy of infection in the neonate.
63. Tumor necrosis factor-α promoter -308 G/A polymorphism and susceptibility to sepsis in very-low-birth-weight infants.
64. A regulatory polymorphism in promoter region of TNFR1 gene is associated with Kawasaki disease in Chinese individuals.
65. Environmental tobacco smoke and male sex modify the influence of IL-13 genetic variants on cord blood IgE levels.
66. Association of glutathione S-transferase Ω 1-1 polymorphisms (A140D and E208K) with the expression of interleukin-8 (IL-8), transforming growth factor beta (TGF-β), and apoptotic protease-activating factor 1 (Apaf-1) in humans chronically exposed to arsenic in drinking water.
67. Toll-Like 4 Receptor Variant, Asp299Gly, and Reduced Risk of Hemorrhagic Cystitis after Hematopoietic Stem Cell Transplantation.
68. Frequencies of functional caspase 12 genotypes in the North Africa population.
69. A functional microsatellite of the <i>macrophage migration inhibitory factor</i> gene associated with meningococcal disease.
70. Immunoregulatory gene polymorphisms in Japanese women with preterm births and periodontitis
71. Intestinal barrier function in neonatal foals: Options for improvement.
72. Risk of infection and sepsis in severely injured patients related to single nucleotide polymorphisms in the lectin pathway.
73. Tumour necrosis factor gene polymorphism in dengue infection: association with risk of bleeding.
74. Effects of <i>IL</i>-<i>10</i> Haplotype and Atomic Bomb Radiation Exposure on Gastric Cancer Risk.
75. Neonatal infections in Saudi Arabia: association with C-reactive protein, <i>CRP</i>-286 (C&gt;T&gt;A) gene polymorphism and IgG antibodies.
76. Biomarkers for Prediction and Diagnosis of Necrotizing Enterocolitis.
77. Toll-like receptors in Neonatal Sepsis.
78. Air pollution, inflammation and preterm birth in Mexico City: Study design and methods.
79. Short (GT)n Microsatellite Repeats in the Heme Oxygenase-1 Gene Promoter Are Associated with Antioxidant and Anti-Inflammatory Status in Mexican Pediatric Patients with Sepsis.
80. IL-6, IL-10, IL-18, IFN- γ The detection significance of waiting in children with hemophagocytic syndrome.
81. Parto Prematuro - Estudo epidemiológico e genético. <i>O Envolvimento Do Gene HBD1</i>.
82. Gene Polymorphisms and Febrile Neutropenia in Acute Leukemia-No Association with IL-4, CCR-5, IL-1RA, but the MBL-2, ACE, and TLR-4 Are Associated with the Disease in Turkish Patients: A Preliminary Study.
83. Genetic and Environmental Influences on the Prospective Correlation Between Systemic Inflammation and Coronary Heart Disease Death in Male Twins.
84. Neonatal infections in Saudi Arabia: Association with cytokine gene polymorphisms.
85. Association of Polymorphisms in IRAK1, IRAK4 and MyD88, and Severe Invasive Pneumococcal Disease.
86. Are Immune Modulating Single Nucleotide Polymorphisms Associated with Necrotizing Enterocolitis?
87. Clinical relevance of single nucleotide polymorphisms within the 13 cytokine genes in North Indian trauma hemorrhagic shock patients.
88. Polymorphism rs2239185 in <i>vitamin D receptor</i> gene is associated with severe community-acquired pneumonia of children in Chinese Han population: a case-control study.
89. Urinary Proteins, Vitamin D and Genetic Polymorphisms as Risk Factors for Febrile Urinary Tract Infection and Relation with Bacteremia: A Case Control Study.
90. The role of inflammatory response in the occurrence and development of immature brain injury.
91. The significance of pre B cell colony enhancing factor in the diagnosis and treatment of neonatal sepsis.
92. Research progress on heart rate variability in neonatal sepsis.
93. Immune mechanism and clinical study of childhood infection.
94. Interleukin-10-1082 G/A gene polymorphisms in Egyptian children with CAP: A case-control study.
95. Genotyping of vitamin D receptor gene polymorphisms using mismatched amplification mutation assay in neonatal sepsis patients of Odisha, eastern India.
96. Does Haptoglobin Phenotype Influence Postnatal Morbidity in Preterm Neonates?
97. Association of TLR polymorphisms with bronchopulmonary dysplasia.
98. Mannose-binding lectin (MBL) insufficiency protects against the development of systemic inflammatory response after pediatric cardiac surgery.
99. Recent developments in severe sepsis research: from bench to bedside and back.
100. Isolation and identification of Streptococcus agalactiae subsp. Pasteuri, and study on its pathogenicity and mechanism of macrolide resistance.
101. Maternal Interleukin Genotypes Are Associated With NICU Outcomes Among Low-Birth-Weight Infants.
102. Genetic Polymorphisms and Bacterial Infections in Neonates.
103. Plasma levels of Macrophage Migration inhibitory Factor and D-Dopachrome Tautomerase show a highly specific Profile in early life.
104. Genome-wide association study of sepsis in extremely premature infants.
105. Pathogenicity study of foodborne Cronobacter sakazakii based on suckling mice.
106. Risk of nontyphoidal Salmonella bacteraemia in African children is modified by STAT4.
107. The interleukin-27 -964A>G polymorphism enhances sepsis-induced inflammatory responses and confers susceptibility to the development of sepsis.
108. <i>IL</i>-<i>10RA</i> Mutation as a Risk Factor of Severe Influenza-Associated Encephalopathy: A Case Report.
109. Effect of maternal and neonatal interleukin-6-174 G/C polymorphism on preterm birth and neonatal morbidity.
110. Genetic susceptibility to invasive pneumococcal disease.
111. Candidate gene analysis in pathogenesis of surgically and non-surgically treated necrotizing enterocolitis in preterm infants.
112. Clinical Predictors of Liver Fibrosis in Patients With Chronic Hepatitis B Virus Infection From Children to Adults.
113. Establishment of a humanized mouse model and preliminary exploration of gene therapy research using this model, as well as the clinical significance of interleukin-35 in the diagnosis of early-onset neonatal sepsis.
114. Establishment of Microdroplet Digital PCR Detection Method for Group B Streptococcus (GBS) and Preliminary Study on Inducing CD4+T Cell Differentiation in Newborns.
115. Research progress in the treatment of alcoholic liver disease based on the hepatointestinal axis.
116. Analysis of Molecular Epidemic Characteristics and Biofilm Formation of Infectious Streptococcus agalactiae.
117. Platelet Glycoprotein VI Haplotypes and the Presentation of Paediatric Sepsis.
118. A prospective investigation of interleukin-8 levels in pediatric acute respiratory failure and acute respiratory distress syndrome.
119. A Toll-like receptor 2 genetic variant modulates occurrence of bacterial infections in patients with sickle cell disease.
120. Acute phase reactant serum amyloid A in inflammation and other diseases.
121. Prevention and treatment of complications related to premature infants.
122. Relationship between IRAK-M gene polymorphism in umbilical cord blood and premature birth and major complications in newborns.
123. Study on the role of novel combination biomarkers in early warning of sepsis in children.
124. Proprotein Convertase Subtilisin/Kexin Type 9 Loss-of-Function Is Detrimental to the Juvenile Host With Septic Shock.
125. Is bronchopulmonary dysplasia decided before birth?
126. Two Faces of Heme Catabolic Pathway in Newborns: A Potential Role of Bilirubin and Carbon Monoxide in Neonatal Inflammatory Diseases.
127. Genetic variant rs16944 in IL1B gene is a risk factor for early-onset sepsis susceptibility and outcome in preterm infants.
128. Predicting Severe Enterovirus 71-Infected Hand, Foot, and Mouth Disease: Cytokines and Chemokines.
129. IIL-1 β A study on the correlation between gene single nucleotide polymorphism and sepsis in full-term newborns.
130. Comparative genomic study of oral streptococcus and pathogenicity analysis of periodontitis in vivo by Fusobacterium nucleatum.
131. Association between single nucleotide polymorphisms and viral load in congenital cytomegalovirus infection.
132. A case of interleukin-1 receptor associated kinase 4 deficiency and literature review.
133. A Polymorphism of Bactericidal/Permeability-Increasing Protein Affects Its Neutralization Efficiency towards Lipopolysaccharide.
134. Component 1 Inhibitor Missense (Val480Met) Variant Is Associated With Gene Expression and Sepsis Development in Neonatal Lung Disease.
135. Gene-environment interactions related to maternal exposure to environmental and lifestyle-related chemicals during pregnancy and the resulting adverse fetal growth: a review
136. Pb and Cd exposure linked with Il-10 and Il-13 gene polymorphisms in asthma risk relevant immunomodulation in children.
137. Association between Interleukin-6 rs1800795 Polymorphism and Serum Interleukin-6 Levels and Full-Term Neonatal Sepsis.
138. Association between plasminogen activator inhibitor-1 gene polymorphism and susceptibility to neonatal sepsis.
139. Association between interleukin-27 gene polymorphisms and Plasmodium falciparum Malaria.
140. Biomarkers of necrotizing enterocolitis in the era of machine learning and omics.
141. The impact and interaction of TLR2 and IRF-5 gene polymorphisms on the susceptibility to neonatal sepsis.
142. Association of 140-IRF5 gene polymorphism with sICAM1 and 25 (OH) D and neonatal pulmonary infection.
143. The impact and interaction of TLR2 and IRF-5 gene polymorphisms on susceptibility to neonatal sepsis.
144. The correlation between 74-IL-10 gene polymorphism and susceptibility to sepsis in full-term newborns.

**IL-10**

**Duplicates(75):**

1. Genetic variants of TNF-[FC12]a, IL-1beta, IL-4 receptor [FC12]a-chain, IL-6 and IL-10 genes are not risk factors for sepsis in low-birth-weight infants.
2. Genetic variants of TNF-[FC12]a, IL-1beta, IL-4 receptor [FC12]a-chain, IL-6 and IL-10 genes are not risk factors for sepsis in low-birth-weight infants.
3. Association between IL-1ra gene polymorphism and premature delivery.
4. Association between IL-1ra gene polymorphism and premature delivery.
5. Cytokine responses and sudden infant death syndrome: genetic, developmental, and environmental risk factors.
6. Cytokine responses and sudden infant death syndrome: genetic, developmental, and environmental risk factors.
7. Genetic polymorphisms and risk for acute renal failure in preterm neonates.
8. Genetic polymorphisms and risk for acute renal failure in preterm neonates.
9. The correlation between systemic inflammatory response syndrome in newborns and IL-10 gene polymorphism.
10. Interleukin-10 and its role in clinical immunoparalysis following pediatric cardiac surgery.
11. Interleukin-10 and its role in clinical immunoparalysis following pediatric cardiac surgery.
12. IL-10, IL-6 and CD14 polymorphisms and sepsis outcome in ventilated very low birth weight infants.
13. Effect of various genetic polymorphisms on the incidence and outcome of severe sepsis.
14. Effect of various genetic polymorphisms on the incidence and outcome of severe sepsis.
15. Genetic polymorphisms of CD14, toll-like receptor 4, and caspase-recruitment domain 15 are not associated with necrotizing enterocolitis in very low birth weight infants.
16. Genetic basis for necrotizing enterocolitis - Risk factors and their relations to genetic polymorphisms.
17. Genetic basis for necrotizing enterocolitis - Risk factors and their relations to genetic polymorphisms.
18. Molecular biology on the ICU. From understanding to treating sepsis.
19. Genetic association studies in VLBW infants exemplifying susceptibility to sepsis -: recent findings and implications for future research.
20. Interleukin-6 gene variants and the risk of sepsis development in children.
21. Interleukin-6 gene variants and the risk of sepsis development in children.
22. Interleukin-6 (-174C) polymorphism and the risk of sepsis in very low birth weight infants: meta-analysis.
23. Interleukin-6 (-174C) polymorphism and the risk of sepsis in very low birth weight infants: meta-analysis.
24. Interleukin-6 polymorphism is associated with chorioamnionitis and neonatal infections in preterm infants.
25. Interleukin-6 polymorphism is associated with chorioamnionitis and neonatal infections in preterm infants.
26. [Genetic markers of predisposition to infectious complications in neonatal infants with respiratory distress syndrome.]
27. Role of polymorphic variants as genetic modulators of infection in neonatal sepsis.
28. Role of polymorphic variants as genetic modulators of infection in neonatal sepsis.
29. An age-related decrease in factor V Leiden frequency among Polish subjects.
30. An age-related decrease in factor V Leiden frequency among Polish subjects.
31. Human cytomegalovirus UL144 is associated with viremia and infant development sequelae in congenital infection.
32. Association between bronchopulmonary dysplasia and MBL2 and IL1-RN polymorphisms.
33. Association between bronchopulmonary dysplasia and MBL2 and IL1-RN polymorphisms.
34. Toll-Like 4 Receptor Variant, Asp299Gly, and Reduced Risk of Hemorrhagic Cystitis after Hematopoietic Stem Cell Transplantation.
35. Frequencies of functional caspase 12 genotypes in the North Africa population.
36. Genetic association study of tumor necrosis factor-alpha with sepsis and septic shock in Thai pediatric patients.
37. Genetic association study of tumor necrosis factor-alpha with sepsis and septic shock in Thai pediatric patients.
38. Characterization of the acute phase response in critically ill children.
39. Characterization of the acute phase response in critically ill children.
40. Prediction of sepsis-related outcomes in neonates through systematic genotyping of polymorphisms in genes for innate immunity and inflammation: a narrative review and critical perspective.
41. Prediction of sepsis-related outcomes in neonates through systematic genotyping of polymorphisms in genes for innate immunity and inflammation: a narrative review and critical perspective.
42. Neonatal infections in Saudi Arabia: association with C-reactive protein, CRP -286 (C>T>A) gene polymorphism and IgG antibodies.
43. Prognostic markers of pediatric meningococcal sepsis.
44. Prognostic markers of pediatric meningococcal sepsis.
45. Multiple gene-to-gene interactions in children with sepsis: a combination of five gene variants predicts outcome of life-threatening sepsis.
46. Multiple gene-to-gene interactions in children with sepsis: a combination of five gene variants predicts outcome of life-threatening sepsis.
47. Neonatal infections in Saudi Arabia: Association with cytokine gene pollymorphisms.
48. Association of Polymorphisms in IRAK1, IRAK4 and MyD88, and Severe Invasive Pneumococcal Disease.
49. Research progress on heart rate variability in neonatal sepsis.
50. Genome-wide association study of sepsis in extremely premature infants.
51. Systematic Review and Meta-analysis: Gene Association Studies in Neonatal Sepsis.
52. Systematic Review and Meta-analysis: Gene Association Studies in Neonatal Sepsis.
53. Tumor necrosis factor-α -308G/A and -238G/A polymorphisms are associated with increased risks of sepsis: evidence from an updated meta-analysis.
54. Tumor necrosis factor-α -308G/A and -238G/A polymorphisms are associated with increased risks of sepsis: evidence from an updated meta-analysis.
55. Risk of nontyphoidal <i>Salmonella</i> bacteraemia in African children is modified by <i>STAT4</i>.
56. Research progress in the treatment of alcoholic liver disease based on the hepatointestinal axis.
57. The association between interleukin-6 gene -174G/C single nucleotide polymorphism and sepsis: an updated meta-analysis with trial sequential analysis.
58. The association between interleukin-6 gene -174G/C single nucleotide polymorphism and sepsis: an updated meta-analysis with trial sequential analysis.
59. Association of Gene Polymorphism of Bactericidal Permeability Increasing Protein Rs4358188, Cluster of Differentiation 14 Rs2569190, Interleukin 1beta Rs1143643 and Matrix Metalloproteinase-16 Rs2664349 with Neonatal Sepsis.
60. Association between Tumor Necrosis Factor-α Promoter -308 G/A Polymorphism and Early Onset Sepsis in Preterm Infants.
61. Association between Tumor Necrosis Factor-α Promoter -308 G/A Polymorphism and Early Onset Sepsis in Preterm Infants.
62. Research progress on the relationship between susceptibility to neonatal sepsis and polymorphisms of tumor necrosis factor and interleukin genes.
63. Research progress on the relationship between susceptibility to neonatal sepsis and polymorphisms of tumor necrosis factor and interleukin genes.
64. Proprotein Convertase Subtilisin/Kexin Type 9 Loss-of-Function Is Detrimental to the Juvenile Host With Septic Shock.
65. Genetic variant rs16944 in <i>IL1B</i> gene is a risk factor for early-onset sepsis susceptibility and outcome in preterm infants.
66. [Association between interleukin-8 rs4073 polymorphisms and susceptibility to neonatal sepsis.]
67. The relationship between the rs4073 polymorphism of IL-8 gene and susceptibility to neonatal sepsis.
68. IL-1 β A study on the correlation between gene single nucleotide polymorphism and sepsis in full-term newborns.
69. The relationship between the rs4073 polymorphism of IL-8 gene and susceptibility to neonatal sepsis.
70. The relationship between the rs4073 polymorphism of IL-8 gene and susceptibility to neonatal sepsis.
71. Association of IL-6 -174G > C Polymorphism with Susceptibility to Childhood Sepsis: A Systematic Review and Meta-Analysis.
72. Association between Interleukin-6 rs1800795 Polymorphism and Serum Interleukin-6 Levels and Full-Term Neonatal Sepsis.
73. The impact and interaction of TLR2 and IRF-5 gene polymorphisms on susceptibility to neonatal sepsis.
74. The correlation between 74-IL-10 gene polymorphism and susceptibility to sepsis in full-term newborns.
75. The correlation between 74-IL-10 gene polymorphism and susceptibility to sepsis in full-term newborns.

**Review,systematic analysis, animal experiment（6）：**

1. Association between lymphotoxin-α intron +252 polymorphism and sepsis: a meta-analysis.
2. Association between IL-6-174G/C Polymorphism and the Risk of Sepsis and Mortality: A Systematic Review and Meta-Analysis.
3. Periodontitis and gestational diabetes mellitus: a systematic review and meta-analysis of observational studies.
4. Prenatal pesticide exposure associated with glycated haemoglobin and markers of metabolic dysfunction in adolescents.
5. Association of gene polymorphism of bactericidal permeability increasing protein rs4358188, cluster of differentiation 14 rs2569190, interleukin 1β rs1143643 and matrix metalloproteinase-16 rs2664349 with neonatal sepsis.
6. Association of IL-6-174G &gt; C Polymorphism with Susceptibility to Childhood Sepsis: A Systematic Review and Meta-Analysis.

**Summarize(4)：**

1. Molecular biology on the ICU - From understanding to treating sepsis.
2. Prediction of sepsis-related outcomes in neonates through systematic genotyping of polymorphisms in genes for innate immunity and inflammation: a narrative review and critical perspective.
3. Elucidating the role of genomics in neonatal sepsis.
4. Research progress on the relationship between susceptibility to neonatal sepsis and polymorphisms of tumor necrosis factor and interleukin genes.

**Studies that include on the title and abstract either having no connection with the subject（143）：**

1. 4G/5G promoter polymorphism in the plasminogen-activator-inhibitor-1 gene and outcome of meningococcal disease. Meningococcal Research Group
2. Pilot study assessing TNF gene polymorphism as a prognostic marker for disease progression in neonates with sepsis
3. A family with delayed Bruton syndrome.
4. Severity of meningococcal disease in children and the angiotensin-converting enzyme insertion/deletion polymorphism.
5. Bench-to-bedside review: genetic influences on meningococcal disease.
6. Is interleukin-6 -174 genotype associated with the development of septicemia in preterm infants?
7. Lower prevalence of IL-4 receptor alpha-chain gene G variant in very-low-birth-weight infants with necrotizing enterocolitis.
8. Mutations of genes involved in the innate immune system as predictors of sepsis in very low birth weight infants.
9. The role of molecular genetics in the pathogenesis and diagnosis of neonatal sepsis.
10. Polymorphisms of genes involved in innate immunity: association with preterm delivery.
11. Tumor necrosis factor alpha -- 308 polymorphism associated with increased sepsis mortality in ventilated very low birth weight infants.
12. Evaluation of systemic inflammatory responses in neonates with herpes simplex virus infection.
13. Genetic screening for susceptibility to infection in the NICU setting.
14. Virulence and cord blood mononuclear cells cytokine production induced by perinatal listeria monocytogenes strains from different phylogenetic lineages.
15. Association of two tumour necrosis factor gene polymorphisms with the incidence of severe intraventricular haemorrhage in preterm infants.
16. Periodontal therapy reduces the rate of preterm low birth weight in women with pregnancy-associated gingivitis.
17. A macrophage migration inhibitory factor promoter polymorphism is associated with high-density parasitemia in children with malaria.
18. Interleukin-6-174-genotype, sepsis and cerebral injury in very low birth weight infants.
19. Genetic polymorphisms and infections.
20. Prevalence of two tumor necrosis factor gene polymorphisms in premature infants with early onset sepsis.
21. Genetic Polymorphisms of CD14, toll-like receptor 4, and caspase-recruitment domain 15 are not associated with necrotizing enterocolitis in very low birth weight infants.
22. 4G4G genotype of the plasminogen activator inhibitor-1 promoter polymorphism associates with disseminated intravascular coagulation in children with systemic meningococcemia.
23. Genetic association studies in VLBW infants exemplifying susceptibility to sepsis -: recent findings and implications for future research.
24. Acute inflammation is exacerbated in mice genetically predisposed to a severe protein C deficiency.
25. Investigation of the Role of Pen-Like Receptors in the Development of Immunopathogenic Intestinal Diseases.
26. The extent to which genotype information may add to the prediction of distrurbed perinatal adaption:: none, minor, or major?
27. Sudden infant death syndrome: Review of implicated genetic factors.
28. Basic and clinical research on retinopathy in premature infants.
29. Role of angiotensin-converting enzyme gene polymorphisms in children with sepsis and septic shock.
30. The role of mannose-binding lectin in susceptibility to infection in preterm Neonates.
31. A study on risk factors associated with PROM and methods for detection of BV flora.
32. 159C&gt;T CD14 genotype -: Functional effects on innate immune responses in term neonates.
33. An infectious aetiology of sudden infant death syndrome.
34. Susceptibility to meningococcal infection-Nature versus nurture?
35. [Genetic markers of predisposition to infectious complications in neonatal infants with respiratory distress syndrome.]
36. Genetics of susceptibility to malaria related phenotypes.
37. Immaturity, perinatal inflammation, and retinopathy of prematurity: A multi-hit hypothesis.
38. Effects of RANTES and MBL2 gene polymorphisms in sickle cell disease clinical outcomes: association of the g.In1.1T>C RANTES variant with protection against infections.
39. Cytokine gene polymorphisms in preterm infants with necrotising enterocolitis: genetic association study.
40. Lack of association between TLR4 polymorphism and severe gram-negative bacterial infection in neonates.
41. Nitric oxide synthase 2A (NOS2A) polymorphisms are not associated with invasive pneumococcal disease.
42. Interleukin-6 G(-174)C polymorphism is associated with mental retardation in cystic periventricular leucomalacia in preterm infants.
43. Theme Index of Chinese Journal of Contemporary Pediatrics, Volume 12, 2010.
44. Genetic variability in complement activation modulates the systemic inflammatory response syndrome in children.
45. Genetic polymorphisms in the endotoxin receptor may influence platelet count as part of the acute phase response in critically ill children.
46. [Correlations between serum interleukin-18 (IL-18) level, IL-18 gene promoter polymorphisms and the development of sepsis in children.]
47. Common <i>NFKBIL2</i> polymorphisms and susceptibility to pneumococcal disease: a genetic association study.
48. Association of the+874 T/A interferon gamma polymorphism with infections in sickle cell disease.
49. CCR5, RANTES and SDF-1 polymorphisms and mother-to-child HIV-1 transmission.
50. β<sub>2</sub>-Adrenergic Receptor Gene Polymorphism Is Associated with Mortality in Septic Shock.
51. Human Cytomegalovirus UL144 Is Associated with Viremia and Infant Development Sequelae in Congenital Infection.
52. Study on the virulence related functions of outer membrane protein T of Escherichia coli K1 pathogenic strain.
53. Genetic Analysis of BPD SP-B Genetic Deficiency Genes in Han Chinese and Study on the Mechanism of Action Leading to BPD.
54. Functional study of GimA, a virulence island gene of Escherichia coli in neonatal meningitis.
55. Functional Promoter Haplotypes of Interleukin-18 Condition Susceptibility to Severe Malarial Anemia and Childhood Mortality.
56. Association of environment and place of birth with asthma in Chinese immigrant children.
57. Procalcitonin versus CRP as an early indicator of fetal infection in preterm premature rupture of membranes.
58. Laboratory aid to the diagnosis and therapy of infection in the neonate.
59. Tumor necrosis factor-α promoter -308 G/A polymorphism and susceptibility to sepsis in very-low-birth-weight infants.
60. A regulatory polymorphism in promoter region of TNFR1 gene is associated with Kawasaki disease in Chinese individuals.
61. Environmental tobacco smoke and male sex modify the influence of IL-13 genetic variants on cord blood IgE levels.
62. Association of glutathione S-transferase Ω 1-1 polymorphisms (A140D and E208K) with the expression of interleukin-8 (IL-8), transforming growth factor beta (TGF-β), and apoptotic protease-activating factor 1 (Apaf-1) in humans chronically exposed to arsenic in drinking water.
63. Toll-Like 4 Receptor Variant, Asp299Gly, and Reduced Risk of Hemorrhagic Cystitis after Hematopoietic Stem Cell Transplantation.
64. Frequencies of functional caspase 12 genotypes in the North Africa population.
65. A functional microsatellite of the <i>macrophage migration inhibitory factor</i> gene associated with meningococcal disease.
66. Immunoregulatory gene polymorphisms in Japanese women with preterm births and periodontitis.
67. Intestinal barrier function in neonatal foals: Options for improvement.
68. Risk of infection and sepsis in severely injured patients related to single nucleotide polymorphisms in the lectin pathway.
69. Tumour necrosis factor gene polymorphism in dengue infection: association with risk of bleeding.
70. Effects of <i>IL</i>-<i>10</i> Haplotype and Atomic Bomb Radiation Exposure on Gastric Cancer Risk.
71. Neonatal infections in Saudi Arabia: association with C-reactive protein, <i>CRP</i>-286 (C&gt;T&gt;A) gene polymorphism and IgG antibodies.
72. Biomarkers for Prediction and Diagnosis of Necrotizing Enterocolitis.
73. Toll-like receptors in Neonatal Sepsis.
74. Air pollution, inflammation and preterm birth in Mexico City: Study design and methods.
75. Short (GT)n Microsatellite Repeats in the Heme Oxygenase-1 Gene Promoter Are Associated with Antioxidant and Anti-Inflammatory Status in Mexican Pediatric Patients with Sepsis.
76. IL-6、IL-10、IL-18、IFN-γ The detection significance of waiting in children with hemophagocytic syndrome.
77. Clinical and basic research on perioperative damage to important organs in children with congenital heart disease.
78. Parto Prematuro - Estudo epidemiológico e genético. <i>O Envolvimento Do Gene HBD1</i>.
79. Genetic Polymorphisms and Sepsis in Premature Neonates.
80. Gene Polymorphisms and Febrile Neutropenia in Acute Leukemia-No Association with IL-4, CCR-5, IL-1RA, but the MBL-2, ACE, and TLR-4 Are Associated with the Disease in Turkish Patients: A Preliminary Study.
81. Genetic and Environmental Influences on the Prospective Correlation Between Systemic Inflammation and Coronary Heart Disease Death in Male Twins.
82. Neonatal infections in Saudi Arabia: Association with cytokine gene polymorphisms.
83. Association of Polymorphisms in IRAK1, IRAK4 and MyD88, and Severe Invasive Pneumococcal Disease.
84. Are Immune Modulating Single Nucleotide Polymorphisms Associated with Necrotizing Enterocolitis?
85. Clinical relevance of single nucleotide polymorphisms within the 13 cytokine genes in North Indian trauma hemorrhagic shock patients.
86. Polymorphism rs2239185 in <i>vitamin D receptor</i> gene is associated with severe community-acquired pneumonia of children in Chinese Han population: a case-control study.
87. Urinary Proteins, Vitamin D and Genetic Polymorphisms as Risk Factors for Febrile Urinary Tract Infection and Relation with Bacteremia: A Case Control Study.
88. The role of inflammatory response in the occurrence and development of immature brain injury.
89. The significance of pre B cell colony enhancing factor in the diagnosis and treatment of neonatal sepsis.
90. Research progress on heart rate variability in neonatal sepsis.
91. Immune mechanism and clinical study of childhood infection.
92. Interleukin-10-1082 G/A gene polymorphisms in Egyptian children with CAP: A case-control study.
93. Genotyping of vitamin D receptor gene polymorphisms using mismatched amplification mutation assay in neonatal sepsis patients of Odisha, eastern India.
94. Does Haptoglobin Phenotype Influence Postnatal Morbidity in Preterm Neonates?
95. Association of TLR polymorphisms with bronchopulmonary dysplasia.
96. Mannose-binding lectin (MBL) insufficiency protects against the development of systemic inflammatory response after pediatric cardiac surgery.
97. Recent developments in severe sepsis research: from bench to bedside and back.
98. Isolation and identification of Streptococcus agalactiae subsp. Pasteuri, and study on its pathogenicity and mechanism of macrolide resistance.
99. Maternal Interleukin Genotypes Are Associated With NICU Outcomes Among Low-Birth-Weight Infants.
100. Genetic Polymorphisms and Bacterial Infections in Neonates.
101. Plasma levels of Macrophage Migration inhibitory Factor and D-Dopachrome Tautomerase show a highly specific Profile in early life.
102. Genome-wide association study of sepsis in extremely premature infants.
103. Pathogenicity study of foodborne Cronobacter sakazakii based on suckling mice.
104. Risk of nontyphoidal Salmonella bacteraemia in African children is modified by STAT4.
105. The interleukin-27 -964A>G polymorphism enhances sepsis-induced inflammatory responses and confers susceptibility to the development of sepsis.
106. <i>IL</i>-<i>10RA</i> Mutation as a Risk Factor of Severe Influenza-Associated Encephalopathy: A Case Report.
107. Effect of maternal and neonatal interleukin-6-174 G/C polymorphism on preterm birth and neonatal morbidity.
108. Genetic susceptibility to invasive pneumococcal disease.
109. Candidate gene analysis in pathogenesis of surgically and non-surgically treated necrotizing enterocolitis in preterm infants.
110. Clinical Predictors of Liver Fibrosis in Patients With Chronic Hepatitis B Virus Infection From Children to Adults.
111. Establishment of a humanized mouse model and preliminary exploration of gene therapy research using this model, as well as the clinical significance of interleukin-35 in the diagnosis of early-onset neonatal sepsis.
112. Establishment of Microdroplet Digital PCR Detection Method for Group B Streptococcus (GBS) and Preliminary Study on Inducing CD4+T Cell Differentiation in Newborns.
113. Research progress in the treatment of alcoholic liver disease based on the hepatointestinal axis.
114. Analysis of Molecular Epidemic Characteristics and Biofilm Formation of Infectious Streptococcus agalactiae.
115. Platelet Glycoprotein VI Haplotypes and the Presentation of Paediatric Sepsis.
116. A prospective investigation of interleukin-8 levels in pediatric acute respiratory failure and acute respiratory distress syndrome.
117. A Toll-like receptor 2 genetic variant modulates occurrence of bacterial infections in patients with sickle cell disease.
118. Acute phase reactant serum amyloid A in inflammation and other diseases.
119. Prevention and treatment of complications related to premature infants.
120. Relationship between IRAK-M gene polymorphism in umbilical cord blood and premature birth and major complications in newborns.
121. Study on the role of novel combination biomarkers in early warning of sepsis in children.
122. Proprotein Convertase Subtilisin/Kexin Type 9 Loss-of-Function Is Detrimental to the Juvenile Host With Septic Shock.
123. Is bronchopulmonary dysplasia decided before birth?
124. Two Faces of Heme Catabolic Pathway in Newborns: A Potential Role of Bilirubin and Carbon Monoxide in Neonatal Inflammatory Diseases.
125. Genetic variant rs16944 in IL1B gene is a risk factor for early-onset sepsis susceptibility and outcome in preterm infants.
126. Predicting Severe Enterovirus 71-Infected Hand, Foot, and Mouth Disease: Cytokines and Chemokines.
127. [Association between interleukin-8 rs4073 polymorphisms and susceptibility to neonatal sepsis.]
128. IL-1 β A study on the correlation between gene single nucleotide polymorphism and sepsis in full-term newborns.
129. The relationship between the rs4073 polymorphism of IL-8 gene and susceptibility to neonatal sepsis.
130. Comparative genomic study of oral streptococcus and pathogenicity analysis of periodontitis in vivo by Fusobacterium nucleatum.
131. Association between single nucleotide polymorphisms and viral load in congenital cytomegalovirus infection.
132. A case of interleukin-1 receptor associated kinase 4 deficiency and literature review.
133. A Polymorphism of Bactericidal/Permeability-Increasing Protein Affects Its Neutralization Efficiency towards Lipopolysaccharide.
134. Component 1 Inhibitor Missense (Val480Met) Variant Is Associated With Gene Expression and Sepsis Development in Neonatal Lung Disease.
135. Gene-environment interactions related to maternal exposure to environmental and lifestyle-related chemicals during pregnancy and the resulting adverse fetal growth: a review.
136. Pb and Cd exposure linked with Il-10 and Il-13 gene polymorphisms in asthma risk relevant immunomodulation in children
137. Association between Interleukin-6 rs1800795 Polymorphism and Serum Interleukin-6 Levels and Full-Term Neonatal Sepsis.
138. Association between plasminogen activator inhibitor-1 gene polymorphism and susceptibility to neonatal sepsis.
139. Association between interleukin-27 gene polymorphisms and Plasmodium falciparum Malaria.
140. Biomarkers of necrotizing enterocolitis in the era of machine learning and omics.
141. The impact and interaction of TLR2 and IRF-5 gene polymorphisms on the susceptibility to neonatal sepsis.
142. Association of 140-IRF5 gene polymorphism with sICAM1 and 25 (OH) D and neonatal pulmonary infection.
143. The impact and interaction of TLR2 and IRF-5 gene polymorphisms on susceptibility to neonatal sepsis.

**TNF-α**

**Duplicates(62):**

1. Pilot study assessing TNF gene polymorphism as a prognostic marker for disease progression in neonates with sepsis.
2. Pilot study assessing TNF gene polymorphism as a prognostic marker for disease progression in neonates with sepsis.
3. Genetic variants of TNF-[FC12]a, IL-1beta, IL-4 receptor [FC12]a-chain, IL-6 and IL-10 genes are not risk factors for sepsis in low-birth-weight infants.
4. Genetic variants of TNF-[FC12]a, IL-1beta, IL-4 receptor [FC12]a-chain, IL-6 and IL-10 genes are not risk factors for sepsis in low-birth-weight infants.
5. Association between IL-1ra gene polymorphism and premature delivery.
6. Association between IL-1ra gene polymorphism and premature delivery.
7. Tumor necrosis factor alpha -- 308 polymorphism associated with increased sepsis mortality in ventilated very low birth weight infants.
8. Cytokine responses and sudden infant death syndrome: genetic, developmental, and environmental risk factors.
9. Cytokine responses and sudden infant death syndrome: genetic, developmental, and environmental risk factors.
10. Genetic polymorphisms and risk for acute renal failure in preterm neonates.
11. Genetic polymorphisms and risk for acute renal failure in preterm neonates.
12. Interleukin-10 and its role in clinical immunoparalysis following pediatric cardiac surgery.
13. Interleukin-10 and its role in clinical immunoparalysis following pediatric cardiac surgery.
14. IL-10, IL-6 and CD14 polymorphisms and sepsis outcome in ventilated very low birth weight infants.
15. IL-10, IL-6 and CD14 polymorphisms and sepsis outcome in ventilated very low birth weight infants.
16. Prevalence of two tumor necrosis factor gene polymorphisms in premature infants with early onset sepsis.
17. Prevalence of two tumor necrosis factor gene polymorphisms in premature infants with early onset sepsis.
18. Effect of various genetic polymorphisms on the incidence and outcome of severe sepsis.
19. Effect of various genetic polymorphisms on the incidence and outcome of severe sepsis.
20. Genetic polymorphisms of CD14, toll-like receptor 4, and caspase-recruitment domain 15 are not associated with necrotizing enterocolitis in very low birth weight infants.
21. Genetic basis for necrotizing enterocolitis - Risk factors and their relations to genetic polymorphisms.
22. Genetic basis for necrotizing enterocolitis - Risk factors and their relations to genetic polymorphisms.
23. Molecular biology on the ICU - From understanding to treating sepsis.
24. Genetic association studies in VLBW infants exemplifying susceptibility to sepsis -: recent findings and implications for future research.
25. Interleukin-6 gene variants and the risk of sepsis development in children.
26. Interleukin-6 gene variants and the risk of sepsis development in children.
27. Interleukin-6 polymorphism is associated with chorioamnionitis and neonatal infections in preterm infants.
28. Interleukin-6 polymorphism is associated with chorioamnionitis and neonatal infections in preterm infants.
29. Role of polymorphic variants as genetic modulators of infection in neonatal sepsis.
30. Role of polymorphic variants as genetic modulators of infection in neonatal sepsis.
31. An age-related decrease in factor V Leiden frequency among Polish subjects.
32. An age-related decrease in factor V Leiden frequency among Polish subjects.
33. Human cytomegalovirus UL144 is associated with viremia and infant development sequelae in congenital infection.
34. Tumor necrosis factor-α promoter-308 G/A polymorphism and susceptibility to sepsis in very-low-birth-weight infants.
35. Association between lymphotoxin-α intron +252 polymorphism and sepsis: a meta-analysis.
36. Association between bronchopulmonary dysplasia and MBL2 and IL1-RN polymorphisms.
37. Association between bronchopulmonary dysplasia and MBL2 and IL1-RN polymorphisms.
38. Toll-like 4 receptor variant, Asp299Gly, and reduced risk of hemorrhagic cystitis after hematopoietic stem cell transplantation.
39. Frequencies of functional caspase 12 genotypes in the North Africa population.
40. Genetic association study of tumor necrosis factor-alpha with sepsis and septic shock in Thai pediatric patients.
41. Characterization of the acute phase response in critically ill children.
42. Characterization of the acute phase response in critically ill children.
43. Prediction of sepsis-related outcomes in neonates through systematic genotyping of polymorphisms in genes for innate immunity and inflammation: a narrative review and critical perspective.
44. Prediction of sepsis-related outcomes in neonates through systematic genotyping of polymorphisms in genes for innate immunity and inflammation: a narrative review and critical perspective.
45. Neonatal infections in Saudi Arabia: association with C-reactive protein, CRP -286 (C>T>A) gene polymorphism and IgG antibodies.
46. Neonatal infections in Saudi Arabia: association with C-reactive protein, CRP -286 (C>T>A) gene polymorphism and IgG antibodies.
47. Association of Polymorphisms in IRAK1, IRAK4 and MyD88, and Severe Invasive Pneumococcal Disease.
48. Research progress on heart rate variability in neonatal sepsis.
49. Systematic Review and Meta-analysis: Gene Association Studies in Neonatal Sepsis.
50. Systematic Review and Meta-analysis: Gene Association Studies in Neonatal Sepsis.
51. Tumor necrosis factor-α -308G/A and -238G/A polymorphisms are associated with increased risks of sepsis: evidence from an updated meta-analysis.
52. Tumor necrosis factor-α -308G/A and -238G/A polymorphisms are associated with increased risks of sepsis: evidence from an updated meta-analysis.
53. Research progress in the treatment of alcoholic liver disease based on the hepatointestinal axis.
54. The association between interleukin-6 gene -174G/C single nucleotide polymorphism and sepsis: an updated meta-analysis with trial sequential analysis.
55. The association between interleukin-6 gene -174G/C single nucleotide polymorphism and sepsis: an updated meta-analysis with trial sequential analysis.
56. Association between Tumor Necrosis Factor-α Promoter -308 G/A Polymorphism and Early Onset Sepsis in Preterm Infants.
57. Association between Tumor Necrosis Factor-α Promoter -308 G/A Polymorphism and Early Onset Sepsis in Preterm Infants.
58. Research progress on the relationship between susceptibility to neonatal sepsis and polymorphisms of tumor necrosis factor and interleukin genes.
59. Research progress on the relationship between susceptibility to neonatal sepsis and polymorphisms of tumor necrosis factor and interleukin genes.
60. Proprotein Convertase Subtilisin/Kexin Type 9 Loss-of-Function Is Detrimental to the Juvenile Host With Septic Shock*.
61. Genetic variant rs16944 in <i>IL1B</i> gene is a risk factor for early-onset sepsis susceptibility and outcome in preterm infants.

The impact and interaction of TLR2 and IRF-5 gene polymorphisms on susceptibility to neonatal sepsis.

**Review,systematic analysis, animal experiment（6）：**

1. Interleukin-6 (-174C) polymorphism and the risk of sepsis in very low birth weight infants: meta-analysis.
2. Association between lymphotoxin-alpha intron +252 polymorphism and sepsis: a meta-analysis.
3. Association between IL-6-174G/C Polymorphism and the Risk of Sepsis and Mortality: A Systematic Review and Meta-Analysis.
4. Periodontitis and gestational diabetes mellitus: a systematic review and meta-analysis of observational studies.
5. Association of Gene Polymorphism of Bactericidal Permeability Increasing Protein Rs4358188, Cluster of Differentiation 14 Rs2569190, Interleukin 1beta Rs1143643 and Matrix Metalloproteinase-16 Rs2664349 with Neonatal Sepsis.
6. Association of IL-6 -174G > C Polymorphism with Susceptibility to Childhood Sepsis: A Systematic Review and Meta-Analysis.

**Summarize(5)：**

1. Molecular biology on the ICU. From understanding to treating sepsis.
2. Prediction of sepsis-related outcomes in neonates through systematic genotyping of polymorphisms in genes for innate immunity and inflammation: A narrative review and critical perspective.
3. Elucidating the role of genomics in neonatal sepsis.
4. Research progress on the relationship between susceptibility to neonatal sepsis and polymorphisms of tumor necrosis factor and interleukin genes.
5. A narrative review of precision medicine in neonatal sepsis: genetic and epigenetic factors associated with disease susceptibility.

**Studies that include on the title and abstract either having no connection with the subject（112）：**

1. Variation in the tumor necrosis factor-alpha gene promoter region may be associated with death from meningococcal disease.
2. Pilot study assessing TNF gene polymorphism as a prognostic marker for disease progression in neonates with sepsis.
3. A family with delayed Bruton syndrome.
4. Severity of meningococcal disease in children and the angiotensin-converting enzyme insertion/deletion polymorphism.
5. Bench-to-bedside review: genetic influences on meningococcal disease.
6. Is interleukin-6 -174 genotype associated with the development of septicemia in preterm infants?
7. Lower prevalence of IL-4 receptor alpha-chain gene G variant in very-low-birth-weight infants with necrotizing enterocolitis.
8. The role of molecular genetics in the pathogenesis and diagnosis of neonatal sepsis.
9. Evaluation of systemic inflammatory responses in neonates with herpes simplex virus infection.
10. Virulence and cord blood mononuclear cells cytokine production induced by perinatal listeria monocytogenes strains from different phylogenetic lineages.
11. Association of two tumour necrosis factor gene polymorphisms with the incidence of severe intraventricular haemorrhage in preterm infants.
12. Periodontal therapy reduces the rate of preterm low birth weight in women with pregnancy-associated gingivitis.
13. A macrophage migration inhibitory factor promoter polymorphism is associated with high-density parasitemia in children with malaria.
14. Genetic polymorphisms and infections.
15. Genetic Polymorphisms of CD14, toll-like receptor 4, and caspase-recruitment domain 15 are not associated with necrotizing enterocolitis in very low birth weight infants.
16. Genetic association studies in VLBW infants exemplifying susceptibility to sepsis -: recent findings and implications for future research.
17. Acute inflammation is exacerbated in mice genetically predisposed to a severe protein C deficiency.
18. Investigation of the Role of Pen-Like Receptors in the Development of Immunopathogenic Intestinal Diseases.
19. The extent to which genotype information may add to the prediction of distrurbed perinatal adaption:: none, minor, or major?
20. Basic and clinical research on retinopathy in premature infants.
21. Role of angiotensin-converting enzyme gene polymorphisms in children with sepsis and septic shock.
22. The role of mannose-binding lectin in susceptibility to infection in preterm Neonates.
23. 159C&gt;T CD14 genotype -: Functional effects on innate immune responses in term neonates.
24. An infectious aetiology of sudden infant death syndrome.
25. [Genetic markers of predisposition to infectious complications in neonatal infants with respiratory distress syndrome.]
26. Genetics of susceptibility to malaria related phenotypes.
27. Immaturity, perinatal inflammation, and retinopathy of prematurity: A multi-hit hypothesis.
28. Effects of RANTES and MBL2 gene polymorphisms in sickle cell disease clinical outcomes: association of the g.In1.1T>C RANTES variant with protection against infections.
29. Cytokine gene polymorphisms in preterm infants with necrotising enterocolitis: genetic association study.
30. Lack of association between TLR4 polymorphism and severe gram-negative bacterial infection in neonates.
31. Nitric oxide synthase 2A (NOS2A) polymorphisms are not associated with invasive pneumococcal disease.
32. Interleukin-6 G(-174)C polymorphism is associated with mental retardation in cystic periventricular leucomalacia in preterm infants.
33. Genetic variability in complement activation modulates the systemic inflammatory response syndrome in children.
34. Genetic polymorphisms in the endotoxin receptor may influence platelet count as part of the acute phase response in critically ill children.
35. Common <i>NFKBIL2</i> polymorphisms and susceptibility to pneumococcal disease: a genetic association study.
36. Association of the+874 T/A interferon gamma polymorphism with infections in sickle cell disease.
37. CCR5, RANTES and SDF-1 polymorphisms and mother-to-child HIV-1 transmission.
38. Interaction between early maternal smoking and variants in <i>TNF</i> and <i>GSTP1</i> in childhood wheezing.
39. Human Cytomegalovirus UL144 Is Associated with Viremia and Infant Development Sequelae in Congenital Infection.
40. Study on the virulence related functions of outer membrane protein T of Escherichia coli K1 pathogenic strain.
41. Genetic Analysis of BPD SP-B Genetic Deficiency Genes in Han Chinese and Study on the Mechanism of Action Leading to BPD.
42. Functional study of GimA, a virulence island gene of Escherichia coli in neonatal meningitis.
43. Functional Promoter Haplotypes of Interleukin-18 Condition Susceptibility to Severe Malarial Anemia and Childhood Mortality.
44. Association of environment and place of birth with asthma in Chinese immigrant children.
45. Laboratory aid to the diagnosis and therapy of infection in the neonate.
46. A regulatory polymorphism in promoter region of TNFR1 gene is associated with Kawasaki disease in Chinese individuals.
47. Association of glutathione S-transferase Ω 1-1 polymorphisms (A140D and E208K) with the expression of interleukin-8 (IL-8), transforming growth factor beta (TGF-β), and apoptotic protease-activating factor 1 (Apaf-1) in humans chronically exposed to arsenic in drinking water.
48. Toll-Like 4 Receptor Variant, Asp299Gly, and Reduced Risk of Hemorrhagic Cystitis after Hematopoietic Stem Cell Transplantation.
49. Frequencies of functional caspase 12 genotypes in the North Africa population.
50. A functional microsatellite of the <i>macrophage migration inhibitory factor</i> gene associated with meningococcal disease.
51. Immunoregulatory gene polymorphisms in Japanese women with preterm births and periodontitis.
52. Intestinal barrier function in neonatal foals: Options for improvement.
53. Risk of infection and sepsis in severely injured patients related to single nucleotide polymorphisms in the lectin pathway.
54. Tumour necrosis factor gene polymorphism in dengue infection: association with risk of bleeding.
55. TNF-238 polymorphism may predict bronchopulmonary dysplasia among preterm infants in the Egyptian population.
56. Neonatal infections in Saudi Arabia: association with C-reactive protein, <i>CRP</i>-286 (C&gt;T&gt;A) gene polymorphism and IgG antibodies.
57. Biomarkers for Prediction and Diagnosis of Necrotizing Enterocolitis.
58. Toll-like receptors in Neonatal Sepsis.
59. Air pollution, inflammation and preterm birth in Mexico City: Study design and methods.
60. Short (GT)n Microsatellite Repeats in the Heme Oxygenase-1 Gene Promoter Are Associated with Antioxidant and Anti-Inflammatory Status in Mexican Pediatric Patients with Sepsis.
61. IL-6、IL-10、IL-18、IFN-γThe detection significance of waiting in children with hemophagocytic syndrome.
62. Parto Prematuro - Estudo epidemiológico e genético. <i>O Envolvimento Do Gene HBD1</i>.
63. A Single Nucleotide Polymorphism in the Corticotropin Receptor Gene Is Associated With a Blunted Cortisol Response During Pediatric Critical Illness.
64. Association of Polymorphisms in IRAK1, IRAK4 and MyD88, and Severe Invasive Pneumococcal Disease.
65. Are Immune Modulating Single Nucleotide Polymorphisms Associated with Necrotizing Enterocolitis?
66. Clinical relevance of single nucleotide polymorphisms within the 13 cytokine genes in North Indian trauma hemorrhagic shock patients.
67. Polymorphism rs2239185 in <i>vitamin D receptor</i> gene is associated with severe community-acquired pneumonia of children in Chinese Han population: a case-control study.
68. Urinary Proteins, Vitamin D and Genetic Polymorphisms as Risk Factors for Febrile Urinary Tract Infection and Relation with Bacteremia: A Case Control Study.
69. The role of inflammatory response in the occurrence and development of immature brain injury.
70. The significance of pre B cell colony enhancing factor in the diagnosis and treatment of neonatal sepsis.
71. Immune mechanism and clinical study of childhood infection.
72. Research progress on heart rate variability in neonatal sepsis.
73. Interleukin-10-1082 G/A gene polymorphisms in Egyptian children with CAP: A case-control study.
74. Genotyping of vitamin D receptor gene polymorphisms using mismatched amplification mutation assay in neonatal sepsis patients of Odisha, eastern India.
75. Does Haptoglobin Phenotype Influence Postnatal Morbidity in Preterm Neonates?
76. Mannose-binding lectin (MBL) insufficiency protects against the development of systemic inflammatory response after pediatric cardiac surgery.
77. Recent developments in severe sepsis research: from bench to bedside and back.
78. Isolation and identification of Streptococcus agalactiae subsp. Pasteuri, and study on its pathogenicity and mechanism of macrolide resistance.
79. Maternal Interleukin Genotypes Are Associated With NICU Outcomes Among Low-Birth-Weight Infants.
80. Genetic Polymorphisms and Bacterial Infections in Neonates.
81. Plasma levels of Macrophage Migration inhibitory Factor and D-Dopachrome Tautomerase show a highly specific Profile in early life.
82. Pathogenicity study of foodborne Cronobacter sakazakii based on suckling mice.
83. Risk of nontyphoidal Salmonella bacteraemia in African children is modified by STAT4.
84. The interleukin-27 -964A>G polymorphism enhances sepsis-induced inflammatory responses and confers susceptibility to the development of sepsis.
85. <i>IL</i>-<i>10RA</i> Mutation as a Risk Factor of Severe Influenza-Associated Encephalopathy: A Case Report.
86. Effect of maternal and neonatal interleukin-6-174 G/C polymorphism on preterm birth and neonatal morbidity.
87. Genetic susceptibility to invasive pneumococcal disease.
88. Candidate gene analysis in pathogenesis of surgically and non-surgically treated necrotizing enterocolitis in preterm infants.
89. Clinical Predictors of Liver Fibrosis in Patients With Chronic Hepatitis B Virus Infection From Children to Adults.
90. Establishment of a humanized mouse model and preliminary exploration of gene therapy research using this model, as well as the clinical significance of interleukin-35 in the diagnosis of early-onset neonatal sepsis.
91. Establishment of Microdroplet Digital PCR Detection Method for Group B Streptococcus (GBS) and Preliminary Study on Inducing CD4+T Cell Differentiation in Newborns.
92. Research progress in the treatment of alcoholic liver disease based on the hepatointestinal axis.
93. Analysis of Molecular Epidemic Characteristics and Biofilm Formation of Infectious Streptococcus agalactiae.
94. Toxicological effects in children exposed to lead: A cross-sectional study at the Colombian Caribbean coast.
95. Platelet Glycoprotein VI Haplotypes and the Presentation of Paediatric Sepsis.
96. A Toll-like receptor 2 genetic variant modulates occurrence of bacterial infections in patients with sickle cell disease.
97. Acute phase reactant serum amyloid A in inflammation and other diseases.
98. Study on the role of novel combination biomarkers in early warning of sepsis in children.
99. Proprotein Convertase Subtilisin/Kexin Type 9 Loss-of-Function Is Detrimental to the Juvenile Host With Septic Shock.
100. Is bronchopulmonary dysplasia decided before birth?
101. Two Faces of Heme Catabolic Pathway in Newborns: A Potential Role of Bilirubin and Carbon Monoxide in Neonatal Inflammatory Diseases.
102. Genetic variant rs16944 in IL1B gene is a risk factor for early-onset sepsis susceptibility and outcome in preterm infants.
103. Predicting Severe Enterovirus 71-Infected Hand, Foot, and Mouth Disease: Cytokines and Chemokines.
104. [Association between interleukin-8 rs4073 polymorphisms and susceptibility to neonatal sepsis.]
105. Comparative genomic study of oral streptococcus and pathogenicity analysis of periodontitis in vivo by Fusobacterium nucleatum.
106. Association between single nucleotide polymorphisms and viral load in congenital cytomegalovirus infection.
107. Component 1 Inhibitor Missense (Val480Met) Variant Is Associated With Gene Expression and Sepsis Development in Neonatal Lung Disease.
108. Association between Interleukin-6 rs1800795 Polymorphism and Serum Interleukin-6 Levels and Full-Term Neonatal Sepsis.
109. Association between interleukin-27 gene polymorphisms and Plasmodium falciparum Malaria.
110. The impact and interaction of TLR2 and IRF-5 gene polymorphisms on the susceptibility to neonatal sepsis.
111. Association of 140-IRF5 gene polymorphism with sICAM1 and 25 (OH) D and neonatal pulmonary infection.
112. The impact and interaction of TLR2 and IRF-5 gene polymorphisms on susceptibility to neonatal sepsis.
