# Supplementary material for: A meta-analysis of the association between inflammatory cytokine polymorphism and neonatal sepsis
Source: PLoS One. 2024 Jun 7;19(6):e0301859. doi: 10.1371/journal.pone.0301859 (PMC11161124; doi:10.1371/journal.pone.0301859)
Supplement: S2 File — (DOCX) [file pone.0301859.s002.docx]

**Meta-analysis on Genetic Association Studies Checklist | PLOS ONE**

|  | Item | Section name and paragraph number within manuscript |
| --- | --- | --- |
|  | **Introduction** |  |
| 1 | Provide a detailed justification for the polymorphism studied; if a single polymorphism was analyzed, give details as to why others were not included in the meta-analysis. | 1.Introduction、paragraph number59-66；4.1Interleukin-1 (IL-1) gene polymorphism、paragraph number635-647；4.2Interleukin-6 (IL-6) gene polymorphism、paragraph number652-657；4.3Interleukin-8 (IL-8) gene polymorphism、paragraph number665-672； 4.4Interleukin-10 (IL-10) gene polymorphism、paragraph number679-688；4.5Tumor growth factor-α (TNF-α) gene polymorphism、paragraph number690-701 |
| 2 | Provide a detailed justification for the population(s) and clinical condition studied. | 1.Introduction、paragraph number59-66 |
|  | **Methods** |  |
| 3 | Provide full details of the search strategy employed; outline the full electronic search strategy –specific combination of keywords and any limits applied- for at least one database. Specify whether synonyms of polymorphisms/genes (e.g. SNP number) were searched. | 2.1Document retrieval、paragraph number69-73；See attached search strategy |
| 4 | Report full details on the inclusion and exclusion criteria applied for selecting studies.  *Please list the excluded articles and the reasons for exclusion of each article in a supplementary file.* | 2.2Inclusion and exclusion criteria、paragraph number75-80；See the exclusion article in the attachment |
| 5 | Provide details on how the quality of the studies included in the analyses was assessed. | 2.5Quality evaluation、paragraph number108-111 |
| 6 | Describe steps taken to contact study authors to identify additional studies and to request missing data. | 2.3Literature screening、paragraph number82-84 |
| 7 | Describe how environmental effects were adjusted for, if this adjustment was not conducted, outline the reasons for this. | 4.Discussion、paragraph number702-715 |
| 8 | Describe the methods of handling heterogeneity/between-study variance. | 3.4Heterogeneity assessment、paragraph number248-261 |
| 9 | Describe how the Hardy-Weinberg equilibrium and linkage disequilibrium were assessed. | 3.2Hardy-Weinberg test、paragraph number133-135 |
| 10 | Describe and justify the choice of model for the analyses (per-allele vs per-genotype vs genetic model-free, random effects vs fixed effects). | 2.6Statistical analysis、paragraph number113-119；3.3Genetic model、paragraph number137-139 |
| 11 | Describe whether a sensitivity analysis has been completed. | 3.8Sensitivity analysis、paragraph number586-606 |
| 12 | Describe whether an assessment of the effects of population stratification has been conducted. | 3.7Subgroup analysis、paragraph number374-380 |
| 13 | Describe whether study-specific results have been assessed and if so the reasons for this (e.g. forest plot). | 3.3The relationship between various cytokine polymorphisms (IL-1, IL-6, IL-8, IL-10, TNF-α) and neonatal sepsis、paragraph number264-287 |
|  | **Results** |  |
| 14 | Include flow diagram for the studies included in the meta-analysis as the first figure for the manuscript | 2.3Literature screening、paragraph number88，91，95，99 and 103 |
| 15 | Report details on allele/genotype prevalence. | 4.1Interleukin-1 (IL-1) gene polymorphism、paragraph number628-647；4.2Interleukin-6 (IL-6) gene polymorphism、paragraph number649-657；4.3Interleukin-8 (IL-8) gene polymorphism、paragraph number659-672；4.4Interleukin-10 (IL-10) gene polymorphism、paragraph number674-688；4.5Tumor growth factor-α (TNF-α) gene polymorphism、paragraph number690-715 |
| 16 | Report the effect size estimates and p values for each analysis. | 4.1Interleukin-1 (IL-1) gene polymorphism、paragraph number628-647；4.2Interleukin-6 (IL-6) gene polymorphism、paragraph number649-657；4.3Interleukin-8 (IL-8) gene polymorphism、paragraph number659-672；4.4Interleukin-10 (IL-10) gene polymorphism、paragraph number674-688；4.5Tumor growth factor-α (TNF-α) gene polymorphism、paragraph number690-715 |
|  | **Discussion** |  |
| 17 | Discuss the limitations of the meta-analysis, including genotyping errors/bias and publication bias. | 4.Discussion、paragraph number702-715 |
| 18 | If the meta-analysis identifies an association within a subgroup of the population studied but not another, discuss the implications of these results, and if applicable the possibility of subgroup-specific publication bias. | 3.7Subgroup analysis、paragraph number374-548 |
| 19 | Discuss the suitability of the sample size employed to the research question and the power of the study. | 4.Discussion、paragraph number702-715 |
