# Supplementary material for: A meta-analysis of the association between inflammatory cytokine polymorphism and neonatal sepsis
Source: PLoS One. 2024 Jun 7;19(6):e0301859. doi: 10.1371/journal.pone.0301859 (PMC11161124; doi:10.1371/journal.pone.0301859)
Supplement: S3 File — (DOC) [file pone.0301859.s003.doc]

知网：

IL-1（5）

SU=(新生儿 + 足月儿 + 新生婴儿 + 早产儿 + 过期产儿 + 正常出生体重儿 + 低出生体重儿 + 巨大儿 + 极低出生体重儿 + 适于胎龄儿 + 小于胎龄儿 + 大于胎龄儿 + 早期新生儿 + 晚期新生儿 + 高危儿)

SU=(败血症 + 菌血症 + 脓毒败血症 + 脓毒血症 + 脓毒症)

SU=(多态性 + 基因多态性 + 遗传多态性 + 单核苷酸多态性 + DNA重复序列多态性 + DNA片段长度多态性 + 变异 + 突变 + 基因型 + 等位基因)

SU=(炎性细胞因子 + 促炎性细胞因子 + 促炎细胞因子 + 抗炎细胞因子 + 白细胞介素-1 + IL-1)

IL-6（6）

SU=(新生儿 + 足月儿 + 新生婴儿 + 早产儿 + 过期产儿 + 正常出生体重儿 + 低出生体重儿 + 巨大儿 + 极低出生体重儿 + 适于胎龄儿 + 小于胎龄儿 + 大于胎龄儿 + 早期新生儿 + 晚期新生儿 + 高危儿)

SU=(败血症 + 菌血症 + 脓毒败血症 + 脓毒血症 + 脓毒症)

SU=(多态性 + 基因多态性 + 遗传多态性 + 单核苷酸多态性 + DNA重复序列多态性 + DNA片段长度多态性 + 变异 + 突变 + 基因型 + 等位基因)

SU=(炎性细胞因子 + 促炎性细胞因子 + 促炎细胞因子 + 抗炎细胞因子 + 白细胞介素-6 + IL-6)

IL-8（5）

SU=(新生儿 + 足月儿 + 新生婴儿 + 早产儿 + 过期产儿 + 正常出生体重儿 + 低出生体重儿 + 巨大儿 + 极低出生体重儿 + 适于胎龄儿 + 小于胎龄儿 + 大于胎龄儿 + 早期新生儿 + 晚期新生儿 + 高危儿)

SU=(败血症 + 菌血症 + 脓毒败血症 + 脓毒血症 + 脓毒症)

SU=(多态性 + 基因多态性 + 遗传多态性 + 单核苷酸多态性 + DNA重复序列多态性 + DNA片段长度多态性 + 变异 + 突变 + 基因型 + 等位基因)

SU=(炎性细胞因子 + 促炎性细胞因子 + 促炎细胞因子 + 抗炎细胞因子 + 白细胞介素-8 + IL-8)

IL-10（6）

SU=(新生儿 + 足月儿 + 新生婴儿 + 早产儿 + 过期产儿 + 正常出生体重儿 + 低出生体重儿 + 巨大儿 + 极低出生体重儿 + 适于胎龄儿 + 小于胎龄儿 + 大于胎龄儿 + 早期新生儿 + 晚期新生儿 + 高危儿)

SU=(败血症 + 菌血症 + 脓毒败血症 + 脓毒血症 + 脓毒症)

SU=(多态性 + 基因多态性 + 遗传多态性 + 单核苷酸多态性 + DNA重复序列多态性 + DNA片段长度多态性 + 变异 + 突变 + 基因型 + 等位基因)

SU=(炎性细胞因子 + 促炎性细胞因子 + 促炎细胞因子 + 抗炎细胞因子 + 白细胞介素-10 + IL-10)

TNF-α（0）

SU=(新生儿 + 足月儿 + 新生婴儿 + 早产儿 + 过期产儿 + 正常出生体重儿 + 低出生体重儿 + 巨大儿 + 极低出生体重儿 + 适于胎龄儿 + 小于胎龄儿 + 大于胎龄儿 + 早期新生儿 + 晚期新生儿 + 高危儿) AND SU=(败血症 + 菌血症 + 脓毒败血症 + 脓毒血症 + 脓毒症 + 全身炎症反应) AND SU=(多态性 + 基因多态性 + 遗传多态性 + 单核苷酸多态性 + DNA重复序列多态性 + DNA片段长度多态性 + 变异 + 突变 + 基因型 + 等位基因) AND SU=(炎性细胞因子 + 促炎性细胞因子 + 促炎细胞因子 + 抗炎细胞因子 + 肿瘤坏死因子 + 肿瘤坏死因子-α + 肿瘤坏死因子-β + TNF-α + TNF-β + TNF)

**万方：**

**IL-1（28）**

[主题:(新生儿 OR 足月儿 OR 新生婴儿 OR 早产儿 OR过期产儿 OR 正常出生体重儿 OR 低出生体重儿 OR 巨大儿 OR极低出生体重儿 OR 适于胎龄儿 OR 小于胎龄儿 OR 大于胎龄儿) and 主题:(败血症 OR 菌血症 OR 脓毒败血症 OR 脓毒血症 OR 脓毒症) and 主题:( 炎性细胞因子 OR 促炎性细胞因子 OR 促炎细胞因子 OR 抗炎细胞因子 OR 白细胞介素 OR 白细胞介素-1 OR IL-1 OR 炎症因子 OR 炎性因子 OR 细胞因子) and 主题:(多态性 OR 基因多态性 OR 遗传多态性 OR 单核苷酸多态性 OR DNA重复序列多态性 OR DNA片段长度多态性 OR 变异 OR 突变 OR 基因型 OR 等位基因)](https://bnu.zssgdsb-85176920tsgjnz.com/https/77726476706e69737468656265737421e3b9569d2936695e790c88b8991b203a8f933f43/advanced-search/paper?q=主题:(新生儿 OR 足月儿 OR 新生婴儿 OR 早产儿 OR过期产儿 OR 正常出生体重儿 OR 低出生体重儿 OR 巨大儿 OR极低出生体重儿 OR 适于胎龄儿 OR 小于胎龄儿 OR 大于胎龄儿) and 主题:(败血症 OR 菌血症 OR 脓毒败血症 OR 脓毒血症 OR 脓毒症) and 主题:( 炎性细胞因子 OR 促炎性细胞因子 OR 促炎细胞因子 OR 抗炎细胞因子 OR 白细胞介素 OR 白细胞介素-1 OR IL-1 OR 炎症因子 OR 炎性因子 OR 细胞因子) and 主题:(多态性 OR 基因多态性 OR 遗传多态性 OR 单核苷酸多态性 OR DNA重复序列多态性 OR DNA片段长度多态性 OR 变异 OR 突变 OR 基因型 OR 等位基因)&type=[)

IL-6（27）

主题:(新生儿 OR 足月儿 OR 新生婴儿 OR 早产儿 OR过期产儿 OR 正常出生体重儿 OR 低出生体重儿 OR 巨大儿 OR极低出生体重儿 OR 适于胎龄儿 OR 小于胎龄儿 OR 大于胎龄儿) and 主题:(败血症 OR 菌血症 OR 脓毒败血症 OR 脓毒血症 OR 脓毒症) and 主题:( 炎性细胞因子 OR 促炎性细胞因子 OR 促炎细胞因子 OR 抗炎细胞因子 OR 白细胞介素 OR 白细胞介素-6 OR IL-6 OR 炎症因子 OR 炎性因子 OR 细胞因子) and 主题:(多态性 OR 基因多态性 OR 遗传多态性 OR 单核苷酸多态性 OR DNA重复序列多态性 OR DNA片段长度多态性 OR 变异 OR 突变 OR 基因型 OR 等位基因)

IL-8（25）

主题:(新生儿 OR 足月儿 OR 新生婴儿 OR 早产儿 OR过期产儿 OR 正常出生体重儿 OR 低出生体重儿 OR 巨大儿 OR极低出生体重儿 OR 适于胎龄儿 OR 小于胎龄儿 OR 大于胎龄儿) and 主题:(败血症 OR 菌血症 OR 脓毒败血症 OR 脓毒血症 OR 脓毒症) and 主题:( 炎性细胞因子 OR 促炎性细胞因子 OR 促炎细胞因子 OR 抗炎细胞因子 OR 白细胞介素 OR 白细胞介素-8 OR IL-8 OR 炎症因子 OR 炎性因子 OR 细胞因子) and 主题:(多态性 OR 基因多态性 OR 遗传多态性 OR 单核苷酸多态性 OR DNA重复序列多态性 OR DNA片段长度多态性 OR 变异 OR 突变 OR 基因型 OR 等位基因)

IL-10（26）

主题:(新生儿 OR 足月儿 OR 新生婴儿 OR 早产儿 OR过期产儿 OR 正常出生体重儿 OR 低出生体重儿 OR 巨大儿 OR极低出生体重儿 OR 适于胎龄儿 OR 小于胎龄儿 OR 大于胎龄儿) and 主题:(败血症 OR 菌血症 OR 脓毒败血症 OR 脓毒血症 OR 脓毒症) and 主题:( 炎性细胞因子 OR 促炎性细胞因子 OR 促炎细胞因子 OR 抗炎细胞因子 OR 白细胞介素 OR 白细胞介素-10 OR IL-10 OR 炎症因子 OR 炎性因子 OR 细胞因子) and 主题:(多态性 OR 基因多态性 OR 遗传多态性 OR 单核苷酸多态性 OR DNA重复序列多态性 OR DNA片段长度多态性 OR 变异 OR 突变 OR 基因型 OR 等位基因)

TNF（19）

主题:(新生儿 OR 足月儿 OR 新生婴儿 OR 早产儿 OR过期产儿 OR 正常出生体重儿 OR 低出生体重儿 OR 巨大儿 OR极低出生体重儿 OR 适于胎龄儿 OR 小于胎龄儿 OR 大于胎龄儿) and 主题:(败血症 OR 菌血症 OR 脓毒败血症 OR 脓毒血症 OR 脓毒症) and 主题:( 炎性细胞因子 OR 促炎性细胞因子 OR 促炎细胞因子 OR 抗炎细胞因子 OR 肿瘤坏死因子 OR TNF OR 炎症因子 OR 炎性因子 OR 细胞因子) and 主题:(多态性 OR 基因多态性 OR 遗传多态性 OR 单核苷酸多态性 OR DNA重复序列多态性 OR DNA片段长度多态性 OR 变异 OR 突变 OR 基因型 OR 等位基因)

**维普：**

**IL-1（6）**

**((((((((((((((((题名或关键词=新生儿 OR 题名或关键词=足月儿) OR 题名或关键词=新生婴儿) OR 题名或关键词=早产儿) OR 题名或关键词=过期产儿) OR 题名或关键词=正常出生体重儿) OR 题名或关键词=低出生体重儿) OR 题名或关键词=巨大儿 OR极低出生体重儿) OR 题名或关键词=适于胎龄儿) OR 题名或关键词=小于胎龄儿) OR 题名或关键词=大于胎龄儿) OR 题名或关键词=早期新生儿) OR 题名或关键词=晚期新生儿) OR 题名或关键词=高危儿) AND ((((题名或关键词=败血症 OR 题名或关键词=菌血症) OR 题名或关键词=脓毒败血症) OR 题名或关键词=脓毒血症) OR 题名或关键词=脓毒症)) AND ((((((((((((题名或关键词=炎性细胞因子 OR 题名或关键词=促炎性细胞因子) OR 题名或关键词=促炎细胞因子) OR 题名或关键词=抗炎细胞因子) OR 题名或关键词=白细胞介素) OR (题名或关键词=白细胞介素 AND ( NOT 题名或关键词=1))) OR (题名或关键词=IL AND ( NOT 题名或关键词=1))) OR 题名或关键词=干扰素) OR (题名或关键词=转化生长因子 AND ( NOT 题名或关键词=β))) OR 题名或关键词=炎症因子) OR 题名或关键词=炎性因子) OR 题名或关键词=细胞因子) OR 题名或关键词=促炎因子)) AND (((((((((题名或关键词=多态性 OR 题名或关键词=基因多态性) OR 题名或关键词=遗传多态性) OR 题名或关键词=单核苷酸多态性) OR 题名或关键词=DNA重复序列多态性) OR 题名或关键词=DNA片段长度多态性) OR 题名或关键词=变异) OR 题名或关键词=突变) OR 题名或关键词=基因型) OR 题名或关键词=等位基因)) 订阅**

**IL-6（6）**

**((((((((((((((((题名或关键词=新生儿 OR 题名或关键词=足月儿) OR 题名或关键词=新生婴儿) OR 题名或关键词=早产儿) OR 题名或关键词=过期产儿) OR 题名或关键词=正常出生体重儿) OR 题名或关键词=低出生体重儿) OR 题名或关键词=巨大儿 OR极低出生体重儿) OR 题名或关键词=适于胎龄儿) OR 题名或关键词=小于胎龄儿) OR 题名或关键词=大于胎龄儿) OR 题名或关键词=早期新生儿) OR 题名或关键词=晚期新生儿) OR 题名或关键词=高危儿) AND ((((题名或关键词=败血症 OR 题名或关键词=菌血症) OR 题名或关键词=脓毒败血症) OR 题名或关键词=脓毒血症) OR 题名或关键词=脓毒症)) AND ((((((((((((题名或关键词=炎性细胞因子 OR 题名或关键词=促炎性细胞因子) OR 题名或关键词=促炎细胞因子) OR 题名或关键词=抗炎细胞因子) OR 题名或关键词=白细胞介素) OR (题名或关键词=白细胞介素 AND ( NOT 题名或关键词=6))) OR (题名或关键词=IL AND ( NOT 题名或关键词=6))) OR 题名或关键词=干扰素) OR (题名或关键词=转化生长因子 AND ( NOT 题名或关键词=β))) OR 题名或关键词=炎症因子) OR 题名或关键词=炎性因子) OR 题名或关键词=细胞因子) OR 题名或关键词=促炎因子)) AND (((((((((题名或关键词=多态性 OR 题名或关键词=基因多态性) OR 题名或关键词=遗传多态性) OR 题名或关键词=单核苷酸多态性) OR 题名或关键词=DNA重复序列多态性) OR 题名或关键词=DNA片段长度多态性) OR 题名或关键词=变异) OR 题名或关键词=突变) OR 题名或关键词=基因型) OR 题名或关键词=等位基因))**

**IL-8（6）**

**((((((((((((((((题名或关键词=新生儿 OR 题名或关键词=足月儿) OR 题名或关键词=新生婴儿) OR 题名或关键词=早产儿) OR 题名或关键词=过期产儿) OR 题名或关键词=正常出生体重儿) OR 题名或关键词=低出生体重儿) OR 题名或关键词=巨大儿 OR极低出生体重儿) OR 题名或关键词=适于胎龄儿) OR 题名或关键词=小于胎龄儿) OR 题名或关键词=大于胎龄儿) OR 题名或关键词=早期新生儿) OR 题名或关键词=晚期新生儿) OR 题名或关键词=高危儿) AND ((((题名或关键词=败血症 OR 题名或关键词=菌血症) OR 题名或关键词=脓毒败血症) OR 题名或关键词=脓毒血症) OR 题名或关键词=脓毒症)) AND ((((((((((((题名或关键词=炎性细胞因子 OR 题名或关键词=促炎性细胞因子) OR 题名或关键词=促炎细胞因子) OR 题名或关键词=抗炎细胞因子) OR 题名或关键词=白细胞介素) OR (题名或关键词=白细胞介素 AND ( NOT 题名或关键词=8))) OR (题名或关键词=IL AND ( NOT 题名或关键词=8))) OR 题名或关键词=干扰素) OR (题名或关键词=转化生长因子 AND ( NOT 题名或关键词=β))) OR 题名或关键词=炎症因子) OR 题名或关键词=炎性因子) OR 题名或关键词=细胞因子) OR 题名或关键词=促炎因子)) AND (((((((((题名或关键词=多态性 OR 题名或关键词=基因多态性) OR 题名或关键词=遗传多态性) OR 题名或关键词=单核苷酸多态性) OR 题名或关键词=DNA重复序列多态性) OR 题名或关键词=DNA片段长度多态性) OR 题名或关键词=变异) OR 题名或关键词=突变) OR 题名或关键词=基因型) OR 题名或关键词=等位基因))**

**IL-10（6）**

**(((((((((((((((题名或关键词=新生儿 OR 题名或关键词=足月儿) OR 题名或关键词=新生婴儿) OR 题名或关键词=早产儿 OR过期产儿) OR 题名或关键词=正常出生体重儿) OR 题名或关键词=低出生体重儿) OR 题名或关键词=巨大儿 OR极低出生体重儿) OR 题名或关键词=适于胎龄儿) OR 题名或关键词=小于胎龄儿) OR 题名或关键词=大于胎龄儿) OR 题名或关键词=早期新生儿) OR 题名或关键词=晚期新生儿) OR 题名或关键词=高危儿) AND ((((题名或关键词=败血症 OR 题名或关键词=菌血症) OR 题名或关键词=脓毒败血症) OR 题名或关键词=脓毒血症) OR 题名或关键词=脓毒症)) AND ((((((((((((题名或关键词=炎性细胞因子 OR 题名或关键词=促炎性细胞因子) OR 题名或关键词=促炎细胞因子) OR 题名或关键词=抗炎细胞因子) OR 题名或关键词=白细胞介素) OR (题名或关键词=白细胞介素 AND ( NOT 题名或关键词=10))) OR (题名或关键词=IL AND ( NOT 题名或关键词=10))) OR 题名或关键词=干扰素) OR (题名或关键词=转化生长因子 AND ( NOT 题名或关键词=β))) OR 题名或关键词=炎症因子) OR 题名或关键词=炎性因子) OR 题名或关键词=细胞因子) OR 题名或关键词=促炎因子)) AND (((((((((题名或关键词=多态性 OR 题名或关键词=基因多态性) OR 题名或关键词=遗传多态性) OR 题名或关键词=单核苷酸多态性) OR 题名或关键词=DNA重复序列多态性) OR 题名或关键词=DNA片段长度多态性) OR 题名或关键词=变异) OR 题名或关键词=突变) OR 题名或关键词=基因型) OR 题名或关键词=等位基因))**

**Tnf（4）**

**(((((((((((((((题名或关键词=新生儿 OR 题名或关键词=足月儿) OR 题名或关键词=新生婴儿) OR 题名或关键词=早产儿 OR过期产儿) OR 题名或关键词=正常出生体重儿) OR 题名或关键词=低出生体重儿) OR 题名或关键词=巨大儿 OR极低出生体重儿) OR 题名或关键词=适于胎龄儿) OR 题名或关键词=小于胎龄儿) OR 题名或关键词=大于胎龄儿) OR 题名或关键词=早期新生儿) OR 题名或关键词=晚期新生儿) OR 题名或关键词=高危儿) AND ((((题名或关键词=败血症 OR 题名或关键词=菌血症) OR 题名或关键词=脓毒败血症) OR 题名或关键词=脓毒血症) OR 题名或关键词=脓毒症)) AND ((((((((((((题名或关键词=炎性细胞因子 OR 题名或关键词=促炎性细胞因子) OR 题名或关键词=促炎细胞因子) OR 题名或关键词=抗炎细胞因子) OR 题名或关键词=肿瘤坏死因子) OR (题名或关键词=肿瘤坏死因子 AND ( NOT 题名或关键词=α))) OR (题名或关键词=肿瘤坏死因子 AND ( NOT 题名或关键词=β))) OR 题名或关键词=干扰素) OR (题名或关键词=转化生长因子 AND ( NOT 题名或关键词=β))) OR 题名或关键词=炎症因子) OR 题名或关键词=炎性因子) OR 题名或关键词=细胞因子) OR 题名或关键词=促炎因子)) AND (((((((((题名或关键词=多态性 OR 题名或关键词=基因多态性) OR 题名或关键词=遗传多态性) OR 题名或关键词=单核苷酸多态性) OR 题名或关键词=DNA重复序列多态性) OR 题名或关键词=DNA片段长度多态性) OR 题名或关键词=变异) OR 题名或关键词=突变) OR 题名或关键词=基因型) OR 题名或关键词=等位基因))**

**CBM：**

**IL-1（6）**

**IL-6（6）**

**IL-8（6）**

**IL-10（6）**

**Tnf（3）**

**29 (((((("肿瘤坏死因子α"[不加权:扩展] OR "肿瘤坏死因子α"[不加权:扩展] OR "淋巴毒素α"[不加权:扩展])) OR (("炎性细胞因子"[常用字段:智能] OR "促炎性细胞因子"[常用字段:智能] OR "促炎细胞因子"[常用字段:智能] OR "抗炎细胞因子"[常用字段:智能] OR "炎症因子"[常用字段:智能] OR "炎性因子"[常用字段:智能] OR "促炎因子"[常用字段:智能] OR "细胞因子"[常用字段:智能]))))) AND ((((("多态性"[常用字段:智能] OR "基因多态性"[常用字段:智能] OR "遗传多态性"[常用字段:智能] OR "变异"[常用字段:智能] OR "突变"[常用字段:智能] OR "基因型"[常用字段:智能] OR "等位基因"[常用字段:智能] OR "DNA重复序列多态性"[常用字段:智能] OR "DNA片段长度多态性"[常用字段:智能])) AND ((((((((("婴儿, 新生"[不加权:扩展]) OR ("早产儿"[常用字段:智能] OR "足月儿"[常用字段:智能] OR "新生儿"[常用字段:智能] OR "新生婴儿"[常用字段:智能] OR "正常出生体重儿"[常用字段:智能] OR "低出生体重儿"[常用字段:智能] OR "巨大儿"[常用字段:智能] OR "极低出生体重儿"[常用字段:智能] OR "小于胎龄儿"[常用字段:智能]))) AND ((("菌血症"[常用字段:智能] OR "脓毒败血症"[常用字段:智能] OR "脓毒血症"[常用字段:智能]) OR ("脓毒症"[不加权:扩展] OR "出血性败血症"[不加权:扩展])))))) OR (("新生儿败血症"[常用字段:智能] OR "新生儿脓毒血症"[常用字段:智能] OR "新生儿菌血症"[常用字段:智能] OR "新生儿脓毒败血症"[常用字段:智能]))))))))) 13 21:39:20**

**28 ((("肿瘤坏死因子α"[不加权:扩展] OR "肿瘤坏死因子α"[不加权:扩展] OR "淋巴毒素α"[不加权:扩展])) OR (("炎性细胞因子"[常用字段:智能] OR "促炎性细胞因子"[常用字段:智能] OR "促炎细胞因子"[常用字段:智能] OR "抗炎细胞因子"[常用字段:智能] OR "炎症因子"[常用字段:智能] OR "炎性因子"[常用字段:智能] OR "促炎因子"[常用字段:智能] OR "细胞因子"[常用字段:智能]))) 499815 21:38:26**

**27 ((((((("白细胞介素类"[不加权:扩展]) OR ("白细胞介素10"[不加权:扩展]))) OR (("炎性细胞因子"[常用字段:智能] OR "促炎性细胞因子"[常用字段:智能] OR "促炎细胞因子"[常用字段:智能] OR "抗炎细胞因子"[常用字段:智能] OR "炎症因子"[常用字段:智能] OR "炎性因子"[常用字段:智能] OR "促炎因子"[常用字段:智能] OR "细胞因子"[常用字段:智能]))))) AND ((((("多态性"[常用字段:智能] OR "基因多态性"[常用字段:智能] OR "遗传多态性"[常用字段:智能] OR "变异"[常用字段:智能] OR "突变"[常用字段:智能] OR "基因型"[常用字段:智能] OR "等位基因"[常用字段:智能] OR "DNA重复序列多态性"[常用字段:智能] OR "DNA片段长度多态性"[常用字段:智能])) AND ((((((((("婴儿, 新生"[不加权:扩展]) OR ("早产儿"[常用字段:智能] OR "足月儿"[常用字段:智能] OR "新生儿"[常用字段:智能] OR "新生婴儿"[常用字段:智能] OR "正常出生体重儿"[常用字段:智能] OR "低出生体重儿"[常用字段:智能] OR "巨大儿"[常用字段:智能] OR "极低出生体重儿"[常用字段:智能] OR "小于胎龄儿"[常用字段:智能]))) AND ((("菌血症"[常用字段:智能] OR "脓毒败血症"[常用字段:智能] OR "脓毒血症"[常用字段:智能]) OR ("脓毒症"[不加权:扩展] OR "出血性败血症"[不加权:扩展])))))) OR (("新生儿败血症"[常用字段:智能] OR "新生儿脓毒血症"[常用字段:智能] OR "新生儿菌血症"[常用字段:智能] OR "新生儿脓毒败血症"[常用字段:智能]))))))))) 21 21:37:32**

**26 (((("白细胞介素类"[不加权:扩展]) OR ("白细胞介素10"[不加权:扩展]))) OR (("炎性细胞因子"[常用字段:智能] OR "促炎性细胞因子"[常用字段:智能] OR "促炎细胞因子"[常用字段:智能] OR "抗炎细胞因子"[常用字段:智能] OR "炎症因子"[常用字段:智能] OR "炎性因子"[常用字段:智能] OR "促炎因子"[常用字段:智能] OR "细胞因子"[常用字段:智能]))) 625216 21:35:48**

**25 ((((((("白细胞介素类"[不加权:扩展]) OR ("白细胞介素8"[不加权:扩展]))) OR (("炎性细胞因子"[常用字段:智能] OR "促炎性细胞因子"[常用字段:智能] OR "促炎细胞因子"[常用字段:智能] OR "抗炎细胞因子"[常用字段:智能] OR "炎症因子"[常用字段:智能] OR "炎性因子"[常用字段:智能] OR "促炎因子"[常用字段:智能] OR "细胞因子"[常用字段:智能]))))) AND ((((("多态性"[常用字段:智能] OR "基因多态性"[常用字段:智能] OR "遗传多态性"[常用字段:智能] OR "变异"[常用字段:智能] OR "突变"[常用字段:智能] OR "基因型"[常用字段:智能] OR "等位基因"[常用字段:智能] OR "DNA重复序列多态性"[常用字段:智能] OR "DNA片段长度多态性"[常用字段:智能])) AND ((((((((("婴儿, 新生"[不加权:扩展]) OR ("早产儿"[常用字段:智能] OR "足月儿"[常用字段:智能] OR "新生儿"[常用字段:智能] OR "新生婴儿"[常用字段:智能] OR "正常出生体重儿"[常用字段:智能] OR "低出生体重儿"[常用字段:智能] OR "巨大儿"[常用字段:智能] OR "极低出生体重儿"[常用字段:智能] OR "小于胎龄儿"[常用字段:智能]))) AND ((("菌血症"[常用字段:智能] OR "脓毒败血症"[常用字段:智能] OR "脓毒血症"[常用字段:智能]) OR ("脓毒症"[不加权:扩展] OR "出血性败血症"[不加权:扩展])))))) OR (("新生儿败血症"[常用字段:智能] OR "新生儿脓毒血症"[常用字段:智能] OR "新生儿菌血症"[常用字段:智能] OR "新生儿脓毒败血症"[常用字段:智能]))))))))) 21 21:34:32**

**24 (((("白细胞介素类"[不加权:扩展]) OR ("白细胞介素8"[不加权:扩展]))) OR (("炎性细胞因子"[常用字段:智能] OR "促炎性细胞因子"[常用字段:智能] OR "促炎细胞因子"[常用字段:智能] OR "抗炎细胞因子"[常用字段:智能] OR "炎症因子"[常用字段:智能] OR "炎性因子"[常用字段:智能] OR "促炎因子"[常用字段:智能] OR "细胞因子"[常用字段:智能]))) 625216 21:33:30**

**23 (((((((("炎性细胞因子"[常用字段:智能] OR "促炎性细胞因子"[常用字段:智能] OR "促炎细胞因子"[常用字段:智能] OR "抗炎细胞因子"[常用字段:智能] OR "炎症因子"[常用字段:智能] OR "炎性因子"[常用字段:智能] OR "促炎因子"[常用字段:智能] OR "细胞因子"[常用字段:智能])) OR ("白细胞介素类"[不加权:扩展]))) OR ("白细胞介素6"[不加权:扩展])))) AND ((((("多态性"[常用字段:智能] OR "基因多态性"[常用字段:智能] OR "遗传多态性"[常用字段:智能] OR "变异"[常用字段:智能] OR "突变"[常用字段:智能] OR "基因型"[常用字段:智能] OR "等位基因"[常用字段:智能] OR "DNA重复序列多态性"[常用字段:智能] OR "DNA片段长度多态性"[常用字段:智能])) AND ((((((((("婴儿, 新生"[不加权:扩展]) OR ("早产儿"[常用字段:智能] OR "足月儿"[常用字段:智能] OR "新生儿"[常用字段:智能] OR "新生婴儿"[常用字段:智能] OR "正常出生体重儿"[常用字段:智能] OR "低出生体重儿"[常用字段:智能] OR "巨大儿"[常用字段:智能] OR "极低出生体重儿"[常用字段:智能] OR "小于胎龄儿"[常用字段:智能]))) AND ((("菌血症"[常用字段:智能] OR "脓毒败血症"[常用字段:智能] OR "脓毒血症"[常用字段:智能]) OR ("脓毒症"[不加权:扩展] OR "出血性败血症"[不加权:扩展])))))) OR (("新生儿败血症"[常用字段:智能] OR "新生儿脓毒血症"[常用字段:智能] OR "新生儿菌血症"[常用字段:智能] OR "新生儿脓毒败血症"[常用字段:智能]))))))))) 21 21:29:54**

**22 ((((("炎性细胞因子"[常用字段:智能] OR "促炎性细胞因子"[常用字段:智能] OR "促炎细胞因子"[常用字段:智能] OR "抗炎细胞因子"[常用字段:智能] OR "炎症因子"[常用字段:智能] OR "炎性因子"[常用字段:智能] OR "促炎因子"[常用字段:智能] OR "细胞因子"[常用字段:智能])) OR ("白细胞介素类"[不加权:扩展]))) OR ("白细胞介素6"[不加权:扩展])) 625216 21:29:00**

**21 (((((((("炎性细胞因子"[常用字段:智能] OR "促炎性细胞因子"[常用字段:智能] OR "促炎细胞因子"[常用字段:智能] OR "抗炎细胞因子"[常用字段:智能] OR "炎症因子"[常用字段:智能] OR "炎性因子"[常用字段:智能] OR "促炎因子"[常用字段:智能] OR "细胞因子"[常用字段:智能])) OR ("白细胞介素类"[不加权:扩展]))) OR ("白细胞介素1"[不加权:扩展])))) AND ((((("多态性"[常用字段:智能] OR "基因多态性"[常用字段:智能] OR "遗传多态性"[常用字段:智能] OR "变异"[常用字段:智能] OR "突变"[常用字段:智能] OR "基因型"[常用字段:智能] OR "等位基因"[常用字段:智能] OR "DNA重复序列多态性"[常用字段:智能] OR "DNA片段长度多态性"[常用字段:智能])) AND ((((((((("婴儿, 新生"[不加权:扩展]) OR ("早产儿"[常用字段:智能] OR "足月儿"[常用字段:智能] OR "新生儿"[常用字段:智能] OR "新生婴儿"[常用字段:智能] OR "正常出生体重儿"[常用字段:智能] OR "低出生体重儿"[常用字段:智能] OR "巨大儿"[常用字段:智能] OR "极低出生体重儿"[常用字段:智能] OR "小于胎龄儿"[常用字段:智能]))) AND ((("菌血症"[常用字段:智能] OR "脓毒败血症"[常用字段:智能] OR "脓毒血症"[常用字段:智能]) OR ("脓毒症"[不加权:扩展] OR "出血性败血症"[不加权:扩展])))))) OR (("新生儿败血症"[常用字段:智能] OR "新生儿脓毒血症"[常用字段:智能] OR "新生儿菌血症"[常用字段:智能] OR "新生儿脓毒败血症"[常用字段:智能]))))))))) 21 21:25:20**

**20 ((((("炎性细胞因子"[常用字段:智能] OR "促炎性细胞因子"[常用字段:智能] OR "促炎细胞因子"[常用字段:智能] OR "抗炎细胞因子"[常用字段:智能] OR "炎症因子"[常用字段:智能] OR "炎性因子"[常用字段:智能] OR "促炎因子"[常用字段:智能] OR "细胞因子"[常用字段:智能])) OR ("白细胞介素类"[不加权:扩展]))) OR ("白细胞介素1"[不加权:扩展])) 625216 21:24:24**

**19 "炎性细胞因子"[常用字段:智能] OR "促炎性细胞因子"[常用字段:智能] OR "促炎细胞因子"[常用字段:智能] OR "抗炎细胞因子"[常用字段:智能] OR "炎症因子"[常用字段:智能] OR "炎性因子"[常用字段:智能] OR "促炎因子"[常用字段:智能] OR "细胞因子"[常用字段:智能] 342783 21:21:46**

**18 ((("多态性"[常用字段:智能] OR "基因多态性"[常用字段:智能] OR "遗传多态性"[常用字段:智能] OR "变异"[常用字段:智能] OR "突变"[常用字段:智能] OR "基因型"[常用字段:智能] OR "等位基因"[常用字段:智能] OR "DNA重复序列多态性"[常用字段:智能] OR "DNA片段长度多态性"[常用字段:智能])) AND ((((((((("婴儿, 新生"[不加权:扩展]) OR ("早产儿"[常用字段:智能] OR "足月儿"[常用字段:智能] OR "新生儿"[常用字段:智能] OR "新生婴儿"[常用字段:智能] OR "正常出生体重儿"[常用字段:智能] OR "低出生体重儿"[常用字段:智能] OR "巨大儿"[常用字段:智能] OR "极低出生体重儿"[常用字段:智能] OR "小于胎龄儿"[常用字段:智能]))) AND ((("菌血症"[常用字段:智能] OR "脓毒败血症"[常用字段:智能] OR "脓毒血症"[常用字段:智能]) OR ("脓毒症"[不加权:扩展] OR "出血性败血症"[不加权:扩展])))))) OR (("新生儿败血症"[常用字段:智能] OR "新生儿脓毒血症"[常用字段:智能] OR "新生儿菌血症"[常用字段:智能] OR "新生儿脓毒败血症"[常用字段:智能])))))) 434 21:19:16**

**17 ((((((("婴儿, 新生"[不加权:扩展]) OR ("早产儿"[常用字段:智能] OR "足月儿"[常用字段:智能] OR "新生儿"[常用字段:智能] OR "新生婴儿"[常用字段:智能] OR "正常出生体重儿"[常用字段:智能] OR "低出生体重儿"[常用字段:智能] OR "巨大儿"[常用字段:智能] OR "极低出生体重儿"[常用字段:智能] OR "小于胎龄儿"[常用字段:智能]))) AND ((("菌血症"[常用字段:智能] OR "脓毒败血症"[常用字段:智能] OR "脓毒血症"[常用字段:智能]) OR ("脓毒症"[不加权:扩展] OR "出血性败血症"[不加权:扩展])))))) OR (("新生儿败血症"[常用字段:智能] OR "新生儿脓毒血症"[常用字段:智能] OR "新生儿菌血症"[常用字段:智能] OR "新生儿脓毒败血症"[常用字段:智能]))) 18585 21:17:48**

**16 "新生儿败血症"[常用字段:智能] OR "新生儿脓毒血症"[常用字段:智能] OR "新生儿菌血症"[常用字段:智能] OR "新生儿脓毒败血症"[常用字段:智能] 2608 21:17:05**

**15 (((("婴儿, 新生"[不加权:扩展]) OR ("早产儿"[常用字段:智能] OR "足月儿"[常用字段:智能] OR "新生儿"[常用字段:智能] OR "新生婴儿"[常用字段:智能] OR "正常出生体重儿"[常用字段:智能] OR "低出生体重儿"[常用字段:智能] OR "巨大儿"[常用字段:智能] OR "极低出生体重儿"[常用字段:智能] OR "小于胎龄儿"[常用字段:智能]))) AND ((("菌血症"[常用字段:智能] OR "脓毒败血症"[常用字段:智能] OR "脓毒血症"[常用字段:智能]) OR ("脓毒症"[不加权:扩展] OR "出血性败血症"[不加权:扩展])))) 18119 21:15:04**

**14 ("菌血症"[常用字段:智能] OR "脓毒败血症"[常用字段:智能] OR "脓毒血症"[常用字段:智能]) OR ("脓毒症"[不加权:扩展] OR "出血性败血症"[不加权:扩展]) 257662 21:14:01**

**13 ("婴儿, 新生"[不加权:扩展]) OR ("早产儿"[常用字段:智能] OR "足月儿"[常用字段:智能] OR "新生儿"[常用字段:智能] OR "新生婴儿"[常用字段:智能] OR "正常出生体重儿"[常用字段:智能] OR "低出生体重儿"[常用字段:智能] OR "巨大儿"[常用字段:智能] OR "极低出生体重儿"[常用字段:智能] OR "小于胎龄儿"[常用字段:智能]) 699575 21:13:20**

**12 "新生儿脓毒症"[不加权:扩展] 231 21:12:31**

**11 "肿瘤坏死因子α"[不加权:扩展] OR "肿瘤坏死因子α"[不加权:扩展] OR "淋巴毒素α"[不加权:扩展] 197301 21:11:40**

**10 "白细胞介素10"[不加权:扩展] 36426 21:10:40**

**9 "白细胞介素6"[不加权:扩展] 24750 21:09:55**

**8 "白细胞介素8"[不加权:扩展] 29471 21:09:26**

**7 "白细胞介素1"[不加权:扩展] 81048 21:07:45**

**6 "白细胞介素类"[不加权:扩展] 344301 21:07:01**

**5 "多态性"[常用字段:智能] OR "基因多态性"[常用字段:智能] OR "遗传多态性"[常用字段:智能] OR "变异"[常用字段:智能] OR "突变"[常用字段:智能] OR "基因型"[常用字段:智能] OR "等位基因"[常用字段:智能] OR "DNA重复序列多态性"[常用字段:智能] OR "DNA片段长度多态性"[常用字段:智能] 1812030 21:05:26**

**4 "菌血症"[常用字段:智能] OR "脓毒败血症"[常用字段:智能] OR "脓毒血症"[常用字段:智能] 257662 21:02:39**

**3 "脓毒症"[不加权:扩展] OR "出血性败血症"[不加权:扩展] 170040 21:01:25**

**2 "婴儿, 新生"[不加权:扩展] 140882 21:00:18**

**1 "早产儿"[常用字段:智能] OR "足月儿"[常用字段:智能] OR "新生儿"[常用字段:智能] OR "新生婴儿"[常用字段:智能] OR "正常出生体重儿"[常用字段:智能] OR "低出生体重儿"[常用字段:智能] OR "巨大儿"[常用字段:智能] OR "极低出生体重儿"[常用字段:智能] OR "小于胎龄儿"[常用字段:智能] 699575 20:18:41**

PubMed：

IL-1(45):

Search: ****(((("Neonatal Sepsis"[Mesh]) OR (((((((((((((((((Neonatal Sepses[Title/Abstract]) OR (Sepses, Neonatal[Title/Abstract])) OR (Sepsis, Neonatal[Title/Abstract])) OR (Neonatal Late-Onset Sepsis[Title/Abstract])) OR (Late-Onset Sepses, Neonatal[Title/Abstract])) OR (Late-Onset Sepsis, Neonatal[Title/Abstract])) OR (Neonatal Late Onset Sepsis[Title/Abstract])) OR (Neonatal Late-Onset Sepses[Title/Abstract])) OR (Sepses, Neonatal Late-Onset[Title/Abstract])) OR (Sepsis, Neonatal Late-Onset[Title/Abstract])) OR (Neonatal Early-Onset Sepsis[Title/Abstract])) OR (Early-Onset Sepses, Neonatal[Title/Abstract])) OR (Early-Onset Sepsis, Neonatal[Title/Abstract])) OR (Neonatal Early Onset Sepsis[Title/Abstract])) OR (Neonatal Early-Onset Sepses[Title/Abstract])) OR (Sepses, Neonatal Early-Onset[Title/Abstract])) OR (Sepsis, Neonatal Early-Onset[Title/Abstract]))) OR (((((((((((((((((((((((("Infant, Newborn"[Mesh]) OR (((((((Infants, Newborn[Title/Abstract]) OR (Newborn Infant[Title/Abstract])) OR (Newborn Infants[Title/Abstract])) OR (Newborns[Title/Abstract])) OR (Newborn[Title/Abstract])) OR (Neonate[Title/Abstract])) OR (Neonates[Title/Abstract]))) OR ("Infant, Premature"[Mesh])) OR (((((((((Infants, Premature[Title/Abstract]) OR (Premature Infant[Title/Abstract])) OR (Preterm Infants[Title/Abstract])) OR (Infant, Preterm[Title/Abstract])) OR (Infants, Preterm[Title/Abstract])) OR (Preterm Infant[Title/Abstract])) OR (Premature Infants[Title/Abstract])) OR (Neonatal Prematurity[Title/Abstract])) OR (Prematurity, Neonatal[Title/Abstract]))) OR ("Infant, Extremely Premature"[Mesh])) OR (((((((((((Extremely Premature Infant[Title/Abstract]) OR (Infants, Extremely Premature[Title/Abstract])) OR (Premature Infant, Extremely[Title/Abstract])) OR (Premature Infants, Extremely[Title/Abstract])) OR (Extremely Preterm Infants[Title/Abstract])) OR (Extremely Preterm Infant[Title/Abstract])) OR (Infant, Extremely Preterm[Title/Abstract])) OR (Infants, Extremely Preterm[Title/Abstract])) OR (Preterm Infant, Extremely[Title/Abstract])) OR (Preterm Infants, Extremely[Title/Abstract])) OR (Extremely Premature Infants[Title/Abstract]))) OR ("Term Birth"[Mesh])) OR (((((((((((Birth, Term[Title/Abstract]) OR (Term Births[Title/Abstract])) OR (Birth of Full-Term Newborn[Title/Abstract])) OR (Birth of Full Term Newborn[Title/Abstract])) OR (Fullterm Birth[Title/Abstract])) OR (Birth, Fullterm[Title/Abstract])) OR (Births, Fullterm[Title/Abstract])) OR (Fullterm Births[Title/Abstract])) OR (Birth of Full-Term Infant[Title/Abstract])) OR (Birth of Full Term Infant[Title/Abstract])) OR (Full-Term Infant Births[Title/Abstract]))) OR (Overdue birth)) OR (Normal birth weight infants)) OR ("Infant, Low Birth Weight"[Mesh])) OR (((((((((Low-Birth-Weight Infant[Title/Abstract]) OR (Infant, Low-Birth-Weight[Title/Abstract])) OR (Infants, Low-Birth-Weight[Title/Abstract])) OR (Low Birth Weight Infant[Title/Abstract])) OR (Low-Birth-Weight Infants[Title/Abstract])) OR (Low Birth Weight[Title/Abstract])) OR (Birth Weight, Low[Title/Abstract])) OR (Birth Weights, Low[Title/Abstract])) OR (Low Birth Weights[Title/Abstract]))) OR ("Infant, Extremely Low Birth Weight"[Mesh])) OR (Extremely Low Birth Weight Infant[Title/Abstract])) OR ("Infant, Very Low Birth Weight"[Mesh])) OR ((((((Very-Low-Birth-Weight Infant[Title/Abstract]) OR (Infant, Very-Low-Birth-Weight[Title/Abstract])) OR (Infants, Very-Low-Birth-Weight[Title/Abstract])) OR (Very Low Birth Weight Infant[Title/Abstract])) OR (Very-Low-Birth-Weight Infants[Title/Abstract])) OR (Very Low Birth Weight[Title/Abstract]))) OR (Giant)) OR (Suitable for gestational age)) OR (Large for gestational age)) OR (Early newborn)) OR (Late newborn)) OR (High risk children)) OR ("Infant, Small for Gestational Age"[Mesh])) AND (((("Sepsis"[Mesh]) OR (((((((((((((((((Bloodstream Infection[Title/Abstract]) OR (Bloodstream Infections[Title/Abstract])) OR (Infection, Bloodstream[Title/Abstract])) OR (Pyemia[Title/Abstract])) OR (Pyemias[Title/Abstract])) OR (Pyohemia[Title/Abstract])) OR (Pyohemias[Title/Abstract])) OR (Pyaemia[Title/Abstract])) OR (Pyaemias[Title/Abstract])) OR (Septicemia[Title/Abstract])) OR (Septicemias[Title/Abstract])) OR (Poisoning, Blood[Title/Abstract])) OR (Blood Poisoning[Title/Abstract])) OR (Blood Poisonings[Title/Abstract])) OR (Poisonings, Blood[Title/Abstract])) OR (Severe Sepsis[Title/Abstract])) OR (Sepsis, Severe[Title/Abstract]))) OR ("Bacteremia"[Mesh])) OR (Bacteremias[Title/Abstract])))) AND (("Polymorphism, Genetic"[Mesh]) OR (((((((((Polymorphisms, Genetic[Title/Abstract]) OR (Genetic Polymorphism[Title/Abstract])) OR (Genetic Polymorphisms[Title/Abstract])) OR (Gene Polymorphism[Title/Abstract])) OR (Gene Polymorphisms[Title/Abstract])) OR (Polymorphism, Gene[Title/Abstract])) OR (Polymorphisms, Gene[Title/Abstract])) OR (Polymorphism (Genetics[Title/Abstract]))) OR (Polymorphisms (Genetics[Title/Abstract]))))) AND ((((((((("Cytokines"[Mesh]) OR (Cytokine[Title/Abstract])) OR (Inflammatory cytokines)) OR (Proinflammatory cytokines)) OR (anti-inflammatory cytokine)) OR (Inflammatory factor)) OR (Proinflammatory factor))) OR (("Interleukins"[Mesh]) OR ((((Interleukin[Title/Abstract]) OR (Interleukin-1[Title/Abstract])) OR (Interleukin-1alpha[Title/Abstract])) OR (Interleukin-1beta[Title/Abstract]))))****

IL-6(45):

Search: ****(((("Neonatal Sepsis"[Mesh]) OR (((((((((((((((((Neonatal Sepses[Title/Abstract]) OR (Sepses, Neonatal[Title/Abstract])) OR (Sepsis, Neonatal[Title/Abstract])) OR (Neonatal Late-Onset Sepsis[Title/Abstract])) OR (Late-Onset Sepses, Neonatal[Title/Abstract])) OR (Late-Onset Sepsis, Neonatal[Title/Abstract])) OR (Neonatal Late Onset Sepsis[Title/Abstract])) OR (Neonatal Late-Onset Sepses[Title/Abstract])) OR (Sepses, Neonatal Late-Onset[Title/Abstract])) OR (Sepsis, Neonatal Late-Onset[Title/Abstract])) OR (Neonatal Early-Onset Sepsis[Title/Abstract])) OR (Early-Onset Sepses, Neonatal[Title/Abstract])) OR (Early-Onset Sepsis, Neonatal[Title/Abstract])) OR (Neonatal Early Onset Sepsis[Title/Abstract])) OR (Neonatal Early-Onset Sepses[Title/Abstract])) OR (Sepses, Neonatal Early-Onset[Title/Abstract])) OR (Sepsis, Neonatal Early-Onset[Title/Abstract]))) OR (((((((((((((((((((((((("Infant, Newborn"[Mesh]) OR (((((((Infants, Newborn[Title/Abstract]) OR (Newborn Infant[Title/Abstract])) OR (Newborn Infants[Title/Abstract])) OR (Newborns[Title/Abstract])) OR (Newborn[Title/Abstract])) OR (Neonate[Title/Abstract])) OR (Neonates[Title/Abstract]))) OR ("Infant, Premature"[Mesh])) OR (((((((((Infants, Premature[Title/Abstract]) OR (Premature Infant[Title/Abstract])) OR (Preterm Infants[Title/Abstract])) OR (Infant, Preterm[Title/Abstract])) OR (Infants, Preterm[Title/Abstract])) OR (Preterm Infant[Title/Abstract])) OR (Premature Infants[Title/Abstract])) OR (Neonatal Prematurity[Title/Abstract])) OR (Prematurity, Neonatal[Title/Abstract]))) OR ("Infant, Extremely Premature"[Mesh])) OR (((((((((((Extremely Premature Infant[Title/Abstract]) OR (Infants, Extremely Premature[Title/Abstract])) OR (Premature Infant, Extremely[Title/Abstract])) OR (Premature Infants, Extremely[Title/Abstract])) OR (Extremely Preterm Infants[Title/Abstract])) OR (Extremely Preterm Infant[Title/Abstract])) OR (Infant, Extremely Preterm[Title/Abstract])) OR (Infants, Extremely Preterm[Title/Abstract])) OR (Preterm Infant, Extremely[Title/Abstract])) OR (Preterm Infants, Extremely[Title/Abstract])) OR (Extremely Premature Infants[Title/Abstract]))) OR ("Term Birth"[Mesh])) OR (((((((((((Birth, Term[Title/Abstract]) OR (Term Births[Title/Abstract])) OR (Birth of Full-Term Newborn[Title/Abstract])) OR (Birth of Full Term Newborn[Title/Abstract])) OR (Fullterm Birth[Title/Abstract])) OR (Birth, Fullterm[Title/Abstract])) OR (Births, Fullterm[Title/Abstract])) OR (Fullterm Births[Title/Abstract])) OR (Birth of Full-Term Infant[Title/Abstract])) OR (Birth of Full Term Infant[Title/Abstract])) OR (Full-Term Infant Births[Title/Abstract]))) OR (Overdue birth)) OR (Normal birth weight infants)) OR ("Infant, Low Birth Weight"[Mesh])) OR (((((((((Low-Birth-Weight Infant[Title/Abstract]) OR (Infant, Low-Birth-Weight[Title/Abstract])) OR (Infants, Low-Birth-Weight[Title/Abstract])) OR (Low Birth Weight Infant[Title/Abstract])) OR (Low-Birth-Weight Infants[Title/Abstract])) OR (Low Birth Weight[Title/Abstract])) OR (Birth Weight, Low[Title/Abstract])) OR (Birth Weights, Low[Title/Abstract])) OR (Low Birth Weights[Title/Abstract]))) OR ("Infant, Extremely Low Birth Weight"[Mesh])) OR (Extremely Low Birth Weight Infant[Title/Abstract])) OR ("Infant, Very Low Birth Weight"[Mesh])) OR ((((((Very-Low-Birth-Weight Infant[Title/Abstract]) OR (Infant, Very-Low-Birth-Weight[Title/Abstract])) OR (Infants, Very-Low-Birth-Weight[Title/Abstract])) OR (Very Low Birth Weight Infant[Title/Abstract])) OR (Very-Low-Birth-Weight Infants[Title/Abstract])) OR (Very Low Birth Weight[Title/Abstract]))) OR (Giant)) OR (Suitable for gestational age)) OR (Large for gestational age)) OR (Early newborn)) OR (Late newborn)) OR (High risk children)) OR ("Infant, Small for Gestational Age"[Mesh])) AND (((("Sepsis"[Mesh]) OR (((((((((((((((((Bloodstream Infection[Title/Abstract]) OR (Bloodstream Infections[Title/Abstract])) OR (Infection, Bloodstream[Title/Abstract])) OR (Pyemia[Title/Abstract])) OR (Pyemias[Title/Abstract])) OR (Pyohemia[Title/Abstract])) OR (Pyohemias[Title/Abstract])) OR (Pyaemia[Title/Abstract])) OR (Pyaemias[Title/Abstract])) OR (Septicemia[Title/Abstract])) OR (Septicemias[Title/Abstract])) OR (Poisoning, Blood[Title/Abstract])) OR (Blood Poisoning[Title/Abstract])) OR (Blood Poisonings[Title/Abstract])) OR (Poisonings, Blood[Title/Abstract])) OR (Severe Sepsis[Title/Abstract])) OR (Sepsis, Severe[Title/Abstract]))) OR ("Bacteremia"[Mesh])) OR (Bacteremias[Title/Abstract])))) AND (("Polymorphism, Genetic"[Mesh]) OR (((((((((Polymorphisms, Genetic[Title/Abstract]) OR (Genetic Polymorphism[Title/Abstract])) OR (Genetic Polymorphisms[Title/Abstract])) OR (Gene Polymorphism[Title/Abstract])) OR (Gene Polymorphisms[Title/Abstract])) OR (Polymorphism, Gene[Title/Abstract])) OR (Polymorphisms, Gene[Title/Abstract])) OR (Polymorphism (Genetics[Title/Abstract]))) OR (Polymorphisms (Genetics[Title/Abstract]))))) AND ((((((((("Cytokines"[Mesh]) OR (Cytokine[Title/Abstract])) OR (Inflammatory cytokines)) OR (Proinflammatory cytokines)) OR (anti-inflammatory cytokine)) OR (Inflammatory factor)) OR (Proinflammatory factor))) OR (("Interleukins"[Mesh]) OR ((Interleukin[Title/Abstract]) OR (Interleukin-6[Title/Abstract]))))****

IL-8(45）:

Search: ****(((("Neonatal Sepsis"[Mesh]) OR (((((((((((((((((Neonatal Sepses[Title/Abstract]) OR (Sepses, Neonatal[Title/Abstract])) OR (Sepsis, Neonatal[Title/Abstract])) OR (Neonatal Late-Onset Sepsis[Title/Abstract])) OR (Late-Onset Sepses, Neonatal[Title/Abstract])) OR (Late-Onset Sepsis, Neonatal[Title/Abstract])) OR (Neonatal Late Onset Sepsis[Title/Abstract])) OR (Neonatal Late-Onset Sepses[Title/Abstract])) OR (Sepses, Neonatal Late-Onset[Title/Abstract])) OR (Sepsis, Neonatal Late-Onset[Title/Abstract])) OR (Neonatal Early-Onset Sepsis[Title/Abstract])) OR (Early-Onset Sepses, Neonatal[Title/Abstract])) OR (Early-Onset Sepsis, Neonatal[Title/Abstract])) OR (Neonatal Early Onset Sepsis[Title/Abstract])) OR (Neonatal Early-Onset Sepses[Title/Abstract])) OR (Sepses, Neonatal Early-Onset[Title/Abstract])) OR (Sepsis, Neonatal Early-Onset[Title/Abstract]))) OR (((((((((((((((((((((((("Infant, Newborn"[Mesh]) OR (((((((Infants, Newborn[Title/Abstract]) OR (Newborn Infant[Title/Abstract])) OR (Newborn Infants[Title/Abstract])) OR (Newborns[Title/Abstract])) OR (Newborn[Title/Abstract])) OR (Neonate[Title/Abstract])) OR (Neonates[Title/Abstract]))) OR ("Infant, Premature"[Mesh])) OR (((((((((Infants, Premature[Title/Abstract]) OR (Premature Infant[Title/Abstract])) OR (Preterm Infants[Title/Abstract])) OR (Infant, Preterm[Title/Abstract])) OR (Infants, Preterm[Title/Abstract])) OR (Preterm Infant[Title/Abstract])) OR (Premature Infants[Title/Abstract])) OR (Neonatal Prematurity[Title/Abstract])) OR (Prematurity, Neonatal[Title/Abstract]))) OR ("Infant, Extremely Premature"[Mesh])) OR (((((((((((Extremely Premature Infant[Title/Abstract]) OR (Infants, Extremely Premature[Title/Abstract])) OR (Premature Infant, Extremely[Title/Abstract])) OR (Premature Infants, Extremely[Title/Abstract])) OR (Extremely Preterm Infants[Title/Abstract])) OR (Extremely Preterm Infant[Title/Abstract])) OR (Infant, Extremely Preterm[Title/Abstract])) OR (Infants, Extremely Preterm[Title/Abstract])) OR (Preterm Infant, Extremely[Title/Abstract])) OR (Preterm Infants, Extremely[Title/Abstract])) OR (Extremely Premature Infants[Title/Abstract]))) OR ("Term Birth"[Mesh])) OR (((((((((((Birth, Term[Title/Abstract]) OR (Term Births[Title/Abstract])) OR (Birth of Full-Term Newborn[Title/Abstract])) OR (Birth of Full Term Newborn[Title/Abstract])) OR (Fullterm Birth[Title/Abstract])) OR (Birth, Fullterm[Title/Abstract])) OR (Births, Fullterm[Title/Abstract])) OR (Fullterm Births[Title/Abstract])) OR (Birth of Full-Term Infant[Title/Abstract])) OR (Birth of Full Term Infant[Title/Abstract])) OR (Full-Term Infant Births[Title/Abstract]))) OR (Overdue birth)) OR (Normal birth weight infants)) OR ("Infant, Low Birth Weight"[Mesh])) OR (((((((((Low-Birth-Weight Infant[Title/Abstract]) OR (Infant, Low-Birth-Weight[Title/Abstract])) OR (Infants, Low-Birth-Weight[Title/Abstract])) OR (Low Birth Weight Infant[Title/Abstract])) OR (Low-Birth-Weight Infants[Title/Abstract])) OR (Low Birth Weight[Title/Abstract])) OR (Birth Weight, Low[Title/Abstract])) OR (Birth Weights, Low[Title/Abstract])) OR (Low Birth Weights[Title/Abstract]))) OR ("Infant, Extremely Low Birth Weight"[Mesh])) OR (Extremely Low Birth Weight Infant[Title/Abstract])) OR ("Infant, Very Low Birth Weight"[Mesh])) OR ((((((Very-Low-Birth-Weight Infant[Title/Abstract]) OR (Infant, Very-Low-Birth-Weight[Title/Abstract])) OR (Infants, Very-Low-Birth-Weight[Title/Abstract])) OR (Very Low Birth Weight Infant[Title/Abstract])) OR (Very-Low-Birth-Weight Infants[Title/Abstract])) OR (Very Low Birth Weight[Title/Abstract]))) OR (Giant)) OR (Suitable for gestational age)) OR (Large for gestational age)) OR (Early newborn)) OR (Late newborn)) OR (High risk children)) OR ("Infant, Small for Gestational Age"[Mesh])) AND (((("Sepsis"[Mesh]) OR (((((((((((((((((Bloodstream Infection[Title/Abstract]) OR (Bloodstream Infections[Title/Abstract])) OR (Infection, Bloodstream[Title/Abstract])) OR (Pyemia[Title/Abstract])) OR (Pyemias[Title/Abstract])) OR (Pyohemia[Title/Abstract])) OR (Pyohemias[Title/Abstract])) OR (Pyaemia[Title/Abstract])) OR (Pyaemias[Title/Abstract])) OR (Septicemia[Title/Abstract])) OR (Septicemias[Title/Abstract])) OR (Poisoning, Blood[Title/Abstract])) OR (Blood Poisoning[Title/Abstract])) OR (Blood Poisonings[Title/Abstract])) OR (Poisonings, Blood[Title/Abstract])) OR (Severe Sepsis[Title/Abstract])) OR (Sepsis, Severe[Title/Abstract]))) OR ("Bacteremia"[Mesh])) OR (Bacteremias[Title/Abstract])))) AND (("Polymorphism, Genetic"[Mesh]) OR (((((((((Polymorphisms, Genetic[Title/Abstract]) OR (Genetic Polymorphism[Title/Abstract])) OR (Genetic Polymorphisms[Title/Abstract])) OR (Gene Polymorphism[Title/Abstract])) OR (Gene Polymorphisms[Title/Abstract])) OR (Polymorphism, Gene[Title/Abstract])) OR (Polymorphisms, Gene[Title/Abstract])) OR (Polymorphism (Genetics[Title/Abstract]))) OR (Polymorphisms (Genetics[Title/Abstract]))))) AND ((((((((("Cytokines"[Mesh]) OR (Cytokine[Title/Abstract])) OR (Inflammatory cytokines)) OR (Proinflammatory cytokines)) OR (anti-inflammatory cytokine)) OR (Inflammatory factor)) OR (Proinflammatory factor))) OR (("Interleukins"[Mesh]) OR ((Interleukin[Title/Abstract]) OR (Interleukin-8[Title/Abstract]))))****

IL-10(45):

Search: ****(((("Neonatal Sepsis"[Mesh]) OR (((((((((((((((((Neonatal Sepses[Title/Abstract]) OR (Sepses, Neonatal[Title/Abstract])) OR (Sepsis, Neonatal[Title/Abstract])) OR (Neonatal Late-Onset Sepsis[Title/Abstract])) OR (Late-Onset Sepses, Neonatal[Title/Abstract])) OR (Late-Onset Sepsis, Neonatal[Title/Abstract])) OR (Neonatal Late Onset Sepsis[Title/Abstract])) OR (Neonatal Late-Onset Sepses[Title/Abstract])) OR (Sepses, Neonatal Late-Onset[Title/Abstract])) OR (Sepsis, Neonatal Late-Onset[Title/Abstract])) OR (Neonatal Early-Onset Sepsis[Title/Abstract])) OR (Early-Onset Sepses, Neonatal[Title/Abstract])) OR (Early-Onset Sepsis, Neonatal[Title/Abstract])) OR (Neonatal Early Onset Sepsis[Title/Abstract])) OR (Neonatal Early-Onset Sepses[Title/Abstract])) OR (Sepses, Neonatal Early-Onset[Title/Abstract])) OR (Sepsis, Neonatal Early-Onset[Title/Abstract]))) OR (((((((((((((((((((((((("Infant, Newborn"[Mesh]) OR (((((((Infants, Newborn[Title/Abstract]) OR (Newborn Infant[Title/Abstract])) OR (Newborn Infants[Title/Abstract])) OR (Newborns[Title/Abstract])) OR (Newborn[Title/Abstract])) OR (Neonate[Title/Abstract])) OR (Neonates[Title/Abstract]))) OR ("Infant, Premature"[Mesh])) OR (((((((((Infants, Premature[Title/Abstract]) OR (Premature Infant[Title/Abstract])) OR (Preterm Infants[Title/Abstract])) OR (Infant, Preterm[Title/Abstract])) OR (Infants, Preterm[Title/Abstract])) OR (Preterm Infant[Title/Abstract])) OR (Premature Infants[Title/Abstract])) OR (Neonatal Prematurity[Title/Abstract])) OR (Prematurity, Neonatal[Title/Abstract]))) OR ("Infant, Extremely Premature"[Mesh])) OR (((((((((((Extremely Premature Infant[Title/Abstract]) OR (Infants, Extremely Premature[Title/Abstract])) OR (Premature Infant, Extremely[Title/Abstract])) OR (Premature Infants, Extremely[Title/Abstract])) OR (Extremely Preterm Infants[Title/Abstract])) OR (Extremely Preterm Infant[Title/Abstract])) OR (Infant, Extremely Preterm[Title/Abstract])) OR (Infants, Extremely Preterm[Title/Abstract])) OR (Preterm Infant, Extremely[Title/Abstract])) OR (Preterm Infants, Extremely[Title/Abstract])) OR (Extremely Premature Infants[Title/Abstract]))) OR ("Term Birth"[Mesh])) OR (((((((((((Birth, Term[Title/Abstract]) OR (Term Births[Title/Abstract])) OR (Birth of Full-Term Newborn[Title/Abstract])) OR (Birth of Full Term Newborn[Title/Abstract])) OR (Fullterm Birth[Title/Abstract])) OR (Birth, Fullterm[Title/Abstract])) OR (Births, Fullterm[Title/Abstract])) OR (Fullterm Births[Title/Abstract])) OR (Birth of Full-Term Infant[Title/Abstract])) OR (Birth of Full Term Infant[Title/Abstract])) OR (Full-Term Infant Births[Title/Abstract]))) OR (Overdue birth)) OR (Normal birth weight infants)) OR ("Infant, Low Birth Weight"[Mesh])) OR (((((((((Low-Birth-Weight Infant[Title/Abstract]) OR (Infant, Low-Birth-Weight[Title/Abstract])) OR (Infants, Low-Birth-Weight[Title/Abstract])) OR (Low Birth Weight Infant[Title/Abstract])) OR (Low-Birth-Weight Infants[Title/Abstract])) OR (Low Birth Weight[Title/Abstract])) OR (Birth Weight, Low[Title/Abstract])) OR (Birth Weights, Low[Title/Abstract])) OR (Low Birth Weights[Title/Abstract]))) OR ("Infant, Extremely Low Birth Weight"[Mesh])) OR (Extremely Low Birth Weight Infant[Title/Abstract])) OR ("Infant, Very Low Birth Weight"[Mesh])) OR ((((((Very-Low-Birth-Weight Infant[Title/Abstract]) OR (Infant, Very-Low-Birth-Weight[Title/Abstract])) OR (Infants, Very-Low-Birth-Weight[Title/Abstract])) OR (Very Low Birth Weight Infant[Title/Abstract])) OR (Very-Low-Birth-Weight Infants[Title/Abstract])) OR (Very Low Birth Weight[Title/Abstract]))) OR (Giant)) OR (Suitable for gestational age)) OR (Large for gestational age)) OR (Early newborn)) OR (Late newborn)) OR (High risk children)) OR ("Infant, Small for Gestational Age"[Mesh])) AND (((("Sepsis"[Mesh]) OR (((((((((((((((((Bloodstream Infection[Title/Abstract]) OR (Bloodstream Infections[Title/Abstract])) OR (Infection, Bloodstream[Title/Abstract])) OR (Pyemia[Title/Abstract])) OR (Pyemias[Title/Abstract])) OR (Pyohemia[Title/Abstract])) OR (Pyohemias[Title/Abstract])) OR (Pyaemia[Title/Abstract])) OR (Pyaemias[Title/Abstract])) OR (Septicemia[Title/Abstract])) OR (Septicemias[Title/Abstract])) OR (Poisoning, Blood[Title/Abstract])) OR (Blood Poisoning[Title/Abstract])) OR (Blood Poisonings[Title/Abstract])) OR (Poisonings, Blood[Title/Abstract])) OR (Severe Sepsis[Title/Abstract])) OR (Sepsis, Severe[Title/Abstract]))) OR ("Bacteremia"[Mesh])) OR (Bacteremias[Title/Abstract])))) AND (("Polymorphism, Genetic"[Mesh]) OR (((((((((Polymorphisms, Genetic[Title/Abstract]) OR (Genetic Polymorphism[Title/Abstract])) OR (Genetic Polymorphisms[Title/Abstract])) OR (Gene Polymorphism[Title/Abstract])) OR (Gene Polymorphisms[Title/Abstract])) OR (Polymorphism, Gene[Title/Abstract])) OR (Polymorphisms, Gene[Title/Abstract])) OR (Polymorphism (Genetics[Title/Abstract]))) OR (Polymorphisms (Genetics[Title/Abstract]))))) AND ((((((((("Cytokines"[Mesh]) OR (Cytokine[Title/Abstract])) OR (Inflammatory cytokines)) OR (Proinflammatory cytokines)) OR (anti-inflammatory cytokine)) OR (Inflammatory factor)) OR (Proinflammatory factor))) OR (("Interleukins"[Mesh]) OR ((Interleukin[Title/Abstract]) OR (Interleukin-10[Title/Abstract]))))****

TNF-α(42):

Search: ****(((("Neonatal Sepsis"[Mesh]) OR (((((((((((((((((Neonatal Sepses[Title/Abstract]) OR (Sepses, Neonatal[Title/Abstract])) OR (Sepsis, Neonatal[Title/Abstract])) OR (Neonatal Late-Onset Sepsis[Title/Abstract])) OR (Late-Onset Sepses, Neonatal[Title/Abstract])) OR (Late-Onset Sepsis, Neonatal[Title/Abstract])) OR (Neonatal Late Onset Sepsis[Title/Abstract])) OR (Neonatal Late-Onset Sepses[Title/Abstract])) OR (Sepses, Neonatal Late-Onset[Title/Abstract])) OR (Sepsis, Neonatal Late-Onset[Title/Abstract])) OR (Neonatal Early-Onset Sepsis[Title/Abstract])) OR (Early-Onset Sepses, Neonatal[Title/Abstract])) OR (Early-Onset Sepsis, Neonatal[Title/Abstract])) OR (Neonatal Early Onset Sepsis[Title/Abstract])) OR (Neonatal Early-Onset Sepses[Title/Abstract])) OR (Sepses, Neonatal Early-Onset[Title/Abstract])) OR (Sepsis, Neonatal Early-Onset[Title/Abstract]))) OR (((((((((((((((((((((((("Infant, Newborn"[Mesh]) OR (((((((Infants, Newborn[Title/Abstract]) OR (Newborn Infant[Title/Abstract])) OR (Newborn Infants[Title/Abstract])) OR (Newborns[Title/Abstract])) OR (Newborn[Title/Abstract])) OR (Neonate[Title/Abstract])) OR (Neonates[Title/Abstract]))) OR ("Infant, Premature"[Mesh])) OR (((((((((Infants, Premature[Title/Abstract]) OR (Premature Infant[Title/Abstract])) OR (Preterm Infants[Title/Abstract])) OR (Infant, Preterm[Title/Abstract])) OR (Infants, Preterm[Title/Abstract])) OR (Preterm Infant[Title/Abstract])) OR (Premature Infants[Title/Abstract])) OR (Neonatal Prematurity[Title/Abstract])) OR (Prematurity, Neonatal[Title/Abstract]))) OR ("Infant, Extremely Premature"[Mesh])) OR (((((((((((Extremely Premature Infant[Title/Abstract]) OR (Infants, Extremely Premature[Title/Abstract])) OR (Premature Infant, Extremely[Title/Abstract])) OR (Premature Infants, Extremely[Title/Abstract])) OR (Extremely Preterm Infants[Title/Abstract])) OR (Extremely Preterm Infant[Title/Abstract])) OR (Infant, Extremely Preterm[Title/Abstract])) OR (Infants, Extremely Preterm[Title/Abstract])) OR (Preterm Infant, Extremely[Title/Abstract])) OR (Preterm Infants, Extremely[Title/Abstract])) OR (Extremely Premature Infants[Title/Abstract]))) OR ("Term Birth"[Mesh])) OR (((((((((((Birth, Term[Title/Abstract]) OR (Term Births[Title/Abstract])) OR (Birth of Full-Term Newborn[Title/Abstract])) OR (Birth of Full Term Newborn[Title/Abstract])) OR (Fullterm Birth[Title/Abstract])) OR (Birth, Fullterm[Title/Abstract])) OR (Births, Fullterm[Title/Abstract])) OR (Fullterm Births[Title/Abstract])) OR (Birth of Full-Term Infant[Title/Abstract])) OR (Birth of Full Term Infant[Title/Abstract])) OR (Full-Term Infant Births[Title/Abstract]))) OR (Overdue birth)) OR (Normal birth weight infants)) OR ("Infant, Low Birth Weight"[Mesh])) OR (((((((((Low-Birth-Weight Infant[Title/Abstract]) OR (Infant, Low-Birth-Weight[Title/Abstract])) OR (Infants, Low-Birth-Weight[Title/Abstract])) OR (Low Birth Weight Infant[Title/Abstract])) OR (Low-Birth-Weight Infants[Title/Abstract])) OR (Low Birth Weight[Title/Abstract])) OR (Birth Weight, Low[Title/Abstract])) OR (Birth Weights, Low[Title/Abstract])) OR (Low Birth Weights[Title/Abstract]))) OR ("Infant, Extremely Low Birth Weight"[Mesh])) OR (Extremely Low Birth Weight Infant[Title/Abstract])) OR ("Infant, Very Low Birth Weight"[Mesh])) OR ((((((Very-Low-Birth-Weight Infant[Title/Abstract]) OR (Infant, Very-Low-Birth-Weight[Title/Abstract])) OR (Infants, Very-Low-Birth-Weight[Title/Abstract])) OR (Very Low Birth Weight Infant[Title/Abstract])) OR (Very-Low-Birth-Weight Infants[Title/Abstract])) OR (Very Low Birth Weight[Title/Abstract]))) OR (Giant)) OR (Suitable for gestational age)) OR (Large for gestational age)) OR (Early newborn)) OR (Late newborn)) OR (High risk children)) OR ("Infant, Small for Gestational Age"[Mesh])) AND (((("Sepsis"[Mesh]) OR (((((((((((((((((Bloodstream Infection[Title/Abstract]) OR (Bloodstream Infections[Title/Abstract])) OR (Infection, Bloodstream[Title/Abstract])) OR (Pyemia[Title/Abstract])) OR (Pyemias[Title/Abstract])) OR (Pyohemia[Title/Abstract])) OR (Pyohemias[Title/Abstract])) OR (Pyaemia[Title/Abstract])) OR (Pyaemias[Title/Abstract])) OR (Septicemia[Title/Abstract])) OR (Septicemias[Title/Abstract])) OR (Poisoning, Blood[Title/Abstract])) OR (Blood Poisoning[Title/Abstract])) OR (Blood Poisonings[Title/Abstract])) OR (Poisonings, Blood[Title/Abstract])) OR (Severe Sepsis[Title/Abstract])) OR (Sepsis, Severe[Title/Abstract]))) OR ("Bacteremia"[Mesh])) OR (Bacteremias[Title/Abstract])))) AND (("Polymorphism, Genetic"[Mesh]) OR (((((((((Polymorphisms, Genetic[Title/Abstract]) OR (Genetic Polymorphism[Title/Abstract])) OR (Genetic Polymorphisms[Title/Abstract])) OR (Gene Polymorphism[Title/Abstract])) OR (Gene Polymorphisms[Title/Abstract])) OR (Polymorphism, Gene[Title/Abstract])) OR (Polymorphisms, Gene[Title/Abstract])) OR (Polymorphism (Genetics[Title/Abstract]))) OR (Polymorphisms (Genetics[Title/Abstract]))))) AND ((((((((("Cytokines"[Mesh]) OR (Cytokine[Title/Abstract])) OR (Inflammatory cytokines)) OR (Proinflammatory cytokines)) OR (anti-inflammatory cytokine)) OR (Inflammatory factor)) OR (Proinflammatory factor))) OR (("Tumor Necrosis Factor-alpha"[Mesh]) OR (((((((((Tumor Necrosis Factor alpha[Title/Abstract]) OR (Cachectin[Title/Abstract])) OR (Cachectin-Tumor Necrosis Factor[Title/Abstract])) OR (Cachectin Tumor Necrosis Factor[Title/Abstract])) OR (Tumor Necrosis Factor Ligand Superfamily Member 2[Title/Abstract])) OR (Tumor Necrosis Factor[Title/Abstract])) OR (TNF Superfamily, Member 2[Title/Abstract])) OR (TNFalpha[Title/Abstract])) OR (TNF-alpha[Title/Abstract]))))****

Embase：

IL-1（12）

IL-6（12）

IL-8（12）

IL-10（12）

TNF-α（13）

| No. | Query | Results |
| --- | --- | --- |
| #34 | #27 AND #29 | 13 |
| #33 | #26 AND #29 | 12 |
| #32 | #25 AND #29 | 12 |
| #31 | #24 AND #29 | 12 |
| #30 | #23 AND #29 | 12 |
| #29 | #16 AND #28 | 56 |
| #28 | 'polymorphism, genetic':ab,ti OR 'polymorphisms, genetic':ab,ti OR 'genetic polymorphism':ab,ti OR 'genetic polymorphisms':ab,ti OR 'gene polymorphism':ab,ti OR 'gene polymorphisms':ab,ti OR 'polymorphism, gene':ab,ti OR 'polymorphisms, gene':ab,ti OR 'polymorphism (genetics)':ab,ti OR 'polymorphisms (genetics)':ab,ti | 80358 |
| #27 | #17 OR #22 | 586843 |
| #26 | #17 OR #21 | 684388 |
| #25 | #17 OR #20 | 684388 |
| #24 | #17 OR #19 | 684392 |
| #23 | #17 OR #18 | 684388 |
| #22 | 'tumor necrosis factor-alpha':ab,ti OR 'tumor necrosis factor alpha':ab,ti OR 'cachectin':ab,ti OR 'cachectin-tumor necrosis factor':ab,ti OR 'cachectin tumor necrosis factor':ab,ti OR 'tumor necrosis factor ligand superfamily member 2':ab,ti OR 'tumor necrosis factor':ab,ti OR 'tnf superfamily, member 2':ab,ti OR 'tnfalpha':ab,ti OR 'tnf-alpha':ab,ti | 188050 |
| #21 | 'interleukin':ab,ti OR 'interleukins':ab,ti OR 'interleukin-10':ab,ti | 340239 |
| #20 | 'interleukin':ab,ti OR 'interleukins':ab,ti OR 'interleukin-8':ab,ti | 340239 |
| #19 | 'interleukin':ab,ti OR 'interleukins':ab,ti OR 'interleukin-6':ab,ti | 340243 |
| #18 | 'interleukin':ab,ti OR 'interleukins':ab,ti OR 'interleukin-1':ab,ti OR 'interleukin-1alpha':ab,ti OR 'interleukin-1beta':ab,ti | 340239 |
| #17 | 'inflammatory cytokines':ab,ti OR 'proinflammatory cytokines':ab,ti OR 'anti-inflammatory cytokine':ab,ti OR 'inflammatory factor':ab,ti OR 'cytokine':ab,ti OR 'proinflammatory factor':ab,ti | 460634 |
| #16 | #3 OR #15 | 23365 |
| #15 | #13 AND #14 | 20847 |
| #14 | 'sepsis':ab,ti OR 'bloodstream infection':ab,ti OR 'bloodstream infections':ab,ti OR 'infection, bloodstream':ab,ti OR 'pyemia':ab,ti OR 'pyemias':ab,ti OR 'pyohemia':ab,ti OR 'pyohemias':ab,ti OR 'pyaemia':ab,ti OR 'pyaemias':ab,ti OR 'septicemia':ab,ti OR 'septicemias':ab,ti OR 'poisoning, blood':ab,ti OR 'blood poisoning':ab,ti OR 'blood poisonings':ab,ti OR 'poisonings, blood':ab,ti OR 'severe sepsis':ab,ti OR 'sepsis, severe':ab,ti OR 'bacteremias':ab,ti OR 'bacteremia':ab,ti | 256948 |
| #13 | #4 OR #5 OR #6 OR #7 OR #8 OR #9 OR #10 OR #11 OR #12 | 582741 |
| #12 | 'infant, very low birth weight':ab,ti OR 'very-low-birth-weight infant':ab,ti OR 'infant, very-low-birth-weight':ab,ti OR 'infants, very-low-birth-weight':ab,ti OR 'very low birth weight infant':ab,ti OR 'very-low-birth-weight infants':ab,ti OR 'very low birth weight':ab,ti | 10795 |
| #11 | 'infant, extremely low birth weight':ab,ti | 2 |
| #10 | 'infant, low birth weight':ab,ti OR 'low-birth-weight infant':ab,ti OR 'infant, low-birth-weight':ab,ti OR 'infants, low-birth-weight':ab,ti OR 'low birth weight infant':ab,ti OR 'low-birth-weight infants':ab,ti OR 'low birth weight':ab,ti OR 'birth weight, low':ab,ti OR 'birth weights, low':ab,ti OR 'low birth weights':ab,ti | 43301 |
| #9 | 'infant, low birth weight':ab,ti OR 'low-birth-weight infant':ab,ti OR 'infant, low-birth-weight':ab,ti OR 'infants, low-birth-weight':ab,ti OR 'low birth weight infant':ab,ti OR 'low-birth-weight infants':ab,ti OR 'low birth weight':ab,ti OR 'birth weight, low':ab,ti OR 'birth weights, low':ab,ti OR 'low birth weights':ab,ti | 43301 |
| #8 | 'overdue birth':ab,ti OR 'normal birth weight infants':ab,ti OR 'extremely low birth weight infant':ab,ti OR 'giant':ab,ti OR 'suitable for gestational age':ab,ti OR 'large for gestational age':ab,ti OR 'early newborn':ab,ti OR 'late newborn':ab,ti OR 'high risk children':ab,ti | 128575 |
| #7 | 'term birth':ab,ti OR 'birth, term':ab,ti OR 'term births':ab,ti OR 'birth of full-term newborn':ab,ti OR 'birth of full term newborn':ab,ti OR 'fullterm birth':ab,ti OR 'birth, fullterm':ab,ti OR 'births, fullterm':ab,ti OR 'fullterm births':ab,ti OR 'birth of full-term infant':ab,ti OR 'birth of full term infant':ab,ti OR 'full-term infant births':ab,ti | 4604 |
| #6 | 'infant, extremely premature':ab,ti OR 'extremely premature infant':ab,ti OR 'infants, extremely premature':ab,ti OR 'premature infant, extremely':ab,ti OR 'premature infants, extremely':ab,ti OR 'extremely preterm infants':ab,ti OR 'extremely preterm infant':ab,ti OR 'infant, extremely preterm':ab,ti OR 'infants, extremely preterm':ab,ti OR 'preterm infant, extremely':ab,ti OR 'preterm infants, extremely':ab,ti OR 'extremely premature infants':ab,ti | 3672 |
| #5 | 'infant, premature':ab,ti OR 'infants, premature':ab,ti OR 'premature infant':ab,ti OR 'preterm infants':ab,ti OR 'infant, preterm':ab,ti OR 'infants, preterm':ab,ti OR 'preterm infant':ab,ti OR 'premature infants':ab,ti OR 'neonatal prematurity':ab,ti OR 'prematurity, neonatal':ab,ti | 67484 |
| #4 | 'infant, newborn':ab,ti OR 'infants, newborn':ab,ti OR 'newborn infant':ab,ti OR 'newborn infants':ab,ti OR 'newborns':ab,ti OR 'newborn':ab,ti OR 'neonate':ab,ti OR 'neonates':ab,ti | 381258 |
| #3 | #1 OR #2 | 7328 |
| #2 | 'neonatal sepsis':ab,ti | 7100 |
| #1 | 'neonatal sepses':ab,ti OR 'sepses, neonatal':ab,ti OR 'sepsis, neonatal':ab,ti OR 'neonatal late-onset sepsis':ab,ti OR 'late-onset sepses, neonatal':ab,ti OR 'late-onset sepsis, neonatal':ab,ti OR 'neonatal late onset sepsis':ab,ti OR 'neonatal late-onset sepses':ab,ti OR 'sepses, neonatal late-onset':ab,ti OR 'sepsis, neonatal late-onset':ab,ti OR 'neonatal early-onset sepsis':ab,ti OR 'early-onset sepses, neonatal':ab,ti OR 'early-onset sepsis, neonatal':ab,ti OR 'neonatal early onset sepsis':ab,ti OR 'neonatal early-onset sepses':ab,ti OR 'sepses, neonatal early-onset':ab,ti OR 'sepsis, neonatal early-onset':ab,ti | 369 |

The Cochrane Library（0）

IL-1（0）

IL-6（0）

IL-8（0）

IL-10（0）

TNF-α（0）

****
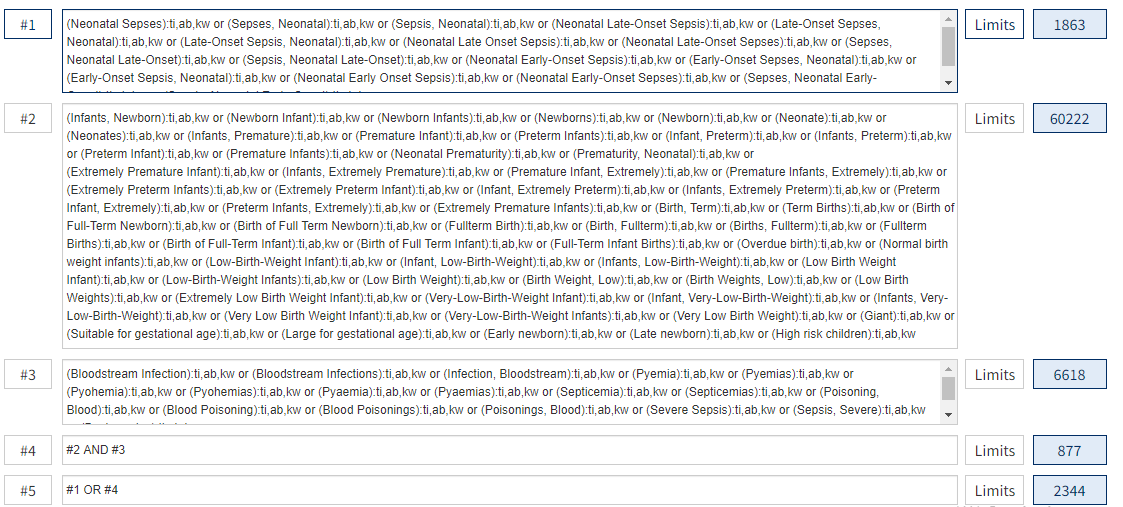
****

****
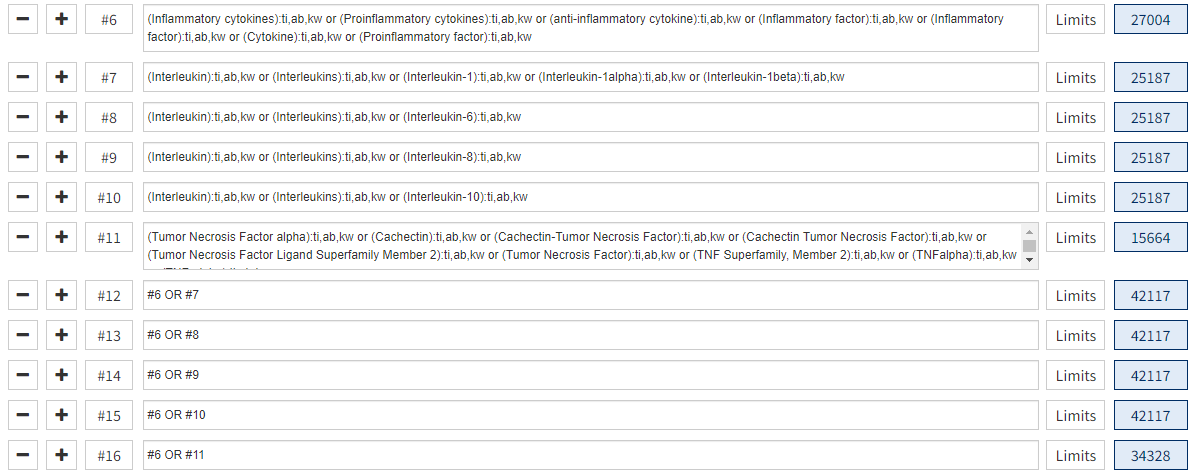
****

****
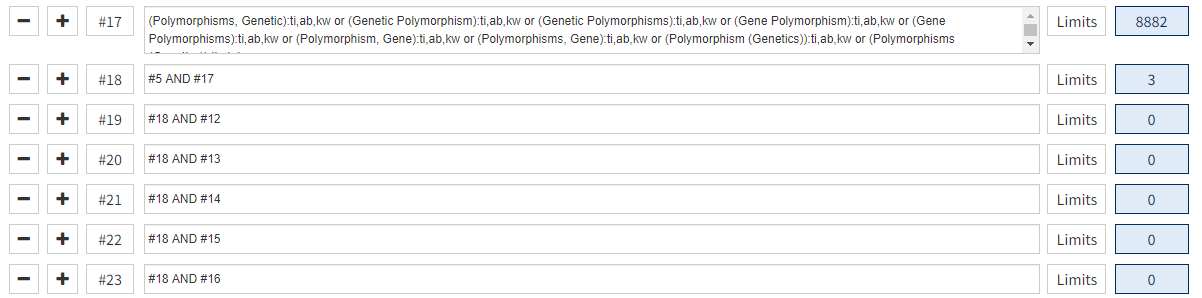
****

Web of Science

IL-1（133）

IL-6（133）

IL-8（133）

IL-10（133）

TNF-α（112）

28

**#16 AND #23** and **Preprint Citation Index** (Exclude – Database)

[112](http://gfbic892151ba9cff47cfsxw966q6bu5px6uf9.fiac.jiujiang.booktsg.com/wos/alldb/summary/d95a7d7e-29ee-4593-8a93-152690a26bb6-b60045ae/relevance/1)

Add to query

linkeditnotifications

27

**#16 AND #22** and **Preprint Citation Index** (Exclude – Database)

[133](http://gfbic892151ba9cff47cfsxw966q6bu5px6uf9.fiac.jiujiang.booktsg.com/wos/alldb/summary/ca93f4b7-baea-407d-999f-12a6156efcd9-b60040e4/relevance/1)

Add to query

linkeditnotifications

26

**#16 AND #21** and **Preprint Citation Index** (Exclude – Database)

[133](http://gfbic892151ba9cff47cfsxw966q6bu5px6uf9.fiac.jiujiang.booktsg.com/wos/alldb/summary/856b48c8-1b87-4b31-99fd-10ea19a30d66-b6003a54/relevance/1)

Add to query

linkeditnotifications

25

**#16 AND #19** and **Preprint Citation Index** (Exclude – Database)

[133](http://gfbic892151ba9cff47cfsxw966q6bu5px6uf9.fiac.jiujiang.booktsg.com/wos/alldb/summary/12b49cad-b246-44e8-a642-b09b79a1da8e-b6002ae8/relevance/1)

Add to query

linkeditnotifications

24

**#16 AND #18** and **Preprint Citation Index** (Exclude – Database)

[133](http://gfbic892151ba9cff47cfsxw966q6bu5px6uf9.fiac.jiujiang.booktsg.com/wos/alldb/summary/be5ac7d0-4f14-481a-a6fd-32bfe420e88e-b600239c/relevance/1)

Add to query

linkeditnotifications

23

**#8 OR #13** and **Preprint Citation Index** (Exclude – Database)

[1,277,165](http://gfbic892151ba9cff47cfsxw966q6bu5px6uf9.fiac.jiujiang.booktsg.com/wos/alldb/summary/4a4e1e99-fffb-4cfb-83f0-3d23106f12c0-b60002f5/relevance/1)

Add to query

linkeditnotifications

22

**#8 OR #12** and **Preprint Citation Index** (Exclude – Database)

[1,371,489](http://gfbic892151ba9cff47cfsxw966q6bu5px6uf9.fiac.jiujiang.booktsg.com/wos/alldb/summary/cc16e098-25a3-492d-ae1b-dce1e696c617-b5fffe58/relevance/1)

Add to query

linkeditnotifications

21

**#8 OR #11** and **Preprint Citation Index** (Exclude – Database)

[1,371,489](http://gfbic892151ba9cff47cfsxw966q6bu5px6uf9.fiac.jiujiang.booktsg.com/wos/alldb/summary/cfa744e4-6357-43a2-b079-df5b48ff1ea0-b5fffb03/relevance/1)

Add to query

linkeditnotifications

20

**lactic acid bacteria** (Topic) and **insect** (Topic) and **Preprint Citation Index** (Exclude – Database) and **2023** (Publication Years)

[58](http://gfbic892151ba9cff47cfsxw966q6bu5px6uf9.fiac.jiujiang.booktsg.com/wos/alldb/summary/c0d49254-e28b-4903-857a-6b93e1e9b3ad-b5fff70f/relevance/1)

Add to query

linkeditnotifications

19

**#8 OR #10** and **Preprint Citation Index** (Exclude – Database)

[1,371,489](http://gfbic892151ba9cff47cfsxw966q6bu5px6uf9.fiac.jiujiang.booktsg.com/wos/alldb/summary/0b7c9bad-159e-4d06-804a-96c9d232f7e9-b5fff3b6/relevance/1)

Add to query

linkeditnotifications

18

**#8 OR #9** and **Preprint Citation Index** (Exclude – Database)

[1,371,489](http://gfbic892151ba9cff47cfsxw966q6bu5px6uf9.fiac.jiujiang.booktsg.com/wos/alldb/summary/6bc48504-4153-438f-ad90-fd7cd6e74169-b5ffee6e/relevance/1)

Add to query

linkeditnotifications

17

**lactic acid bacteria** (Topic) and **insect** (Topic) and **Preprint Citation Index** (Exclude – Database)

[634](http://gfbic892151ba9cff47cfsxw966q6bu5px6uf9.fiac.jiujiang.booktsg.com/wos/alldb/summary/ad66415b-e46b-4446-a5b2-d07b3ac07fea-b5ffdddd/relevance/1)

Add to query

linkeditnotifications

16

**#5 AND #15** and **Preprint Citation Index** (Exclude – Database)

[608](http://gfbic892151ba9cff47cfsxw966q6bu5px6uf9.fiac.jiujiang.booktsg.com/wos/alldb/summary/6d8e38ed-7a92-418a-be1a-560ebc7f0fd5-b5ffc42c/relevance/1)

Add to query

linkeditnotifications

15

**#2 OR #14** and **Preprint Citation Index** (Exclude – Database)

[48,050](http://gfbic892151ba9cff47cfsxw966q6bu5px6uf9.fiac.jiujiang.booktsg.com/wos/alldb/summary/08e0fc99-fa98-46cb-b653-40f6b02ba01a-b5ffa9cd/relevance/1)

Add to query

linkeditnotifications

14

**#3 AND #4** and **Preprint Citation Index** (Exclude – Database)

[46,015](http://gfbic892151ba9cff47cfsxw966q6bu5px6uf9.fiac.jiujiang.booktsg.com/wos/alldb/summary/5cbbee78-35d4-4037-9e39-d4b331c8fc79-b5ff984a/relevance/1)

Add to query

linkeditnotifications

13

**TS=(Tumor Necrosis Factor-alpha or Tumor Necrosis Factor alpha or Cachectin or Cachectin-Tumor Necrosis Factor or Cachectin Tumor Necrosis Factor or Tumor Necrosis Factor Ligand Superfamily Member 2 or Tumor Necrosis Factor or TNF Superfamily, Member 2 or TNFalpha or TNF-alpha)** and **Preprint Citation Index** (Exclude – Database)

[481,716](http://gfbic892151ba9cff47cfsxw966q6bu5px6uf9.fiac.jiujiang.booktsg.com/wos/alldb/summary/ce041fde-c767-4635-b9d7-0ed28018e444-b5ff4d76/relevance/1)

Add to query

linkeditnotifications

12

**TS=(Interleukins or Interleukin or Interleukin-10)** and **Preprint Citation Index** (Exclude – Database)

[657,189](http://gfbic892151ba9cff47cfsxw966q6bu5px6uf9.fiac.jiujiang.booktsg.com/wos/alldb/summary/2b8274fe-02c1-4923-b8cf-e2ccb11fe8a3-b5ff3522/relevance/1)

Add to query

linkeditnotifications

11

**TS=(Interleukins or Interleukin or Interleukin-8)** and **Preprint Citation Index** (Exclude – Database)

[657,189](http://gfbic892151ba9cff47cfsxw966q6bu5px6uf9.fiac.jiujiang.booktsg.com/wos/alldb/summary/d79e7d4a-6459-4951-b69b-5acc83e84c8b-b5ff312e/relevance/1)

Add to query

linkeditnotifications

10

**TS=(Interleukins or Interleukin or Interleukin-6)** and **Preprint Citation Index** (Exclude – Database)

[657,189](http://gfbic892151ba9cff47cfsxw966q6bu5px6uf9.fiac.jiujiang.booktsg.com/wos/alldb/summary/8b1c239d-3d4a-4834-9062-80672a540fe4-b5ff08b7/relevance/1)

Add to query

linkeditnotifications

9

**TS=(Interleukins or Interleukin or Interleukin-1 or Interleukin-1alpha or Interleukin-1beta)** and **Preprint Citation Index** (Exclude – Database)

[657,189](http://gfbic892151ba9cff47cfsxw966q6bu5px6uf9.fiac.jiujiang.booktsg.com/wos/alldb/summary/f9f63b2b-fd41-4bf9-8d70-b98e3e982287-b5ff0435/relevance/1)

Add to query

linkeditnotifications

8

**TS=(Inflammatory cytokines or Proinflammatory cytokines or anti-inflammatory cytokine or Inflammatory factor or Cytokines or Cytokine or Proinflammatory factor )** and **Preprint Citation Index** (Exclude – Database)

[1,089,043](http://gfbic892151ba9cff47cfsxw966q6bu5px6uf9.fiac.jiujiang.booktsg.com/wos/alldb/summary/79e42e45-6a3c-4218-b9be-0af63929a7dc-b5feedec/relevance/1)

Add to query

linkeditnotifications

7

**rebound or regain** (Topic) and **diabetes** (Topic) and **obesity** (Topic) and **Preprint Citation Index** (Exclude – Database)

[809](http://gfbic892151ba9cff47cfsxw966q6bu5px6uf9.fiac.jiujiang.booktsg.com/wos/alldb/summary/5e366fc6-d745-40ae-9672-66c6ce416246-b5fec75e/relevance/1)

Add to query

linkeditnotifications

6

**rebound or regain** (Topic) and **diabetes** (Topic) and **Preprint Citation Index** (Exclude – Database)

[1,693](http://gfbic892151ba9cff47cfsxw966q6bu5px6uf9.fiac.jiujiang.booktsg.com/wos/alldb/summary/bfba6d02-476e-42c9-8f61-d414d1623d4a-b5fec407/relevance/1)

Add to query

linkeditnotifications

5

**TS=(Polymorphism, Genetic or Polymorphisms, Genetic or Genetic Polymorphism or Genetic Polymorphisms or Gene Polymorphism or Gene Polymorphisms or Polymorphism, Gene or Polymorphisms, Gene or Polymorphism (Genetics) or Polymorphisms (Genetics))** and **Preprint Citation Index** (Exclude – Database)

[575,377](http://gfbic892151ba9cff47cfsxw966q6bu5px6uf9.fiac.jiujiang.booktsg.com/wos/alldb/summary/a0bc82dd-da55-4f53-b8b2-c1cb783b9db6-b5fe9703/relevance/1)

Add to query

linkeditnotifications

4

**TS=(Sepsis or Bloodstream Infection or Bloodstream Infections or Infection, Bloodstream or Pyemia or Pyemias or Pyohemia or Pyohemias or Pyaemia or Pyaemias or Septicemia or Septicemias or Poisoning, Blood or Blood Poisoning or Blood Poisonings or Poisonings, Blood or Severe Sepsis or Sepsis, Severe or Bacteremia or Bacteremias)** and **Preprint Citation Index** (Exclude – Database)

[416,085](http://gfbic892151ba9cff47cfsxw966q6bu5px6uf9.fiac.jiujiang.booktsg.com/wos/alldb/summary/1f83e5d1-40af-4e6f-ac5f-4d75ad280bd6-b5fe8b9a/relevance/1)

Add to query

linkeditnotifications

3

**TS=(Infant, Newborn or Infants, Newborn or Newborn Infant or Newborn Infants or Newborns or Newborn or Neonate or Neonates or Infant, Premature or Infants, Premature or Premature Infant or Preterm Infants or Infant, Preterm or Infants, Preterm or Preterm Infant or Premature Infants or Neonatal Prematurity or Prematurity, Neonatal or Infant, Extremely Premature or Extremely Premature Infant or Infants, Extremely Premature or Premature Infant, Extremely or Premature Infants, Extremely or Extremely Preterm Infants or Extremely Preterm Infant or Infant, Extremely Preterm or Infants, Extremely Preterm or Preterm Infant, Extremely or Preterm Infants, Extremely or Extremely Premature Infants or Term Birth or Birth, Term or Term Births or Birth of Full-Term Newborn or Birth of Full Term Newborn or Fullterm Birth or Birth, Fullterm or Births, Fullterm or Fullterm Births or Birth of Full-Term Infant or Birth of Full Term Infant or Full-Term Infant Births or Overdue birth or Normal birth weight infants or Infant, Low Birth Weight or Low-Birth-Weight Infant or Infant, Low-Birth-Weight or Infants, Low-Birth-Weight or Low Birth Weight Infant or Low-Birth-Weight Infants or Low Birth Weight or Birth Weight, Low or Birth Weights, Low or Low Birth Weights or Infant, Extremely Low Birth Weight or Extremely Low Birth Weight Infant or Infant, Very Low Birth Weight or Very-Low-Birth-Weight Infant or Infant, Very-Low-Birth-Weight or Infants, Very-Low-Birth-Weight or Very Low Birth Weight Infant or Very-Low-Birth-Weight Infants or Very Low Birth Weight or Giant or Suitable for gestational age or Infant, Small for Gestational Age or Large for gestational age or Early newborn or Late newborn or High risk children)** and **Preprint Citation Index** (Exclude – Database)

[1,603,797](http://gfbic892151ba9cff47cfsxw966q6bu5px6uf9.fiac.jiujiang.booktsg.com/wos/alldb/summary/c021302b-10ee-4f4a-b37f-8f4bb1114d91-b5fe84b7/relevance/1)

Add to query

linkeditnotifications

2

**TS=(Neonatal sepsis or Neonatal Sepses or Sepses, Neonatal or Sepsis, Neonatal or Neonatal Late-Onset Sepsis o r Late-Onset Sepses, Neonatal or Late-Onset Sepsis, Neonatal or Neonatal Late Onset Sepsis or Neonatal Late-Onset Sepses or Sepses, Neonatal Late-Onset
or Sepsis, Neonatal Late-Onset or Neonatal Early-Onset Sepsis or Early-Onset Sepses, Neonatal or Early-Onset Sepsis, Neonatal or Neonatal Early Onset Sepsis or Neonatal Early-Onset Sepses or Sepses, Neonatal Early-Onset or Sepsis, Neonatal Early-Onset)** and **Preprint Citation Index** (Exclude – Database)

[18,140](http://gfbic892151ba9cff47cfsxw966q6bu5px6uf9.fiac.jiujiang.booktsg.com/wos/alldb/summary/13b2c0ba-99d2-4095-ae2c-398db7d40183-b5fe743c/relevance/1)

Add to query

linkeditnotifications

1

**Comparative Efficacy of Early TIPS, Non-Early TIPS, and Standard treatment in patients with cirrhosis and acute variceal bleeding: a network meta-analysis** (Topic) and **Preprint Citation Index** (Exclude – Database)

[1](http://gfbic892151ba9cff47cfsxw966q6bu5px6uf9.fiac.jiujiang.booktsg.com/wos/alldb/summary/25f06c86-7aa2-46a6-9a41-ef217468a849-b5fdb8be/relevance/1)
